# High‐Throughput Discovery of Ni(IN)_2_ for Ethane/Ethylene Separation

**DOI:** 10.1002/advs.202004940

**Published:** 2021-04-01

**Authors:** Minjung Kang, Sunghyun Yoon, Seongbin Ga, Dong Won Kang, Seungyun Han, Jong Hyeak Choe, Hyojin Kim, Dae Won Kim, Yongchul G. Chung, Chang Seop Hong

**Affiliations:** ^1^ Department of Chemistry Korea University Seoul 02841 Republic of Korea; ^2^ School of Chemical Engineering Pusan National University Busan 46241 Republic of Korea

**Keywords:** C2 separation, ethane‐selective MOFs, high‐throughput discovery, metal–organic frameworks, recyclability

## Abstract

Although ethylene (C_2_H_4_) is one of the most critical chemicals used as a feedstock in artificial plastic chemistry fields, it is challenging to obtain high‐purity C_2_H_4_ gas without any trace ethane (C_2_H_6_) by the oil cracking process. Adsorptive separation using C_2_H_6_‐selective adsorbents is beneficial because it directly produces high‐purity C_2_H_4_ in a single step. Herein, **Ni(IN)_2_** (HIN = isonicotinic acid) is computationally discovered as a promising adsorbent with the assistance of the multiscale high‐throughput computational screening workflow and Computation‐Ready, Experimental (CoRE) metal–organic framework (MOF) 2019 database. **Ni(IN)_2_** is subsequently synthesized and tested to show the ideal adsorbed solution theory (IAST) selectivity of 2.45 at 1 bar for a C_2_H_6_/C_2_H_4_ mixture (1:15), which is one of the top‐performing selectivity values reported for C_2_H_6_‐selective MOFs as well as excellent recyclability, suggesting that this material is a promising C_2_H_6_‐selective adsorbent. Process‐level simulation results based on experimental isotherms demonstrate that the material is one of the top materials reported to date for ethane/ethylene separation under the conditions considered in this work.

## Graphical abstract

High‐throughput computational screening is performed to discover C_2_H_6_‐selective metal–organic frameworks (MOFs) in the Computation‐Ready, Experimental (CoRE) MOF 2019 database. With a well‐matched pore environment, **Ni(IN)_2_** can selectively adsorb C_2_H_6_ over C_2_H_4_ with a high experimental C_2_H_6_/C_2_H_4_ selectivity of 2.45.

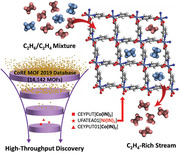

## Introduction

C_2_H_4_ (ethylene) is one of the most important olefins for the industrial mass production of commercial polymer materials and high valued chemicals.^[^ ^1^ ^]^ C_2_H_4_ is produced through a steam cracking process, where C_2_H_6_ (ethane) is the primary feedstock for the reaction. The effluent of the steam cracking process contains 5–10% of unreacted C_2_H_6_, which must be separated to obtain high‐purity C_2_H_4_ for the polymerization reaction. However, the similarity in molecular size and volatility of C_2_H_6_ (kinetic diameter 4.16 Å; boiling point 169.42 K) and C_2_H_4_ (kinetic diameter 4.44 Å; boiling point 184.55 K) makes the separation of the two molecules a challenge.^[^ ^2^ ^]^ To overcome the limitation of the technology, researchers have highly sought and intensively investigated effective separation methods, such as adsorption separation using zeolites and activated carbons, at near‐ambient temperature and pressure during the past decades.^[^ ^3^ ^]^

Metal–organic frameworks (MOFs) have attracted much attention for the separation of hydrocarbons because of their high pore volumes and designable pore properties.^[^ ^1^, ^4^ ^]^ For ethane/ethylene separation, most of the reported MOFs were C_2_H_4_ selective, where the separation mechanism typically involves the selective interaction of C_2_H_4_ with open metal sites or highly polar groups in the framework.^[^ ^5^ ^]^ However, these C_2_H_4_‐selective MOFs are not ideal because they demand an additional desorption procedure to afford the C_2_H_4_‐rich product stream (≈99.95%) and the harsh desorption conditions owing to their strong interactions.^[^ ^5^, ^6^ ^]^ In contrast, C_2_H_6_‐selective MOFs are more energy efficient because high‐purity C_2_H_4_ can be directly obtained using only a simple adsorption process to adsorb a trace amount of C_2_H_6_ molecules. Despite these advantages, this type of adsorption behavior has been reported in a few MOFs and hydrogen‐bonded organic frameworks so far, displaying relatively low C_2_H_6_ selectivity owing to the similar polarity of C_2_H_6_ and C_2_H_4_.^[^ ^7^ ^]^

The interaction between C_2_H_6_ molecules and a framework material can be tuned in MOFs by controlling the pore surface chemistry based on topology and organic ligands. Because C_2_H_4_ has a larger quadrupole moment (1.50 × 10^−26^ esu cm^2^) and a smaller polarization (42.52 × 10^−25^ cm^3^) than those of C_2_H_6_ (0.65 × 10^−26^ esu cm^2^ and 44.7 × 10^−25^ cm^3^, respectively),^[^ ^2a^ ^]^ the pore surface chemistry of MOFs can be fine‐tuned to enable the selective adsorption of C_2_H_6_ over C_2_H_4_. Separation of C_2_H_6_/C_2_H_4_ using MOFs with a suitable pore size is an effective strategy; however, discovering high‐performing MOFs with specific pore sizes is a formidable challenge because of the large number of reported MOFs in the literature.^[^ ^8^ ^]^

High‐throughput computational screening can supplement the experimental efforts to discover MOFs for C_2_H_6_/C_2_H_4_ separation. Keskin et al. computationally screened 278 MOFs using molecular simulation and compared the adsorption selectivities and working capacities of MOFs with those of zeolites.^[^ ^9^ ^]^ Jiang et al. applied computational screening to a large set (12020) of MOFs and found 16 top‐performing MOFs for C_2_H_6_/C_2_H_4_ separation. On the basis of the extensive data obtained from the screening, they established the relationships between performance metrics such as working capacity, selectivity, and structural features such as pore size and surface area.^[^ ^10^ ^]^ However, there is a growing body of evidence indicating that the performance metrics commonly used in the literature, such as working capacity and selectivity, are inadequate to correctly predict the material's performance in actual process settings, such as in pressure vacuum‐swing adsorptions. Recent studies suggest purity and recovery as more meaningful indicators of adsorbent materials for separation. For instance, Rodrigues et al. employed purity and recovery to assess the C_2_H_6_/C_2_H_4_ separation performance.^[^ ^11^ ^]^ They evaluated the C_2_H_6_/C_2_H_4_ separation capabilities of Cu‐BTC (BTC = benzene‐1,3,5‐tricarboxylate) and ZIF‐8 (ZIF = zeolitic imidaxzolate framework) for pressure‐swing adsorption and simulated moving bed using purity, recovery, and productivity. Although process‐level evaluation is a more appropriate method for assessing material's performance for adsorption‐based separation, computational, or experimental evaluation of all the available MOFs at the process level is not practical due to the high cost and time involved with the task. Therefore, an alternative strategy is needed to discover MOFs with high ethane‐selective MOFs.

In this work, we combine high‐throughput computational screening with process simulation to computationally screen the Computation‐Ready, Experimental (CoRE) MOF 2019 database^[^ ^8b^ ^]^ for the selective adsorption of C_2_H_6_ over C_2_H_4_. Through computational screening, we found a top‐performing MOF, UFATEA01[**Ni(IN)_2_**], which we synthesized in the laboratory for testing. **Ni(IN)_2_** (HIN = isonicotinic acid) is a nickel isonicotinate‐based ultra‐microporous MOF with high stability under humid conditions. In addition, **Ni(IN)_2_** can be synthesized on a gram scale.^[^ ^12^ ^]^ The experimental data of **Ni(IN)_2_** and other adsorbent data from recently reported studies were compared according to the ideal vacuum swing adsorption (VSA) process modeling. The results of process‐level simulations suggest that **Ni(IN)_2_** is the top‐performing material used in computational screening and is superior to many of the adsorbent materials reported to date for C_2_H_4_/C_2_H_6_ separation.

## Results and Discussion

**Figure 1:**
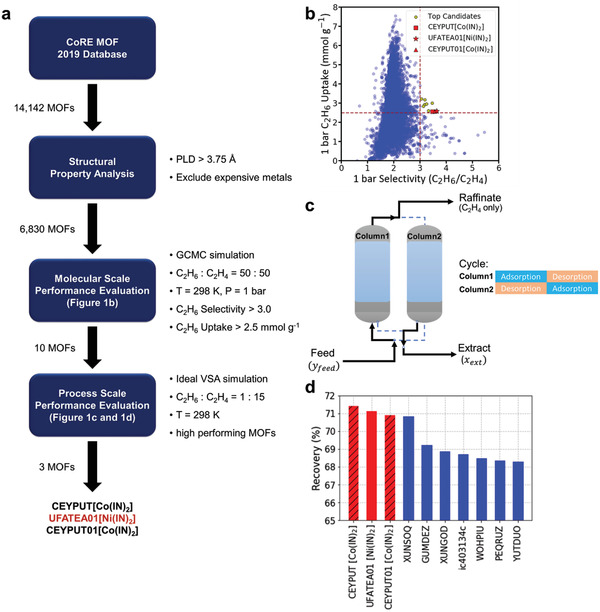
a) Screening workflow for identifying high‐performing MOFs from the CoRE MOF 2019 database. b) C_2_H_6_ uptake versus selectivity (C_2_H_6_ uptake/C_2_H_4_ uptake) at 1 bar for 6830 structures. Each point corresponds to the GCMC result from a single MOF structure. c) Operation diagram of an ideal VSA process simulation. d) C_2_H_4_ recovery in an ideal VSA process simulation with a 0.01 mbar vacuum operation with top three MOFs (orange bars) and excluded ones due to their durabilities (hatched bars).

As shown in **Figure** **1a**, the high‐performing MOFs (CEYPUT[**Co(IN)_2_**], UFATEA01[**Ni(IN)_2_**], and CEYPUT01[**Co(IN)_2_**]) were selected according to the screening procedure consisting of three different filters. In the first filter, we excluded MOFs with a pore limiting diameter (PLD) smaller than 3.75 Å, which corresponds to the diameter of a methyl group of C_2_H_6_ from the TraPPE model. Because C_2_H_6_ molecules are difficult to access in the pores of MOFs with a PLD smaller than 3.75 Å, materials with smaller pore sizes are screened out. Consecutively, we excluded MOFs containing expensive metal atoms (Au, Ag, Dy, Eu, Ga, Gd, Hf, In, Ir, La, Mo, Nd, Pd, Pr, Pt, Rh, Ru, Se, Sm, Tb, Te, Tm, U, and Y). This exclusion was employed as the second part of the first filter. Grand canonical Monte Carlo (GCMC) simulations were performed for the remaining 6830 MOFs to evaluate their performances. These simulations computed the binary component (50:50) adsorption uptake for C_2_H_6_ and C_2_H_4_ in the 6830 MOFs. More detailed methods are discussed in Section S1 in the Supporting Information. Figure 1b presents the distribution of both C_2_H_6_ uptake and selectivity (C_2_H_6_ uptake/C_2_H_4_ uptake) at 1 bar obtained from the GCMC simulations. From the computational screening data, we selected MOFs with a selectivity larger than 3 and a C_2_H_6_ uptake larger than 2.5 mmol g^–1^ because selectivity and uptake have been considered as critical metrics in many previous studies.^[^ ^7a^ ^‐^ ^7^, ^13^ ^]^ This step reduced the number of target MOFs from 6830 to 10, resulting in a significant reduction in the computational cost for the subsequent process modeling. Detailed discussion on the selection strategy is discussed in Section S6 in the Supporting Information.

Recent discussions in the literature^[^ ^14^ ^]^ show that the widely popular adsorbent's performance metrics, such as selectivity and working capacity, do not accurately represent the materials' process‐level performance. For the process‐level performance, product (i.e., C_2_H_4_) purity and recovery are two main performance metrics. In this work, the ethylene recovery, which is directly related to the amount of ethylene produced from the process, was calculated based on the ideal VSA simulation^[^ ^15^ ^]^ for the selected ten MOFs to evaluate their process‐level performance. Several modifications to the original method were made to enable the process‐level performance evaluation for the C_2_H_6_/C_2_H_4_ separation case, and the details are discussed in Section S1 in the Supporting Information. Figure 1d shows the ideal VSA simulation results for the ten selected MOFs. The results show that the top three MOFs (CEYPUT (**Co(IN)_2_**), CEPUT01, UFATEA01 (**Ni(IN)_2_**)) are the same MOFs but synthesized with different metals, suggesting that the material's performance depends on the identity of the ligand that forms the favorable pore environment for selective C_2_H_6_ adsorption.

To validate the performance of **Co(IN)_2_** and **Ni(IN)_2_**, we synthesized and tested the adsorption performance of the two MOFs. **Co(IN)_2_** and **Ni(IN)_2_** were prepared according to the procedure reported in the literature^[^ ^12^, ^16^ ^]^ and characterized by powder X‐ray diffraction (PXRD) patterns (Figures S3 and S4, Supporting Information). The PXRD patterns of the synthesized MOFs matched well with those of the simulated structures, confirming their high phase purity. Before the gas isotherm measurements, the MOFs were degassed at 160 °C for 12 h under a vacuum to remove the guest molecules from the pores. However, the peaks of **Co(IN)_2_** almost disappeared in the PXRD pattern, indicating a structural collapse during the degassing process (Figure S3, Supporting Information). On the other hand, **Ni(IN)_2_** retained its structure after not only the degassing process but also the various gas adsorption measurements (Figure S4, Supporting Information). Because structural stability is vital in industrial applications, **Ni(IN)_2_** was chosen as a target adsorbent among potential adsorbent candidates.

**Figure 2:**
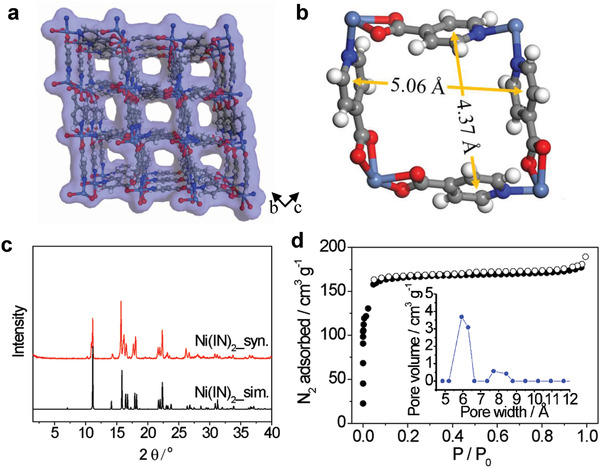
a) A 3D porous framework with a Connolly surface representation of **Ni(IN)_2_** with microporous 1D channels along the *a*‐axis. b) Pore window based on the distances between the centroids of the pyridine rings in the channel from the crystal structure. c) PXRD patterns of the simulated and as‐synthesized **Ni(IN)_2_**. d) N_2_ adsorption isotherm of **Ni(IN)_2_** at 77 K. The inset indicates the pore size distribution in the range of 5–12 Å.

As shown in **Figure** **2a**,b, **Ni(IN)_2_** has a 1D channel with a size of 5.06 × 4.37 Å (excluding the van der Waals radii) along the *a*‐axis. The framework is composed of linking isolated Ni octahedra by IN^–^ ligands, affording a square lattice coordination network. The synthesized **Ni(IN)_2_** was characterized by several experimental methods (Figure 2 and Figures S2, S4, and S5–S7, Supporting Information). The PXRD pattern was well matched with the simulated one, confirming that the MOF was successfully prepared with high purity (Figure 2c). The nitrogen isotherm of **Ni(IN)_2_** was collected at 77 K (Figure 2d). The isotherm with a typical type I behavior indicates the dominance of microporosity. The Brunauer–Emmett–Teller surface area and the total pore volume of **Ni(IN)_2_** were calculated to be 520 m^2^ g^–1^ and 0.305 cm^3^ g^–1^, respectively. Furthermore, from the result of the density functional theory analysis, the pore size distribution was in the range 5–12 Å and mainly peaked at 6 Å, which is consistent with the pore size estimated from the crystal structure (Figure 2b).

**Figure 3:**
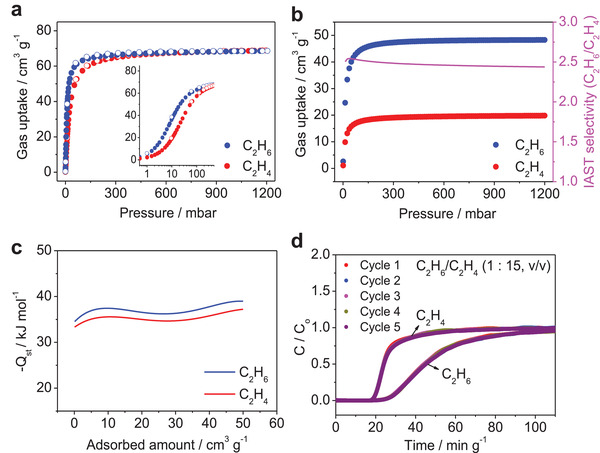
a) C_2_H_6_ and C_2_H_4_ adsorption isotherms of **Ni(IN)_2_** at 298 K. The inset graph is plotted on a log scale. b) Adsorption isotherms and selectivity of **Ni(IN)_2_** predicted by the IAST model for a gas mixture of C_2_H_6_/C_2_H_4_ (1:1, v/v) at 298 K. c) Plots of isosteric heat of adsorption of C_2_H_6_ and C_2_H_4_ for **Ni(IN)_2_**. d) Cycling breakthrough curves of the C_2_H_6_/C_2_H_4_ mixture (1:15, v/v) in a fixed bed packed with **Ni(IN)_2_** at 298 K and 1 bar.

The accessible surface of the channels is surrounded by pyridine rings of the IN^–^ ligands, which is expected to be a favorable structure for the framework to have host–guest interactions with incoming gas molecules. Single‐component C_2_H_6_ and C_2_H_4_ isotherms of **Ni(IN)_2_** were recorded at 298 K and different temperatures after being fully activated at 160 °C under vacuum for 12 h to remove the guest molecules from the pores. These isotherms exhibited a type I behavior with a steep adsorption trend from 0 to 0.1 bar in **Figure** **3a**. This indicates that C_2_H_6_ and C_2_H_4_ have strong host‐guest interactions with the backbone of **Ni(IN)_2_**. As the adsorption temperature increased from 273 to 323 K, the pressure of the steep adsorption events also increased levels (Figures S8 and S9, Supporting Information). The C_2_H_6_ uptake capacity of **Ni(IN)_2_** at 1 bar (68.36 cm^3^ g^–1^) is notably superior to that of the benchmark a dsorbents MAF‐49 (38.1 cm^3^ g^–1^) (MAF = metal‐azolate framework), Cu(Qc)_2_ (41.4 cm^3^ g^–1^), and ZIF‐7 (41.1 cm^3^ g^–1^).^[^ ^7e,k^ ^]^

Ideal adsorbed solution theory (IAST) calculations were performed using the IAST++ program to predict the C_2_H_6_/C_2_H_4_ selectivity.^[^ ^17^ ^]^ The experimental adsorption isotherms of **Ni(IN)_2_** were fitted with the dual‐site Langmuir–Freundlich model and the adsorption selectivity of the binary mixture C_2_H_6_/C_2_H_4_ at ratios of 1:1 v/v and 1:15 v/v at 298 K was calculated from the IAST. The estimated selectivities of **Ni(IN)_2_** for C_2_H_6_/C_2_H_4_ at 298 K and 1 bar were 2.44 and 2.45 at ratios of 1:1 and 1:15, respectively (Figure 3b and Figure S10, Supporting Information). Among the reported MOFs, the selectivity of **Ni(IN)_2_** at 298 K and 1 bar at a ratio of 1:1 outperformed that of most C_2_H_6_‐selectivity MOFs such as MUF‐15 (1.96) (MUF = Massey university framework), MIL‐53(Al)‐FA (1.9) (MIL = Matériaux de l′Institut Lavoisier; FA = fumarate), Ni(TMBDC)(DABCO)_0.5_ (1.98) (H_2_TMBDC = 2,3,5,6‐tetramethylterephthalic acid; DABCO = 1,4‐diazabicyclo[2.2.2]octane), and ZIF‐7 (1.5).^[^ ^5^, ^7^ ^]^ The adsorption selectivities of the simulated structures calculated from binary component GCMC results were 3.65 and 2.91 for the pristine and relaxed structures, respectively (Table S4, Supporting Information). Using the Clausius–Clapeyron formula, we experimentally determined the isosteric heats of adsorption (−*Q*_st_) of **Ni(IN)_2_** for C_2_H_6_ and C_2_H_4_ from adsorption isotherms measured at 273, 298, and 323 K (Figure 3c and Figure S11 and Table S1, Supporting Information). The obtained −*Q*_st_ for C_2_H_6_ at zero coverage is 34.5 kJ mol^–1^, which is greater than that of 33.3 kJ mol^–1^ for C_2_H_4_. A similar tendency is observed for a higher coverage.

To explore the dynamic separation capability of the C_2_H_6_/C_2_H_4_ mixture, we performed a breakthrough experiment using a packed column containing ≈1 g of activated **Ni(IN)_2_**. As shown in Figure S12 in the Supporting Information, it is clear that **Ni(IN)_2_** can effectively separate a mixture of C_2_H_6_/C_2_H_4_ (1:1, v/v). C_2_H_4_ was initially passed through the column at 7.74 min g^–1^ and it reached saturation, while C_2_H_6_ was first detected at 11.79 min g^–1^. In the case of a different composition of C_2_H_6_/C_2_H_4_ (1:15, v/v), **Ni(IN)_2_** can also separate the mixed gas at 17.03 min g^–1^ for C_2_H_4_ and at 21.94 min g^–1^ for C_2_H_6_ (Figure S12, Supporting Information). The results showed that C_2_H_6_ is more efficiently adsorbed into the **Ni(IN)_2_** bed. The saturation uptakes of C_2_H_4_ and C_2_H_6_ on the fixed bed corresponded to 23.9  and 3.16 cm^3^ g^–1^, respectively, which is consistent with the expected amount based on the single‐component isotherms (17.6  and 2.88 cm^3^ g^–1^, respectively). The calculated selectivity from the breakthrough curves is ≈1.98, which is slightly less than that from IAST (2.44). Subsequently, we repeated the breakthrough tests five times for the C_2_H_6_/C_2_H_4_ mixture (1:15, v/v). After the first cycle, the adsorbent was regenerated completely within 2 h at 160 °C. The results of the recycling experiments showed that the breakthrough performance remained almost unchanged over five continuous cycles, suggesting that it has excellent recyclability for separation (Figure 3d). Additional experimental and simulation data, such as N_2_ isotherms and C_2_H_6_/C_2_H_4_ isotherms with molecular dynamics (MD) simulation results, are available in Figures S13–S15 and Tables S2 and S3 in the Supporting Information. Simulated results for N_2_, C_2_H_4_, and C_2_H_6_ indicate type I isotherm generated from the structure (Figures S14 and S15, Supporting Information). To reflect more realistic conditions, the relaxed **Ni(IN)_2_** structure was obtained from the pristine **Ni(IN)_2_** structure by MD simulation. The simulated isotherms from the relaxed **Ni(IN)_2_** are more similar to the experimental data than from the pristine **Ni(IN)_2_**.

**Figure 4:**
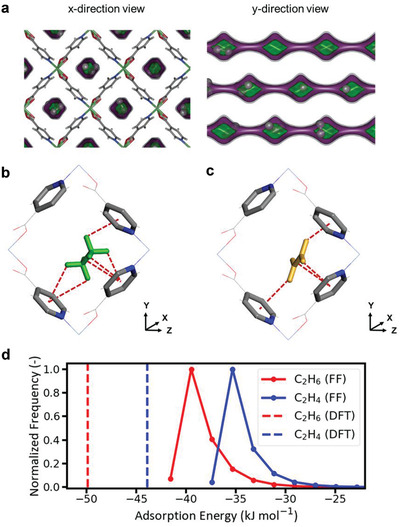
a) **Ni(IN)_2_** with methane energy isocontour levels: 0 (gray), −12.5 kJ mol^−1^ (purple), and −24.9 kJ mol^−1^ (green). A snapshot of the result of the binary mixture GCMC simulation of the C_2_H_6_ molecules is shown with the energy contour graph. For clarity, framework atoms were removed for the *y*‐direction view. b,c) Configurations of C_2_H_6_ and C_2_H_4_ in **Ni(IN)_2_** from DFT calculations. For visualization purposes, C_2_H_6_ and C_2_H_4_ molecules were represented by green and orange colors, respectively. The red dashed lines indicate C–H···*π* interactions. d) Adsorption energy distribution of a single C_2_H_6_ or C_2_H_4_ molecule inside **Ni(IN)_2_** obtained from force field‐based calculations (solid lines). The dashed vertical lines are the binding energy of C_2_H_6_ and C_2_H_4_ in **Ni(IN)_2_** obtained from DFT calculations.

The result of the potential energy surface analysis shows the unique pore environment of **Ni(IN)_2_**, which leads to the selective adsorption of C_2_H_6_ over C_2_H_4_. **Figure** **4a** shows the strong adsorption pockets within **Ni(IN)_2_** created by the ligand along the pore wall and that the size of each pocket can only accommodate a single C_2_H_6_ molecule. Periodic density functional theory (DFT) calculations were carried out to investigate the selective adsorption mechanism of C_2_H_6_ over C_2_H_4_. Figure 4b,c shows the optimized configurations of C_2_H_6_ and C_2_H_4_ in **Ni(IN)_2_**. The unique pore environment of **Ni(IN)_2_** enables all hydrogens of C_2_H_6_ and C_2_H_4_ to interact with the pyridine group of **Ni(IN)_2_**, where C_2_H_6_ has more C—H···*π* interactions than C_2_H_4_, which leads to more favorable interaction with the framework. The lack of C—H···*π* interactions between the pore wall and C_2_H_4_ molecule results in the lower DFT binding energy of C_2_H_6_ (−49.9 kJ mol^–1^) than C_2_H_4_ (−43.9 kJ mol^–1^). Figure 4d shows the adsorption energy distribution of C_2_H_6_ and C_2_H_4_ in the pores of **Ni(IN)_2_** obtained from Monte Carlo sampling of the pore with a single C_2_H_6_ or C_2_H_4_ molecule. The simulation data suggest that there is only one dominant adsorption energy peak for each molecule. This is consistent with the result of the potential energy surface analysis shown in Figure 4a, which shows a single strong adsorption site within the pores of **Ni(IN)_2_**. The adsorption energy of C_2_H_6_ obtained from the simulation shows that the dominant adsorption energy peak for C_2_H_6_ is −39.5 kJ mol^−1^, while that for C_2_H_4_ is −35.3 kJ mol^−1^, providing quantitative evidence for the origin of the C_2_H_6_‐selective nature of the material.

**Figure 5:**
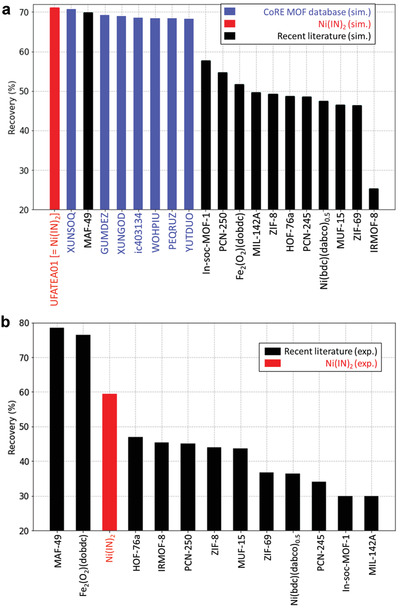
Results of the adsorbent evaluation (in terms of C_2_H_4_ recovery) based on the ideal VSA process simulations where 0.01 mbar desorption is used for the volumetric C_2_H_6_/C_2_H_4_ feed ratio of 1:15 at 298 K and 1 bar. a) Based on the molecular simulation results of the top adsorbent (**Ni(IN)_2_**) obtained from Figure 1d screening of the CoRE MOF 2019 database (red), of the other top candidates in Figure 1d screening work (blue), and of the recently reported adsorbents (black). b) Based on the experimental isotherm data of **Ni(IN)_2_** (red) and of the reported adsorbents (black).

The ideal VSA process simulations were performed to evaluate the performance of **Ni(IN)_2_** in a practical setting. **Figure** **5a** shows the results from the VSA process simulations based on the pure component adsorption isotherms obtained from the GCMC simulations. Besides the MOFs listed in Figure 1d (colored in blue both in Figure 1d and in Figure 5a), we also evaluated additional high‐performing materials for ethane/ethylene separation reported in the literature for comparison with **Ni(IN)_2_**. The results show **Ni(IN)_2_** is the best performing MOFs in terms of ethylene recovery. Recovery is a more realistic performance metric for the adsorbent process than productivity, a commonly used performance metric in recent literature. Additional results and discussions related to productivity and recovery metrics are provided in Section S8 of the Supporting Information. Furthermore, the experimental isotherms of the high‐performing MOFs reported in the literature were also used to carry out the ideal VSA process simulations (Figure 5b; colored in black). We found that the **Ni(IN)_2_** is among the top‐performing adsorbent materials for ethane/ethylene separation. Additional results and discussions related to different operating conditions are provided in Tables S4–S6 and Figures S16–S18 in Supporting Information.^[^ ^18^ ^]^

## Conclusion

In conclusion, we carried out high‐throughput computational screening and process‐level evaluation to discover C_2_H_6_‐selective MOFs from the CoRE MOF 2019 database. We explored a large number of MOFs to search for the most effective adsorbent for C_2_H_6_/C_2_H_4_ separation and successfully found an adsorbent, **Ni(IN)_2_**, that exhibits high performance in the separation process. **Ni(IN)_2_** with a well‐matched pore environment can selectively adsorb C_2_H_6_ over C_2_H_4_ with a high experimental C_2_H_6_/C_2_H_4_ selectivity of 2.45. The results of the breakthrough experiments with a mixture of C_2_H_6_/C_2_H_4_ (1:1 and 1:15, v/v) revealed that C_2_H_6_ was selectively separated from the gas mixture. The separation performance was well maintained over five repeated cycles. Using the process simulation, we compared the process‐level performance of **Ni(IN)_2_** with those of the recently reported MOFs. The result of this comparative evaluation shows that **Ni(IN)_2_** outperforms many of the adsorbents and ranks third place among the ten high‐performing MOFs. On the basis of these results, this work suggests that the MOF has a strong potential as a solid adsorbent for the separation of olefin/paraffin in industrial environments.

## Conflict of Interest

The authors declare no conflict of interest.

## Supporting information

Supporting Information

## Data Availability

Research data are not shared.
