# Supplementary material for: High‐Throughput Discovery of Ni(IN)2 for Ethane/Ethylene Separation
Source: Adv Sci (Weinh). 2021 Apr 1;8(11):2004940. doi: 10.1002/advs.202004940 (PMC8188204; doi:10.1002/advs.202004940)
Supplement: Supplementary file 1 — Supporting Information [file ADVS-8-2004940-s001.pdf]

© 2021 Wiley-VCH GmbH

ADVANCED SCIENCE
Open Access

Supporting Information

for *Adv. Sci.*, DOI: 10.1002/advs.202004940

High-Throughput Discovery of Ni(IN)2 for Ethane/Ethylene Separation

Minjung Kang[a], Sunghyun Yoon[b], Seongbin Ga[b], Dong Won Kang[a], Seungyun Han[b], Jong Hyeak Choe[a], Hyojin Kim[a], Dae Won Kim[a], Yongchul G. Chung,[b] and Chang Seop Hong*[a]*

Electronic Supplementary Information

High-Throughput Discovery of $Ni(IN)_2$ for Ethane/Ethylene Separation

Minjung Kang[a], Sunghyun Yoon[b], Seongbin Ga[b], Dong Won Kang[a], Seungyun Han[b], Jong Hyeak Choe[a], Hyojin Kim[a], Dae Won Kim[a], Yongchul G. Chung,*[b] and Chang Seop Hong*[a]

[a]Department of Chemistry, Korea University, Seoul 02841, Republic of Korea

[b]School of Chemical Biomolecular Engineering, Pusan National University, 46241 Busan, Korea (South)

E-mail: cshong@korea.ac.kr

E-mail: drygchung@gmail.com

S1. Materials and Methods

S2. Analytical characterizations

S3. Adsorption studies

S4. Comparison of experimental and simulated results

S5. Ideal VSA simulations results

S6. Correlation of process-level and molecular-level metrics

S7. Parameter estimations for isotherm models

S8. Comparison between productivity and recovery metrics

S9. References

S1. Materials and Methods

Materials: All chemicals and solvents employed in the synthesis were purchased from commercial suppliers and utilized without further purification.

Synthesis of [Co(IN)$_2$]: **Co(IN)$_2$** was prepared according to the reported procedure.[1] Cobalt nitrate hexahydrate (58.2 mg, 0.2 mmol), 4-pyridine carboxylic acid (HIN) (24.6 mg, 0.2 mmol), and dimethyl formamide (DMF) (10 mL) were mixed and sealed in a 25 mL solvothermal vial. After reaction at 140 ^{o}C for 72 h, purple block-shaped product was collected by filtration. The product was washed with freash DMF and methanol several times.

Synthesis of [Ni(IN)$_2$]: **Ni(IN)$_2$** was prepared according to the reported procedure.[2] Nickel acetate tetrahydrate (0.249 g, 1.0 mmol) and HIN (0.246 g, 2.0 mmol) in a mixture of DMF (6 mL) and acetonitrile (ACN) (4 mL) were stirred for 30 min at room temperature. After stirring the mixture, trimethylamine (0.075 mL) was added to it. The mixture was stirred for another 30 min to make it homogeneous. The mixture was placed in a 25 mL solvothermal vial and heated at 150 ^{o}C for 72 h after sealed. The bluish green product was collected by filtration and thoroughly washed with fresh DMF and methanol. The final product was isolated by filtration, washed with methanol, and dried under vacuum. Yield: 359 mg (93.3%). Elemental analysis (%) calcd. for $C_{15}H_{16}N_3NiO_{5.5}$ (**Ni(IN)$_2$**·DMF·0.5H_2O): C 46.80, N 10.91, H 4.19; found: C 46.60, N 10.84, H 4.11.

Gas sorption measurements: Before sorption analysis, the samples were activated at 160 ^{o}C under vacuum for 12 h. The composition of the activated sample was determined: elemental analysis (%) calcd. for for $C_{12}H_{8.8}N_2NiO_{4.4}$ (**Ni(IN)$_2$**·0.4H_2O): C 46.48, N 9.03, H 2.86; found: C 46.45, N 9.10, H 2.65. (Moisture content is due to exposure to air during elemental analysis measurements.) All gases used in measurements were highly pure (99.999%). Gas sorption was performed using a Micromeritics ASAP instrument up to 1 atm of gas pressure. Filled and open symbols indicate adsorption and desorption, respectively.

Physical measurements: Thermogravimetric analysis (TGA) was carried out in N_2 (99.999%) atmosphere (flow rate = 70 mL min^{-1}) in the temperature range 30 – 900 ^{o}C (heating rate = 2 ^{o}C min^{-1}) using a TA instrument Discovery TGA. Powder X-ray diffraction (PXRD) patterns were recorded using Cu Kα radiation (λ = 1.5406 Å) with a Rigaku Ultima III diffractometer with a scan speed of 2° min^{-1} and a step size of 0.01^{o}. Elemental analysis for C, H, and N was conducted at the Elemental Analysis Service

Center of Sogang University. Infrared (IR) spectra were obtained with an ATR module using a Nicolet iS10 FT-IR spectrometer.

Breakthrough experiments: The breakthrough curves of mixed gas C_2H_6/C_2H_4 (1:1 or 1:15, v/v) for **Ni(IN)$_2$** were performed by a BELCAT-II linked with BELMASS mass spectrometer. A fixed-bed column filled with the pelletized samples (425 – 600 μm) was prepared and the total gas flow rate was fixed to total 100 sccm for 1:1, and 50 sccm for 1:15. The flow rates of binary mixutre C_2H_6/C_2H_4 were controlled at 2 sccm for 1:1, and 1 sccm for 1:15, respectively, balanced with He gas. Before breakthrough measurements, all samples were activated at 160 ^oC for 12 h. The composition of the activated sample was determined: elemetal analysis (%) calcd. for for $C_{12}H_{9.7}N_2NiO_{4.85}$ (**Ni(IN)$_2$**$\cdot 0.85H_2O$): C 45.29, N 8.80, H 3.07; found: C 45.01, N 8.69, H 2.71. (Moisture content is due to exposure to air during elemental analysis measurements.) All outgas were continuously monitored by the mass spectrometer. The adsorbed amount of each gas was calculated with the ChemMaster program.[3] In case of the cyclic test, the samples were regenerated at 160 ^oC for 2 h before each cycle.

Computational methods: For molecular-level evaluation, the grand canonical Monte Carlo (GCMC) simulations were carried out to compute binary component (50:50) adsorption data for C_2H_6 and C_2H_4 in 6,830 MOFs from CoRE MOF 2019 database[4] at 1 bar, and 298 K. The single and binary component (50:50) adsorption isotherms were computed for C_2H_6 and C_2H_4 in top candidates (10 top-performing MOFs) in high-throughput computational screening with a range of pressure (from 1 Pa up to 100,000 Pa) at 298 K. Each GCMC simulation composed of a total 10,000 cycles where the first 5,000 cycles for initialization and the remaining 5,000 cycles for production run to compute ensemble average. The additional GCMC simulations were performed for 10,000 initialization cycles followed by 10,000 production cycles to compute single component C_2H_6 and C_2H_4 adsorption isotherms for the reported MOFs in the literature. The swap (insertion and deletion), rotation, re-insertion, translation, and identity change moves for C_2H_6 and C_2H_4 were considered equal probabilities for binary component GCMC simulations. For single-component GCMC simulations, the identity change move was not included in calculations. The potential energy surface of the materials were calculated by placing a single adsorbate molecule inside the unit cell and using MC moves to sample the inside of the framework. Energy histograms were computed based on 5,000,000 MC cycles. The Widom particle insertion simulations with 20,000 cycles were carried out at 298 K for top candidates (10 top-performing MOFs + 12 reported MOFs in the literature) in high-throughput computational screening to predict the heat of adsorption of C_2H_6 and C_2H_4 in the MOFs. Additionally, the Widom particle insertion simulations of 25,000 cycles were carried out at 298 K for C_2H_6 and C_2H_4 in the empty box (30 Å x 30 Å x 30 Å size) to predict the

internal energy of C_2H_6 and C_2H_4. The heat of adsorption was computed using the following equation (Eq. S1):

$$\Delta H = \langle U_{hg} \rangle - \langle U_h \rangle - \langle U_g \rangle - RT \quad \text{(Eq. S1)}$$

where ΔH is the heat of adsorption of the adsorbate (C_2H_6 and C_2H_4) in the framework (MOFs), $\langle U_{hg} \rangle$ is the interaction energy between the adsorbate and the framework, $\langle U_h \rangle$ is the framework energy (which is set to zero for rigid framework), $\langle U_g \rangle$ is the adsorbate's internal energy (which is zero for rigid molecules), R is gas constant, and T is the temperature of the system in Kelvin. For the N_2 adsorption isotherm in **Ni(IN)$_2$** with a range of pressure (up to 1 bar) at 77 K, the GCMC simulation was performed for 5,000 initialization cycles followed by 5,000 production cycles.

The Lennard-Jones (LJ) 12-6 potential was used to model the nonbonded interactions between framework atoms and atoms of adsorbates (Eq. S2):

$$U_{ij}(r_{ij}) = 4\varepsilon_{ij}\left[\left(\frac{\sigma_{ij}}{r_{ij}}\right)^{12} - \left(\frac{\sigma_{ij}}{r_{ij}}\right)^{6}\right] \quad \text{(Eq. S2)}$$

where U_{ij} is the interaction energy between atom i and j, r_{ij} is the distance between atoms i and j, ε_{ij} and σ_{ij} are the LJ well-depth and diameter, respectively. For the framework atoms, the interaction parameters were obtained from the DREIDING force field [5]. The TraPPE force fields were used to model C_2H_6, C_2H_4 [6] and N_2 [7] molecules. The LJ parameters for different atom type interactions were approximated with the Lorentz-Berthelot mixing rules (Eq. S3-4):

$$\varepsilon_{ij} = \sqrt{\varepsilon_{ii}\varepsilon_{jj}} \quad \text{(Eq. S3)}$$

$$\sigma_{ij} = \frac{\sigma_{ii}\,\sigma_{jj}}{2} \quad \text{(Eq. S4)}$$

The non-bonded interactions for both adsorbate-adsorbate and adsorbate-framework interactions were truncated at 14.0 Å with the analytic tail correction. Periodic boundary conditions (PBC) were applied to x, y, and z directions to satisfy the minimum image conventions with respect to the 14.0 Å cutoff. The framework atoms forming the MOFs are held fixed at their crystallographic positions during MC and GCMC simulations. All MC and GCMC simulations were carried out using the open-source RASPA 2.0. [8] The potential energy surface of **Ni(IN)$_2$** was calculated with methane molecule as a probe using iRASPA software.[9]

Molecular Dynamics (MD) simulations were carried out to obtain relaxed **Ni(IN)$_2$**. NPT MD simulations were carried out for 1 ns for pristine **Ni(IN)$_2$** at 298 K and 1 atm. Following the NPT MD

simulation, **Ni(IN)$_2$** structure was relaxed under the NVT ensemble at 0 K. The Nose-Hoover thermostat and barostat were used to maintain the system's temperature and pressure damping parameters of 0.1 ps and 1.0 ps, respectively. Velocity-verlet integrator was used to numerically integrate the Newton's equations of motion with 0.1 fs time steps. Conjugated gradient (CG) and fire algorithms, as implemented in the LAMMPS simulation package (ver 12 Dec 2018) were used for the energy minimization step. The energy difference between subsequent cycles becomes less than 1.0 x 10^{-6} kcal/mol. Bonded interactions between framework atoms were approximated based on UFF4MOF force field, as reported by Coupry *et al.*[10] Force field parameters were assigned using the LAMMPS interface python module developed by Boyd *et al.*[11] All MD simulations were carried out using LAMMPS.[12] Material Studio[13] was used for visualization of both pristine **Ni(IN)$_2$** and relaxed **Ni(IN)$_2$**. For the C_2H_6, C_2H_4 and N_2 adsorption isotherms in relaxed **Ni(IN)$_2$**, the GCMC simulations were carried out in the manner mentioned above. For structural comparison between pristine **Ni(IN)$_2$** and relaxed **Ni(IN)$_2$**, the structural features (pore size distribution (PSD), accessible pore volume, and pore limiting diameter (PLD)) of both MOFs were computed using the zeo++ open source software.[14] The pore size distributions (PSD) of both MOFs were computed using a rigid sphere with a radius of 1.655 Å, which is the LJ σ parameter of N_2 in the TraPPE model to obtain the pore size of both MOFs. The accessible pore volumes of both MOFs were calculated with a radius of 0 Å.

Density functional theory (DFT) calculations were carried out using CP2K package[15] The PBE functional[16] with the Grimme D3 correction[17] was used. The Goedecker−Teter−Hutter (GTH) pseudopotentials[18] and DZVP-MOLOPT-GTH basis sets[19] were utilized with energy cutoff of 1000 Ry. The Broyden−Fletcher−Goldfarb−Shanno (BGFS) algorithm was used for geometry optimization. The binding energies between gas molecule and MOF were calculated by

$$E_{B.E.} = E_{MOF+gas} - E_{gas} - E_{MOF} \qquad \text{(Eq. S5)}$$

where $E_{MOF+gas}$ is the energy from the optimized configuration of C_2H_6/C_2H_4 molecule in **Ni(IN)$_2$**, E_{gas} is the energy of the optimized C_2H_6/C_2H_4 molecule and E_{MOF} is the energy of the optimized **Ni(IN)$_2$**.

For the adsorbent evaluation at the process-level, an ideal vacuum swing adsorption (VSA) process is adopted in this work[20], which models the VSA process by excluding the heat and dispersion and void volume effects. The method computes the best possible performance that an adsorbent material can achieve under the ideal circumstances. The ideal VSA simulation only consists of adsorption and desorption steps, and we calculated the C_2H_4 recovery during the adsorption and desorption step to evaluate the performance of adsorbent materials. Here, C_2H_4 is a raffinate component produced during the adsorption step, while the unreacted ethane was recovered during the desorption step. The purity of C_2H_4

is 100% for all materials evaluated in this work since the materials preferentially adsorb C_2H_6 over C_2H_4 during the adsorption step. Depending on the adsorbents, some C_2H_4 is adsorbed during the adsorption step, which is lost during the desorption step. The extent of loss and the recovery of C_2H_4 is closely related to the economic performance of the VSA process. A high C_2H_4 recovery is directly related to a higher production rate of C_2H_4 and a smaller loss of the raw material.

The ideal VSA process produces 100% purity of raffinate, which is ethylene in our case. This high-level of purity can be achieved by judiciously selecting the operating conditions of the process, such as step times, rinse flowrate, which has been experimentally demonstrated by Park *et al.*[21] However, the modification of the operating conditions leads to the trade-offs between the recovery and purity of ethylene product. One of the key materials properties that can be tuned from the operating condition is mass transfer coefficient. Unfortunately, the accurate estimation of the mass transfer coefficients of all adsorbents are computationally and experimentally difficult and time consuming. Ideal VSA simulation allows us to remove the need to use the mass transfer coefficient to evaluate the performance of adsorbent materials at the process-level.

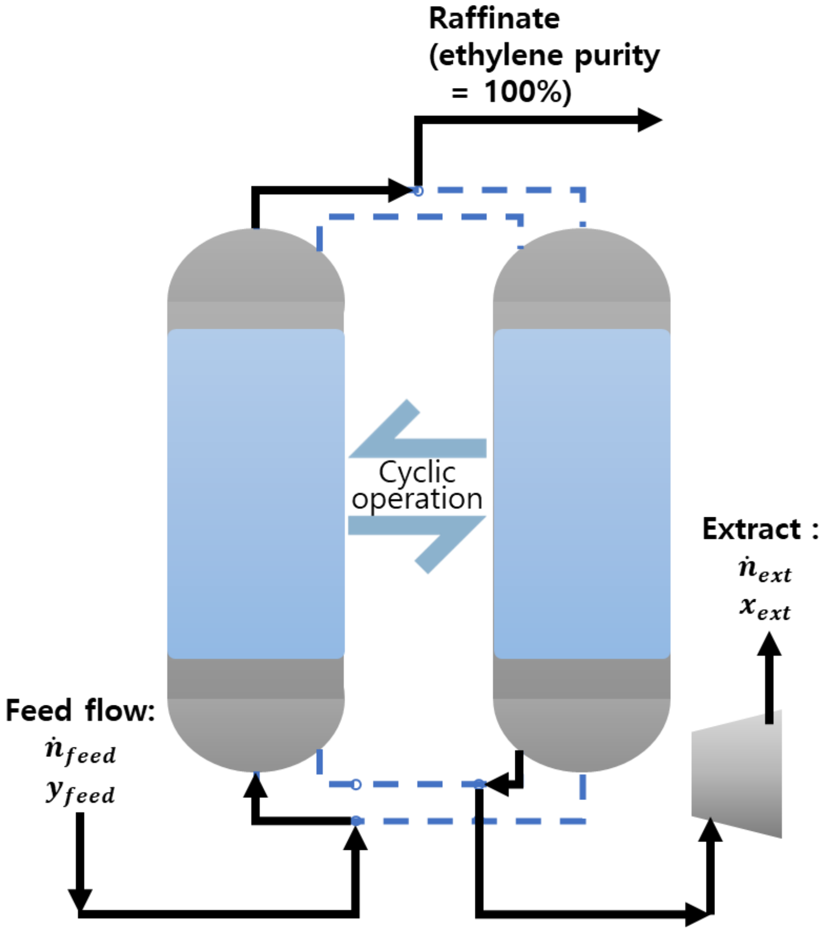

Figure S1. VSA process schematic and its key parameters.

We modified the original method that has been developed for the CO_2/N_2 separation for the case of C_2H_4/C_2H_6 separation. The key parameters are marked in Figure S1. Based on the assumption that the process produces 100% ethylene through the raffinate flow, we can write down the mass conservation of ethylene as in Eq. S6:

$$(1 - y_{feed})\dot{n}_{feed} = (1 - x_{ext})\dot{n}_{ext} \quad \text{(Eq. S6)}$$

Here, y_{feed} is the mole fraction of ethylene in the feed flow, x_{ext} is the mole fraction of ethylene in the extract, $\dot{n}_{feed}$ and $\dot{n}_{ext}$ are the total molar flow rate of feed and extract in the unit of mol per time.

The ethylene purity in the raffinate, Rec_{C2H4}, is formally defined as:

$$Rec_{C2H4} = \frac{(In)_{C_2H_4} - (Loss)_{C_2H_4}}{(In)_{C_2H_4}} = \frac{y_{feed}\dot{n}_{feed} - x_{ext}\dot{n}_{ext}}{y_{feed}\dot{n}_{ext}} \quad \text{(Eq. S7)}$$

By combining the Equations S5 and S6, we can obtain the equation for the raffinate recovery as below:

$$Rec_{C2H4} = 1 - \frac{x_{ext}}{(1 - x_{ext})}\frac{(1 - y_{feed})}{y_{feed}} \quad \text{(Eq. S8)}$$

Here, Rec_{C2H4} is the recovery of ethylene in the raffinate, x_{ext} and y_{feed} are the molar fraction of C_2H_4 in the extract and feed flows, respectively. For the application of the adsorbent properties, the ideal adsorbed solution theory (IAST) was employed with the quadratic, Langmuir or dual-site Langmuir isotherm parameters explaining the pure adsorption isotherm (see the Supplementary Information S7). Each molecular simulation result or the experiment dataset was fitted with the most suitable model among the isotherm models supported in pyIAST.[22] The mixture isotherms were predicted using the pure component isotherms as input. Details of fitting isotherm parameters are provided as Section S7 of the Supplementary Information (SI).

Isosteric heat of adsorption calculations: The coverage-dependent adsorption enthalpy profiles were calculated from the sorption data measured at 273, 298, and 323 K by Virial fitting method and Clausius-Claperyron equation. A Virial-type expression was used (Eq. S9), which is composed of parameters ai and bi, which are independent of temperature. In Eq. S9, P is the pressure in atm, N is the adsorbed amount in mmol g^{-1}, T is the temperature in Kelvin, a_i and b_i are the Virial coefficients, and m and n represent the number of coefficients required to adequately describe the isotherms.

$$ln\, p = ln\, N + \frac{1}{T}\ \sum_{i=0}^{l} a_i n^i + \sum_{j=0}^{m} b_j n^j \quad \text{(Eq. S9)}$$

To calculate Q_{st}, the fitting parameters from the Eq. S9 were used for the Eq. S10.

$$Q_{st} = -R \left[\frac{\partial ln ln\, p}{\partial\left(\frac{1}{T}\right)} \right]_n = -R \sum_{i=0}^{l} a_i n^i \quad \text{(Eq. S10)}$$

Derivation of the propagation rate of saturated adsorbent area

From the process engineering point of view, the main advantage of C_2H_6 selective material is that the product (ethylene) exits first during the adsorption step. To see if this is true for all the materials considered in this work, we derived the propagation rates of the saturation region for both ethane and ethylene, and compared if the ethane is always small. The propagation rate is calculated in the following way:

$$r_i = \frac{l_{sat}A}{t_{unit}} \quad \text{(Eq. S11)}$$

$$\varepsilon \Delta q_i \rho_s l_{sat} A = y_i F t_{unit} \quad \text{(Eq. S12)}$$

$$r_i^o = \frac{\varepsilon\rho}{F} r_i = \frac{y_i}{\Delta q_i} \quad \text{(Eq. S13)}$$

where, r_i is a propagation rate of the saturated adsorbent area for component *i* during the adsorption time. For different gas component, they have different propagation rate. Because the area propagates in an axial way, the rate can be written in Eq. S11, where l_{sat} and *A* are axial length of the saturated adsorbent area and cross-sectional area, respectively. The propagation rate is how much of the area expanded for a unit period time, t_{unit}. From the mass balance of the entire adsorption column system, during the adsorption step, the amount of adsorbed component *i* is equivalent to the injected amount of component *i*. This correlation is expressed in Eq.S12. On the left-hand side, to express the adsorbed amount, $\varepsilon, \Delta q_i$, and ρ_s are used standing for void fraction, uptake change between the adsorption and desorption period, and the solid density of the adsorbent. For the injected component *i*, on the right-hand side, y_i and F are additionally used as feed composition and overall feed flowrate. By substituting $l_{sat}A$ with the correlation of S11, the propagation rate can be derived. Since the two components share the void fraction, adsorbent density, and overall feed flowrate in the same system, we can reduce the equation in the Eq. S13. Thus, by comparing the reduced propagation rate, r_i^o, we can see which component would be a raffinate (leading) component in the VSA process. In all the MOF cases tested in this screening work, the reduced propagation rate of ethylene ($r_{C_2H_4}^o$) is faster than the rate of ethane ($r_{C_2H_4}$), which indicates that we can

take full advantage of the C_2H_6 selective adsorbents in all cases. All data and analyses used in this work can be accessed from: https://github.com/yoonseonghyun/2020-C2.git

S2. Analytical characterizations

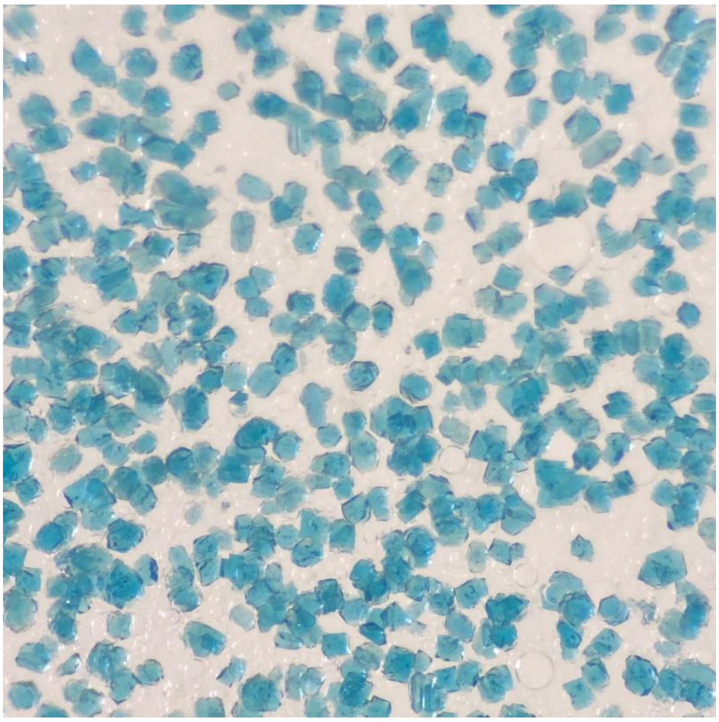

Figure S2. An optical micrograph of **Ni(IN)$_2$**.

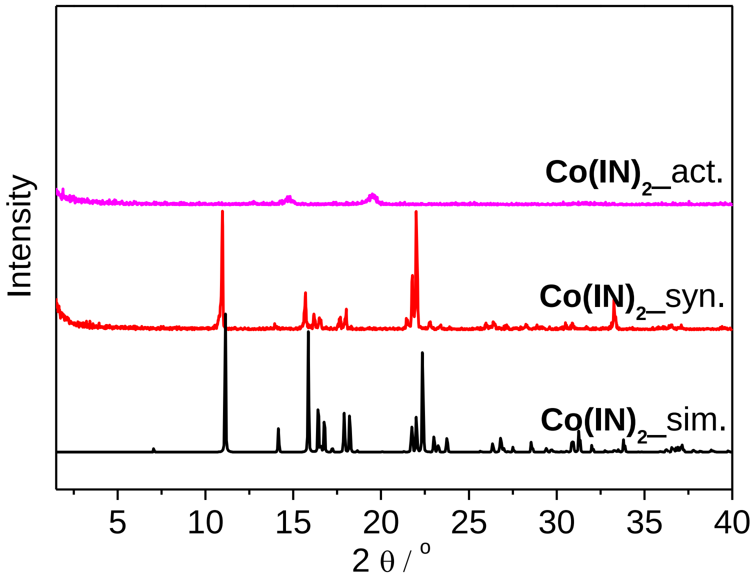

Figure S3. Powder X-ray diffraction profiles of simulated, as synthesized, and activated **Co(IN)$_2$**.

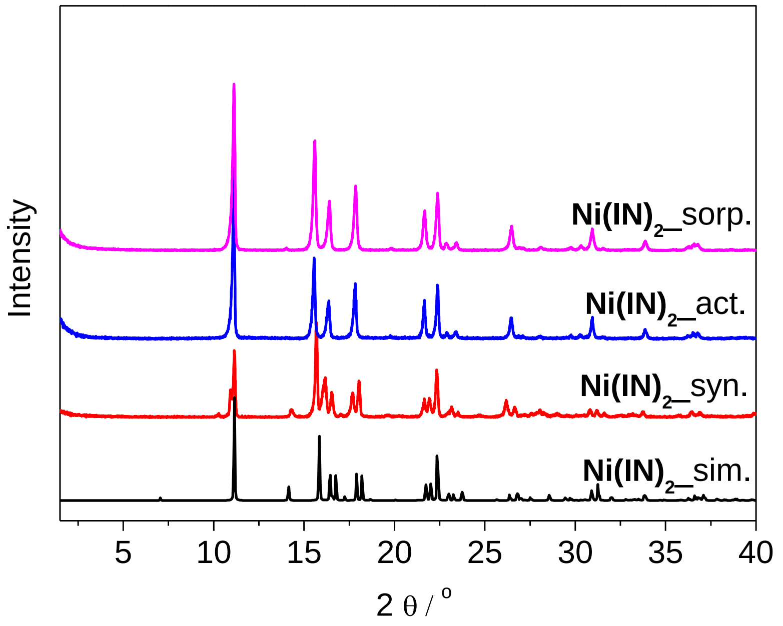

Figure S4. Powder X-ray diffraction profiles of **Ni(IN)₂** simulated, as synthesized, activated, and after gas sorption measurements.

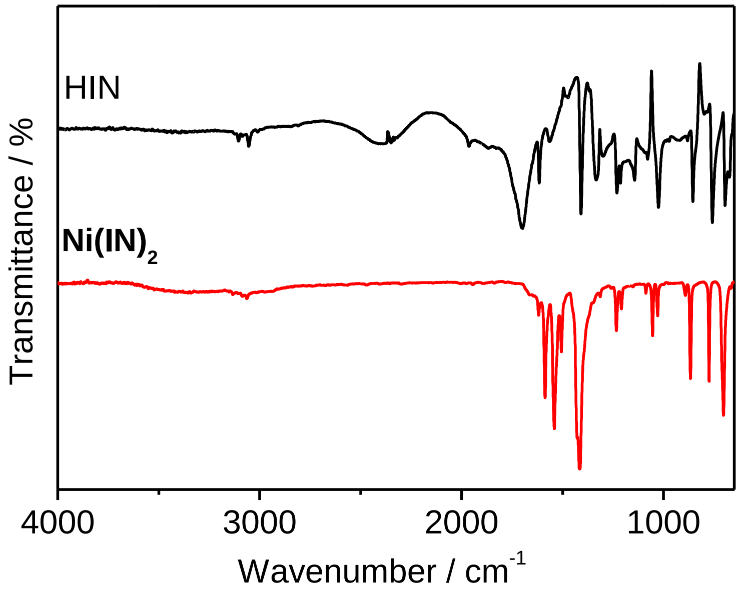

Figure S5. IR spectra of HIN and **Ni(IN)₂**.

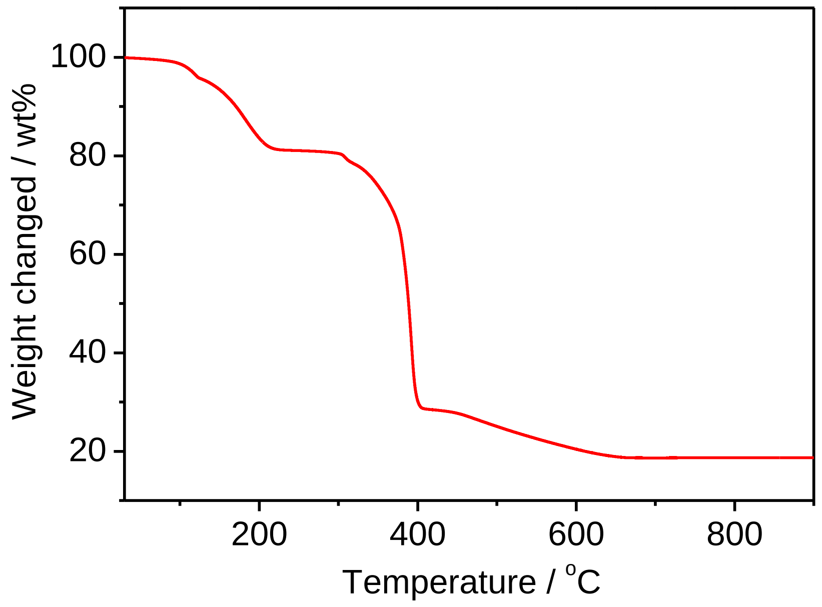

Figure S6. TGA curve for **Ni(IN)$_2$.**

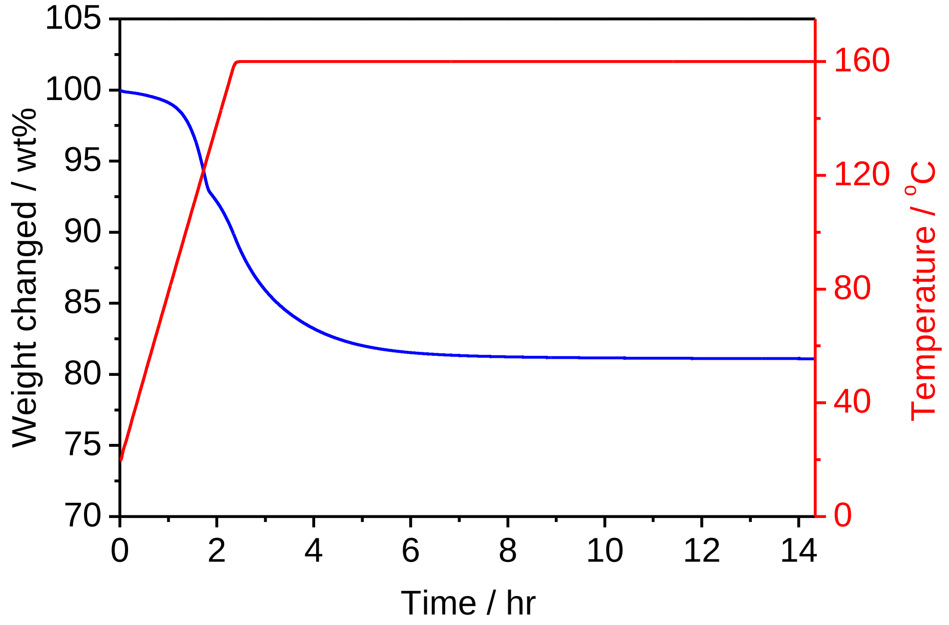

Figure S7. TGA curve for **Ni(IN)$_2$** under the activation condition of the breakthrough experiment.

S3. Adsorption studies

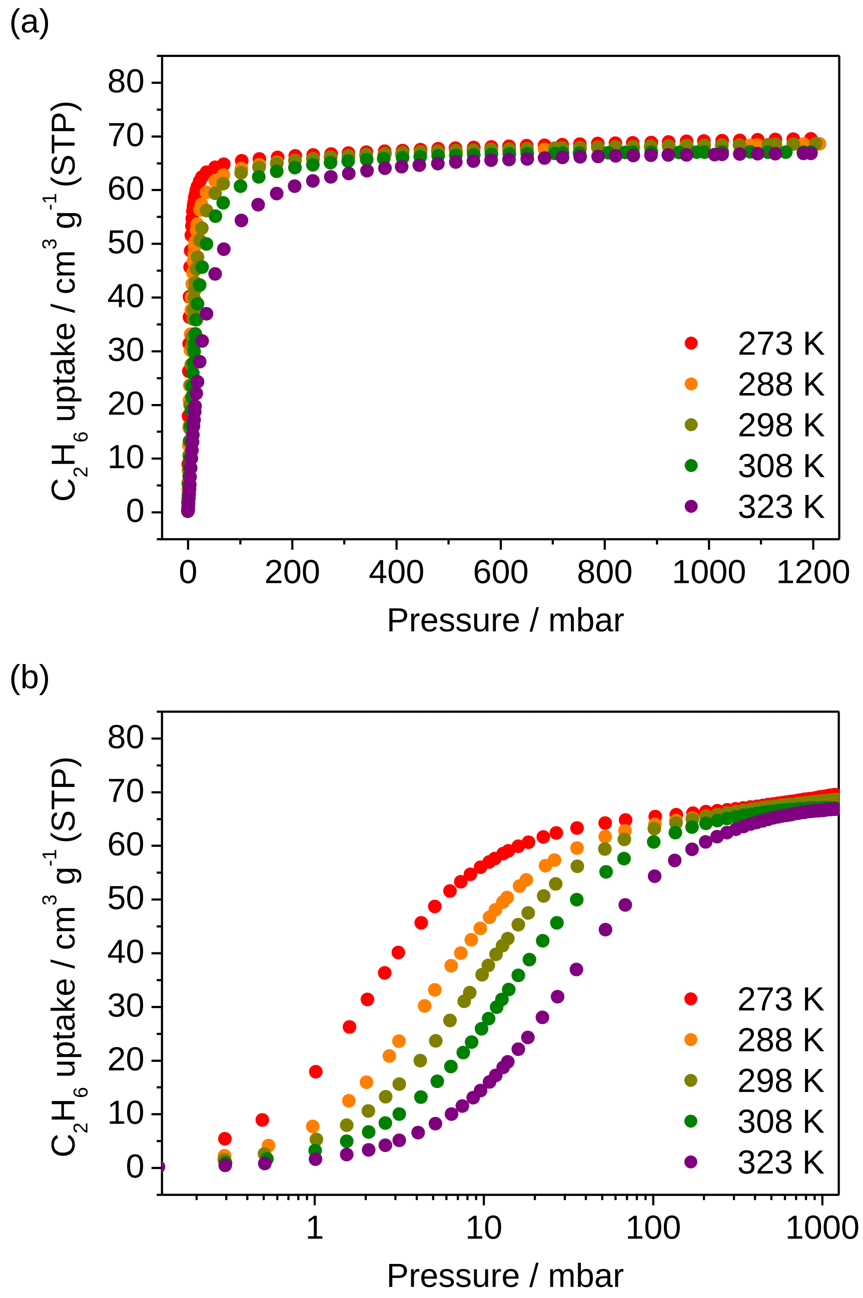

Figure S8. C_2H_6 sorption isotherms of **Ni(IN)$_2$** on a linear (a) and log (b) scale at 273, 288, 298, 308, and 323 K.

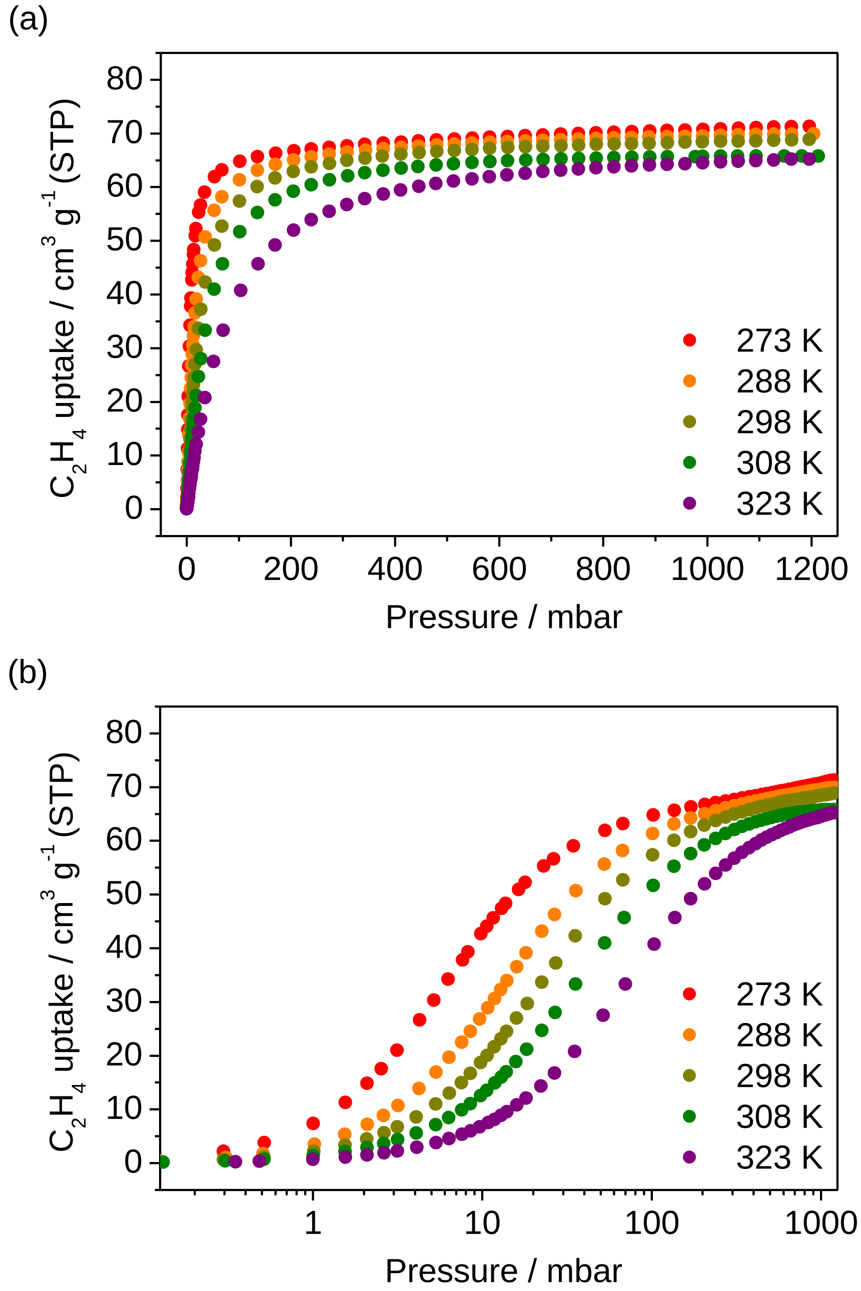

Figure S9. C_2H_4 sorption isotherms of **Ni(IN)$_2$** on a linear (a) and log (b) scale at 273, 288, 298, 308, and 323 K.

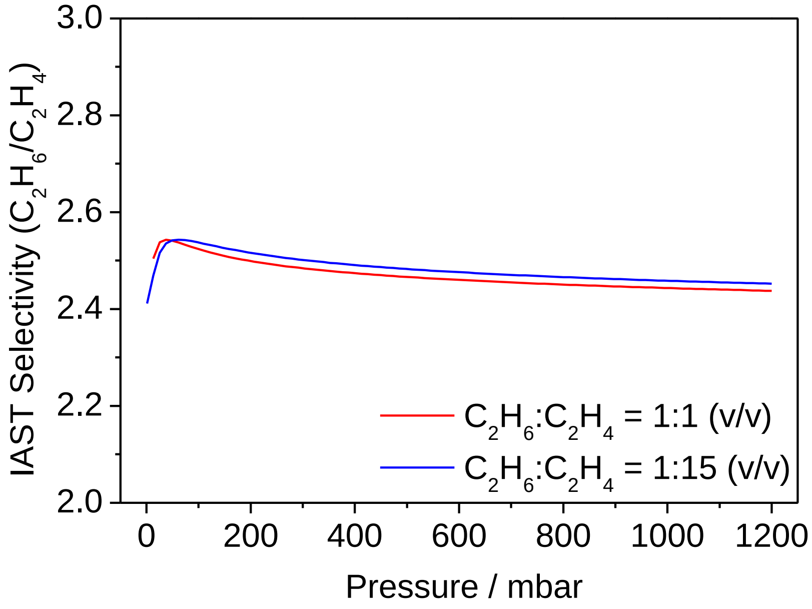

Figure S10. Selectivity of **Ni(IN)$_2$** predicted by the IAST method for 1:1 and 1:15 C_2H_6/C_2H_4 (v/v) mixture at 298 K, respectively.

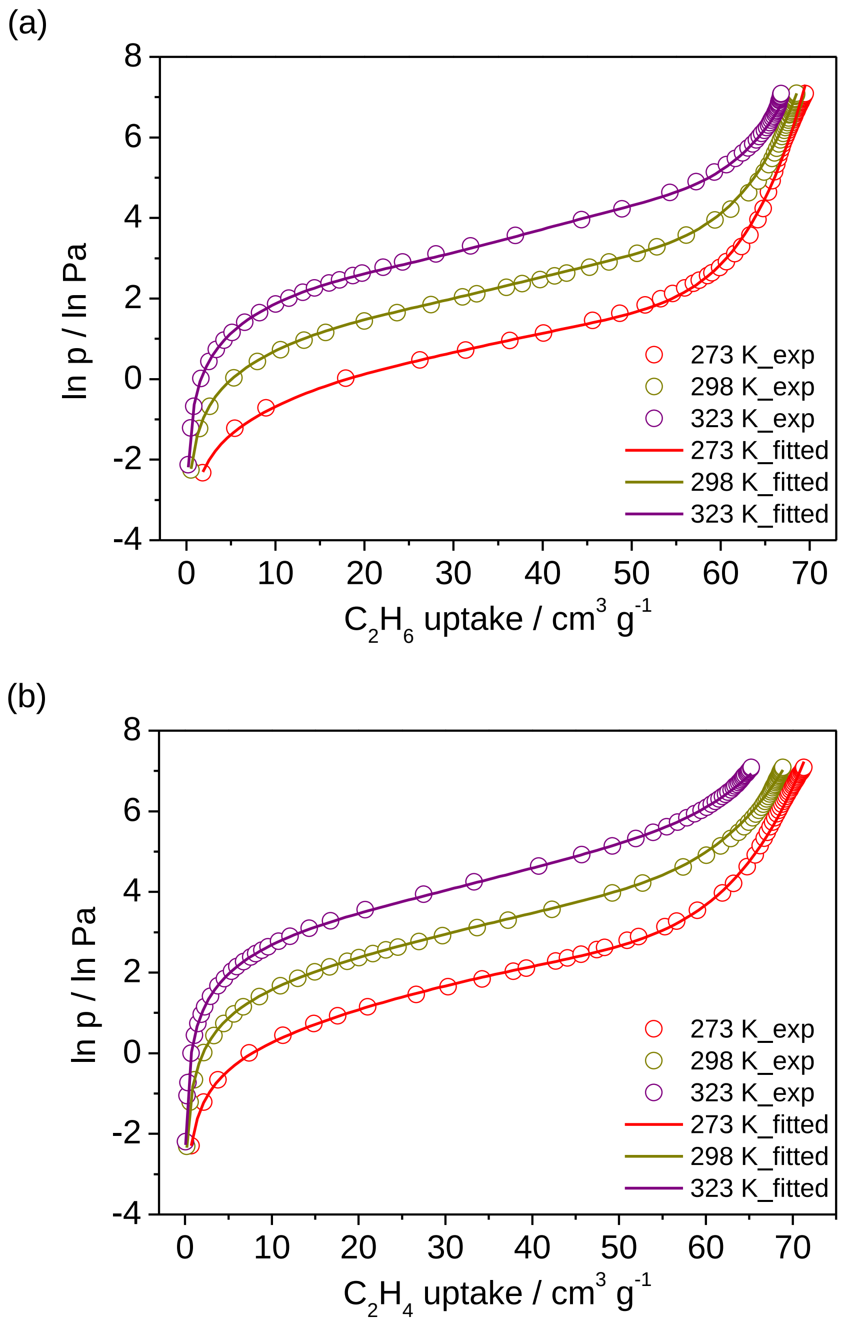

Figure S11. Comparison of experimental isotherms to the ones obtained from Virial modeling carried out using C_2H_6 (a) and C_2H_4 (b) isotherms collected at 273, 298, and 323 K.

Table S1. Summary of the fitted Virial parameters.

	C_2H_6	C_2H_4
$\mathbf{a_0}$	-4141.88618	-4002.05056
$\mathbf{a_1}$	-94.39141	-64.2689
$\mathbf{a_2}$	8.37645	4.93421
$\mathbf{a_3}$	-0.30421	-0.14228
$\mathbf{a_4}$	0.00556	0.00174
$\mathbf{a_5}$	-6.12822×10^{-5}	-1.3108×10^{-5}
$\mathbf{a_6}$	3.62302×10^{-7}	9.02428×10^{-8}
$\mathbf{b_0}$	12.36173	12.72599
$\mathbf{b_1}$	0.2759	0.20095
$\mathbf{b_2}$	-0.02168	-0.01472
$\mathbf{b_3}$	6.08624×10^{-4}	3.9565×10^{-4}
$\mathbf{b_4}$	-5.28997×10^{-6}	-3.2429×10^{-6}

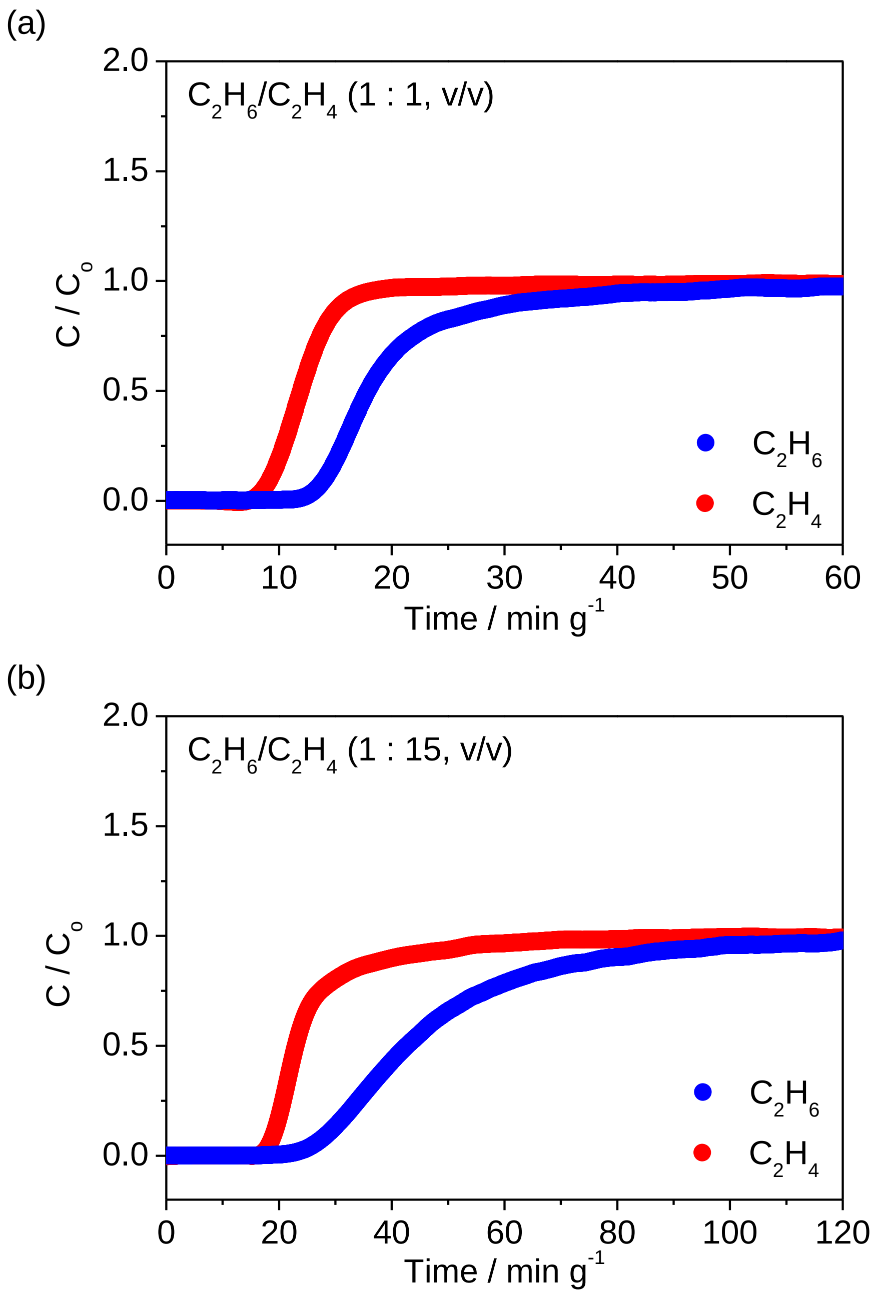

Figure S12. Breakthrough curves for a mixture of C_2H_6/C_2H_4 in volumetric ratios of (a) 1:1 and (b) 1:15 at 298 K and 1 bar.

S4. Comparison of experimental and simulated results

To obtain relaxed **Ni(IN)$_2$** structure, MD simulations were carried out for pristine **Ni(IN)$_2$** structure. As shown in **Figure S12** and **Table S2**, the relaxed **Ni(IN)$_2$** has relatively larger pore size and pore volume than the pristine **Ni(IN)$_2$**. Both MOFs were used to compare experimental and simulated isotherms. **Figure S13** shows that the N_2 isotherm of the relaxed **Ni(IN)$_2$** is similar to experimental isotherm compared with data of pristine **Ni(IN)$_2$**. Additionally, as shown in **Figure S14** and **Table S4**, the single component C_2H_6 and C_2H_4 adsorption isotherms of the relaxed one are more close to experimental data compared with pristine one. The 1 bar selectivity of the relaxed **Ni(IN)$_2$** was 2.91 that is closer to the selectivity value (2.45) obtained based on the experimental data, suggesting that the relaxed structure is more representative of the structure under experimental condition. However, it is not possible to obtain relaxed structures by applying MD simulation to all MOFs of CoRE MOF 2019 database due to large computational costs. Therefore, we considered MD simulation for only **Ni(IN)$_2$**, which is the best performing MOF emerging from the high-throughput computational screening.

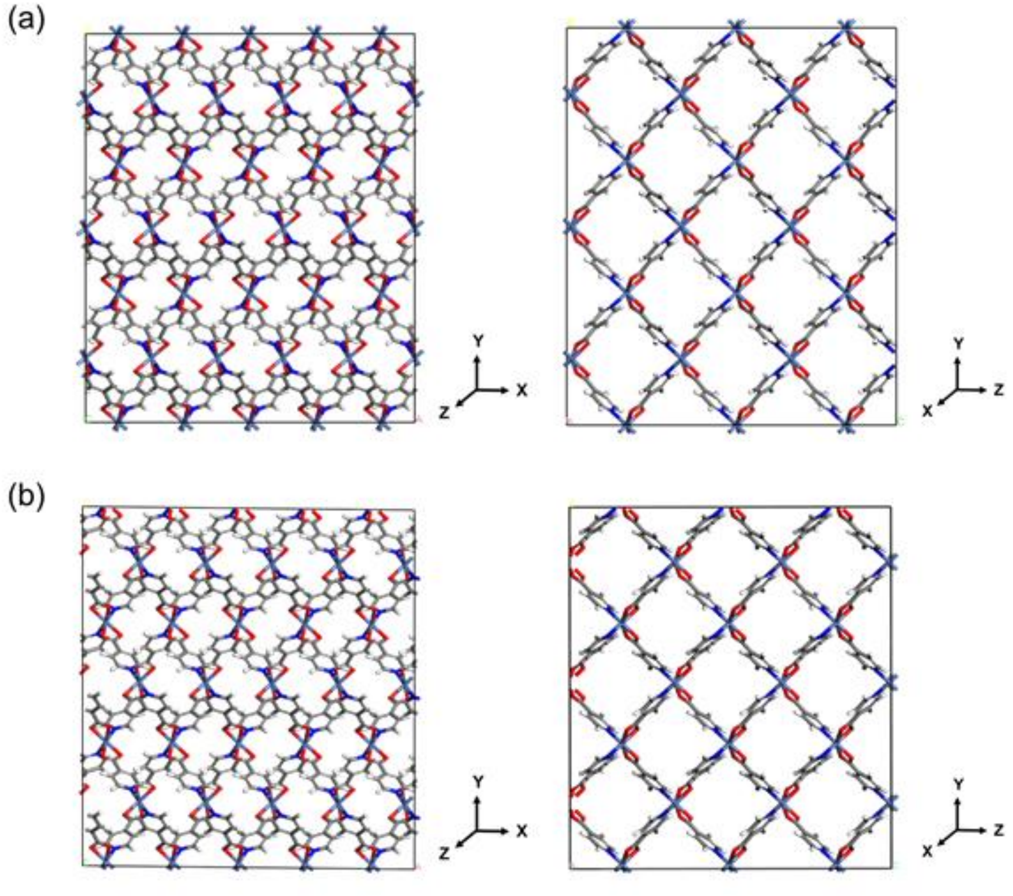

Figure S13. Atomistic model of (a) pristine **Ni(IN)$_2$** and (b) relaxed **Ni(IN)$_2$** obtained from NPT/NVT MD simulation. The figure on the left is a z-direction view and the figure on the right is a x-direction view.

Table S2. Comparison of structure properties and cell parameters for pristine and relaxed **Ni(IN)$_2$**.

	pristine **Ni(IN)$_2$**	relaxed **Ni(IN)$_2$**
cell length a (Å)	31.78	33.77
cell length b (Å)	37.49	36.49
cell length c (Å)	31.14	32.43
cell angle α (°)	90.00	90.02
cell angle β (°)	90.00	89.40
cell angle γ (°)	90.00	90.63
PLD (Å)	4.10	4.26
pore volume (cm^3 g^{-1})	0.42	0.46
void fraction (-)	0.51	0.52
main pore size (Å)	5.3	5.4

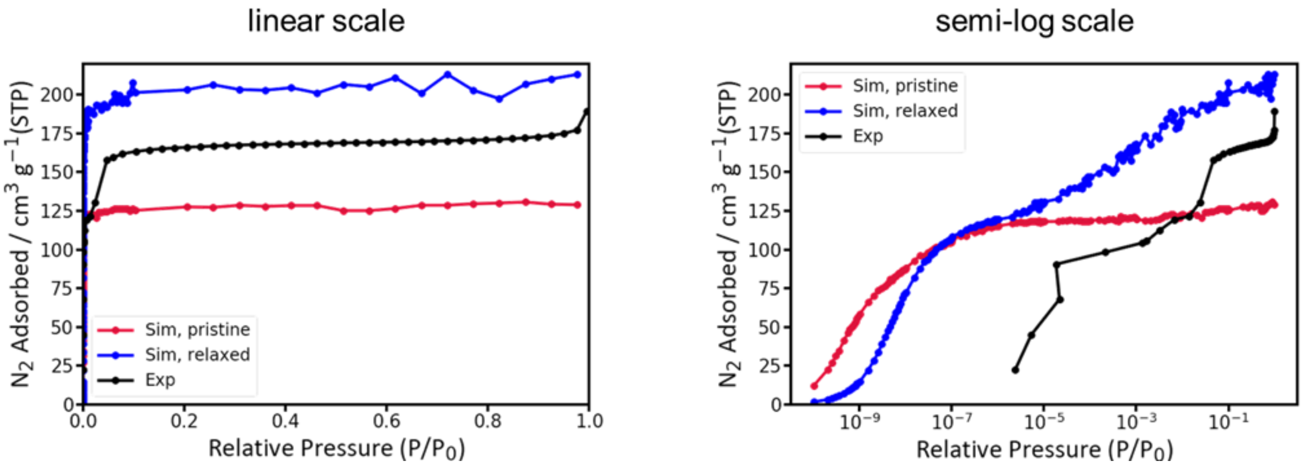

Figure S14. Simulated and experimental N_2 adsorption isotherms of **Ni(IN)₂** at 77 K.

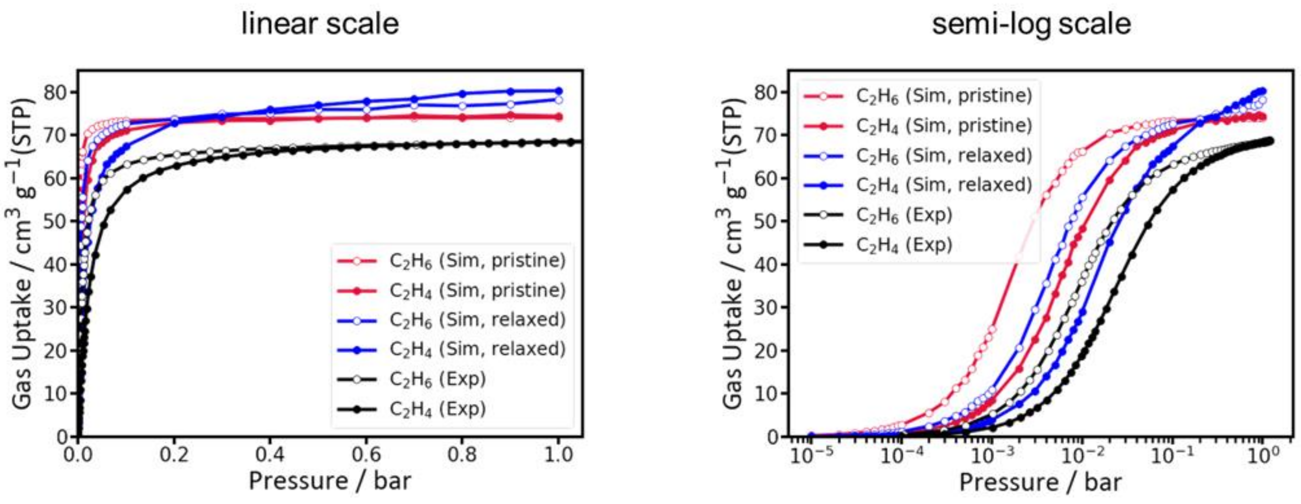

Figure S15. C_2H_6 and C_2H_4 adsorption isotherms of **Ni(IN)$_2$** at 298 K obtained from simulation and experiment.

Table S3. Comparison of 1 bar selectivity (C_2H_6/C_2H_4) from simulation and experiment in **Ni(IN)$_2$**.

Experiment[a] (IAST)	Simulation[b]	
	pristine	relaxed
2.44	3.65	2.91

[a]Experimental selectivity was calculated from IAST method.

[b]Simulated selectivities were calculated from binary component GCMC results.

S5. Ideal VSA simulations results with different operating conditions

Additional process-level analyses with different vacuum pressure (i.e., desorption pressure) were performed to elucidate the parameter's sensitivity on the performance. The results are shown in **Figure S16 – S18**. For varying vacuum pressure, we tested the top 10 candidates selected in the screening work with the CoRE MOF 2019 database. **Figure S16** shows the VSA process performances of the top 10 candidates. This evauation was performed for the varying vacuum pressure from 0.01 mbar to 10 mbar. The ethylene recoveries of the VSA processes with each adsorbent are displayed as different vaccum pressures are used during the desorption step. In **Figure S17**, the adsorbents from the literature are compared with **Ni(IN)$_2$** for the varying vacuum pressure with the same setting of **Figure S16**. **Figure S18** has the results of ideal VSA process where 16.44% of ethane is used as a feed flow, which has been reported by Park *et al*.[21] In these three cases, as the vacuum pressure decreases, the recovery values of all the MOFs become improved. This trend can be explained with the correlation where the energy input and the extent of separation in this process. Stronger vacuum needs increased energy for the process operation, but the process can harness more energy to separate the target gas. Thus, more energy input to the process leads to lower vacuum pressure, which makes better separation of C_2H_6/C_2H_4. Moreover, depending on the adsorption isotherm properties, the slope of the recovery is shown to be different. While MAF-49 and **Ni(IN)$_2$** shift their performances significantly as the vacuum pressure reduces, the other MOFs have relatively small changes. These different performance changes indicate that different operating conditions of the process should be considered during the adsorbent selection work for the adsorptive process design.

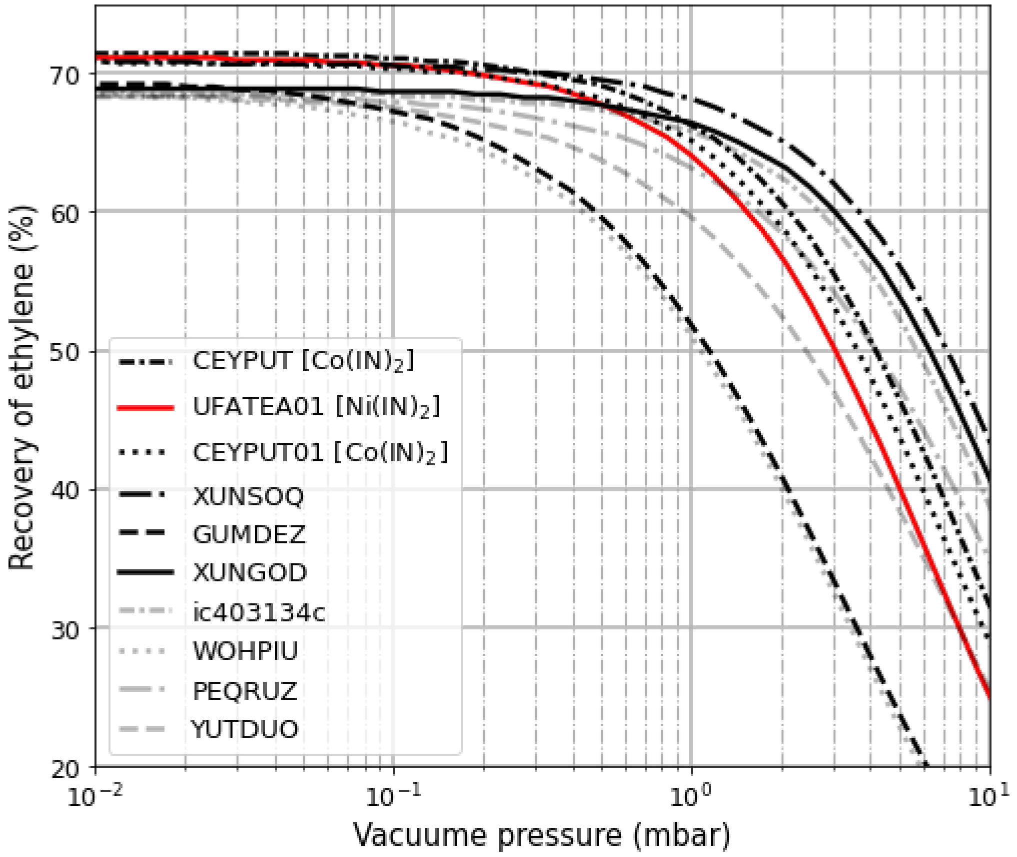

Figure S16. Adsorbent evaluation (ethylene recovery) with ideal VSA simulations for feed of C_2H_6/C_2H_4 in volumetric ratio of 1:15 at 298K and 1 bar. The ideal VSA simulations are based on molecular simulation results of the top 10 adsorbents found from the screening of CoRE MOF.

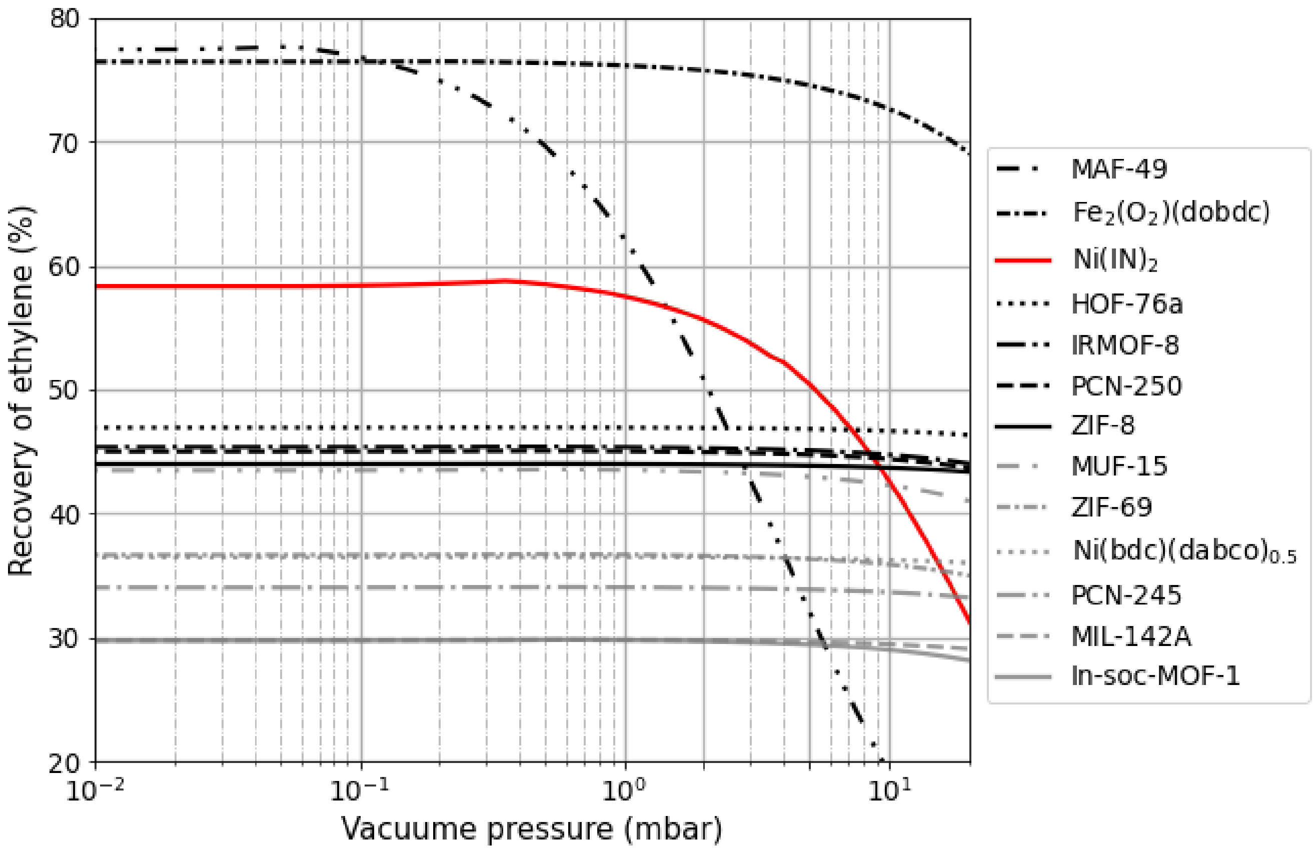

Figure S17. **$Ni(IN)_2$** Performance compared with other adsorbents recently reported as high performing adsorbents for C_2H_6/C_2H_4 separation.

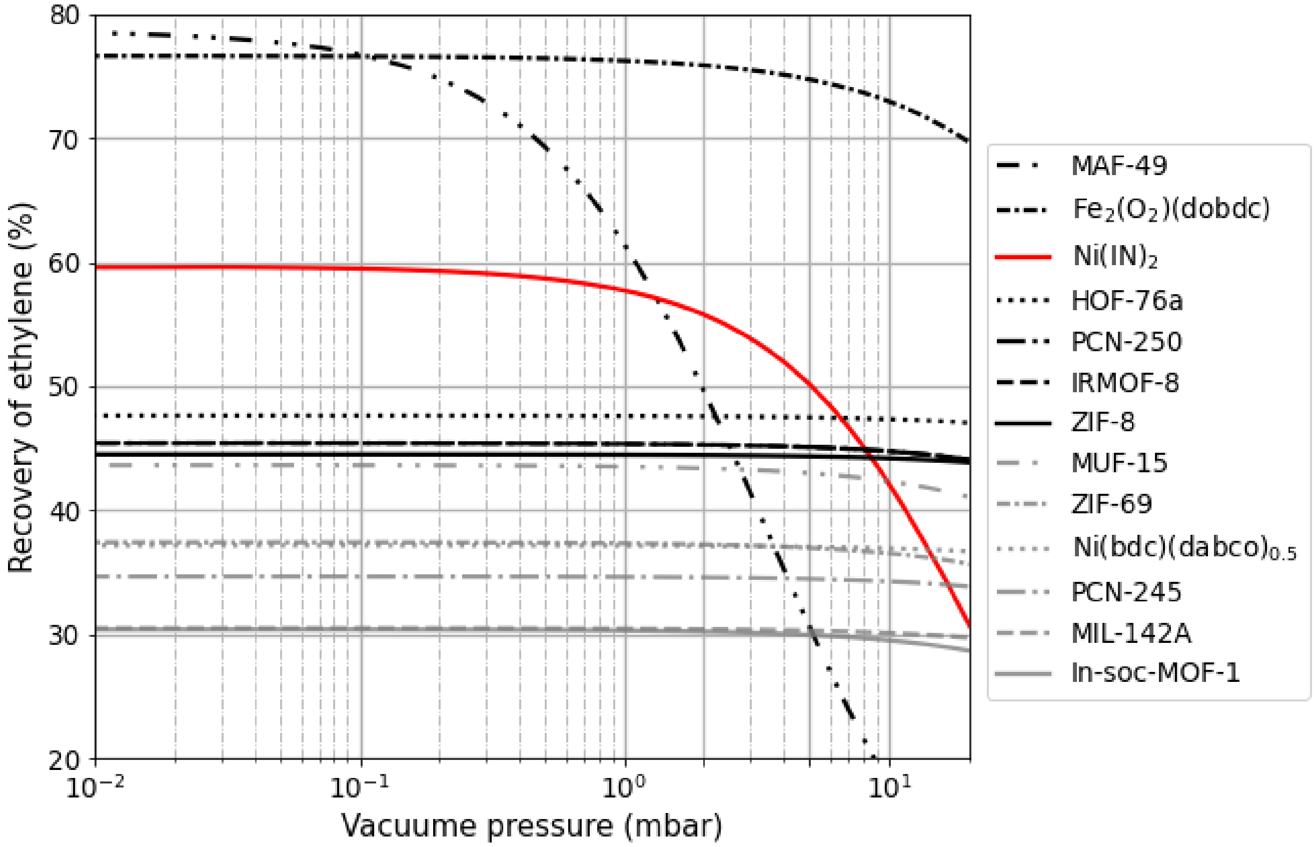

Figure S18. Case of 16.44% ethane feed for ideal VSA process employing **$Ni(IN)_2$** and other adsorbents recently reported in the literature.

Table S4. Evaluation of top 10 candidates in CoRE MOF 2019 database (% ethylene recovery) with ideal VSA simulations for the feed of C_2H_6/C_2H_4 in volumetric ratio of 1:15 at 298K and 1 bar (digitized data of **Figure S16**).

	Ethylene recovery (%)		
Name	at P_{des} = 0.01 mbar	at P_{des} = 10 mbar	at P_{des} = 50 mbar
CEYPUT [**Co(IN)$_2$**]	71.42020933	29.74771755	6.466935032
UFATEA01[**Ni(IN)$_2$**]	71.11117376	25.19565449	4.656526136
CEYPUT01 [**Co(IN)$_2$**]	70.88452070	27.16634314	5.988040873
XUNSOQ	70.74574702	43.66513484	12.57351770
GUMDEZ	69.29404240	12.53777115	0.723818775
XUNGOD	68.93370613	40.95594945	11.31595070
ic403134c	68.61845574	38.94292134	10.34784183
WOHPIU	68.44832929	11.78485813	0.213007665
PEQRUZ	68.42611156	35.11677765	9.124786979
YUTDUO	68.34872518	25.78334660	4.230440690

Table S5. Evaluation of the adsorbents reported in the literature (ethylene % recovery) with ideal VSA simulations for the feed of C_2H_6/C_2H_4 in volumetric ratio of 1:15 at 298K and 1 bar (digitized data of **Figure S17**).

from the literature	Ethylene recovery (%)		
Name	at P_{des} = 0.01 mbar	at P_{des} = 10 mbar	at P_{des} = 50 mbar
MAF-49	77.417363	19.072905	2.8604369
$Fe_2(O_2)$(dobdc)	76.424576	72.618829	60.203557
**Ni(IN)$_2$**	58.323617	42.638544	15.716348
HOF-76a	46.921405	46.644266	45.281778
IRMOF-8	45.360412	44.725336	41.99165
PCN-250	44.998426	44.390884	41.46467
ZIF-8	43.994564	43.695855	42.291293
MUF-15	43.473175	42.293516	37.43611
ZIF-69	36.670786	35.886023	32.514705
Ni(bdc)(dabco)$_{0.5}$	36.465539	36.260584	35.214617
PCN-245	34.025082	33.656455	31.957696
MIL-142A	29.791639	29.471848	27.942547
In-soc-MOF-1	29.746176	29.010324	25.781445

Table S6. Evaluation of the adsorbents reported in the literature (ethylene % recovery) with ideal VSA simulations for feed of 16.44% ethane at 298K and 1 bar (digitized data of **Figure S18**).

from the literature	Ethylene recovery (%)		
Name	at P_{des} = 0.01 mbar	at P_{des} = 10 mbar	at P_{des} = 50 mbar
MAF-49	78.455489	17.875845	2.4536592
$Fe_2(O_2)$(dobdc)	76.60281	72.941795	61.481065
**$Ni(IN)_2$**	59.570697	42.135852	15.156389
HOF-76a	47.63098	47.347465	46.076733
IRMOF-8	45.412523	44.752643	41.871469
PCN-250	45.401798	44.738573	42.110582
ZIF-8	44.499501	44.191551	42.884287
MUF-15	43.640302	42.333643	37.547469
ZIF-69	37.415336	36.540243	33.216874
Ni(bdc)$(dabco)_{0.5}$	37.169699	36.941471	35.934616
PCN-245	34.679341	34.270262	32.625256
MIL-142A	30.484823	30.113567	28.621539
In-soc-MOF-1	30.394131	29.533196	26.309715

S6. Correlation of process-level and molecular-level metrics

To see the correlation of process-level and molecular-level metrics, we computed the Spearman's ranking correlation coefficients (SRCC) which is a nonparametric measure of rank correlation using Eq. S14:

$$r_s = \frac{cov(rg_X, rg_Y)}{\sigma_{rg_X}\sigma_{rg_Y}} \quad \text{(Eq. S14)}$$

where rg_{X_i} and rg_{Y_i} are ranks of raw scores X_i and Y_i, $cov(rg_X, rg_Y)$ is the covariance of the rank variables, σ_{rg_X} and σ_{rg_Y} are the standard derivations of the rank variables. We calculated SRCC for recovery, 1 bar selectivity (50:50) and 1 bar ethane uptake obtained from experimental and simulated isotherm data used in this paper. As shown in **Figure S19**, SRCC between recovery and selectivity is high, while SRCC between recovery and uptake is low. This data supports our approach to select the best performing MOFs based on selectivity.

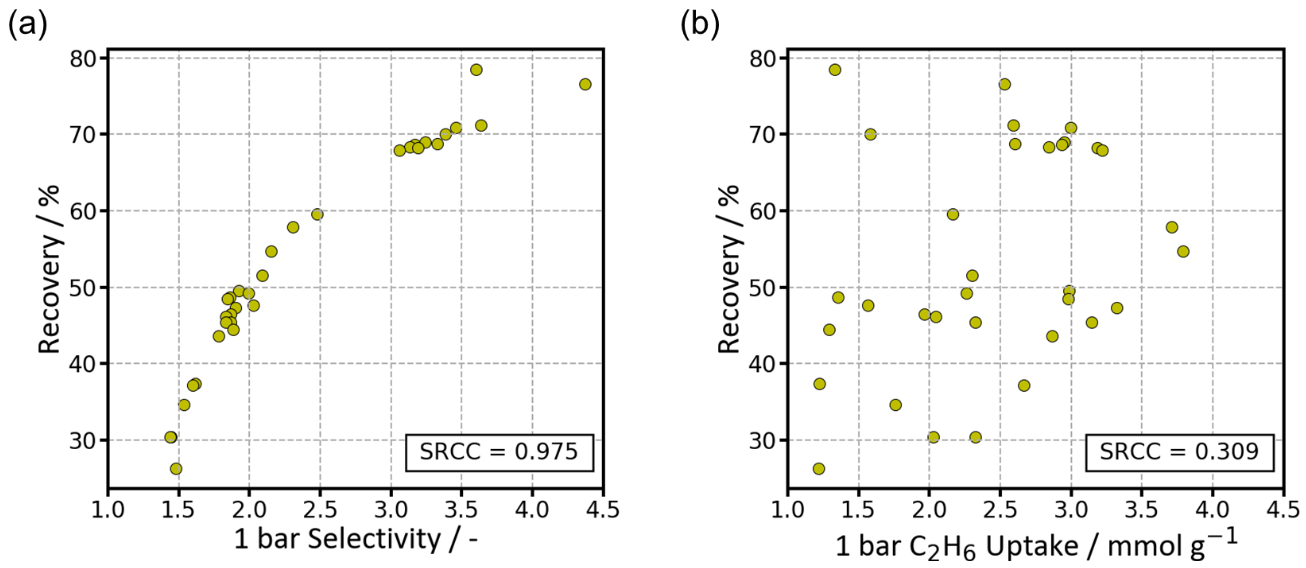

Figure S19. SRCC and comparison of recovery-selectivity distribution in (a) and of recovery-uptake distribution in (b).

S7. Parameter estimations for isotherm models

Molecular simulation results and experimental dataset were fitted with the Quadratic (Eq. S15), Langmuir (Eq. S16) or dual-site Langmuir (Eq. S17) isotherm model.

$$Q(P) = M\frac{(K_a + 2K_b P)P}{1 + K_a P + K_b P^2} \quad \text{(Eq. S15)}$$

$$Q(P) = M\frac{KP}{1 + KP} \quad \text{(Eq. S16)}$$

$$Q(P) = M_1\frac{K_1 P}{1 + K_1 P} + M_2\frac{K_2 P}{1 + K_2 P} \quad \text{(Eq. S17)}$$

where Q is the gas uptake in mmol g^{-1}, P is pressure in bar.

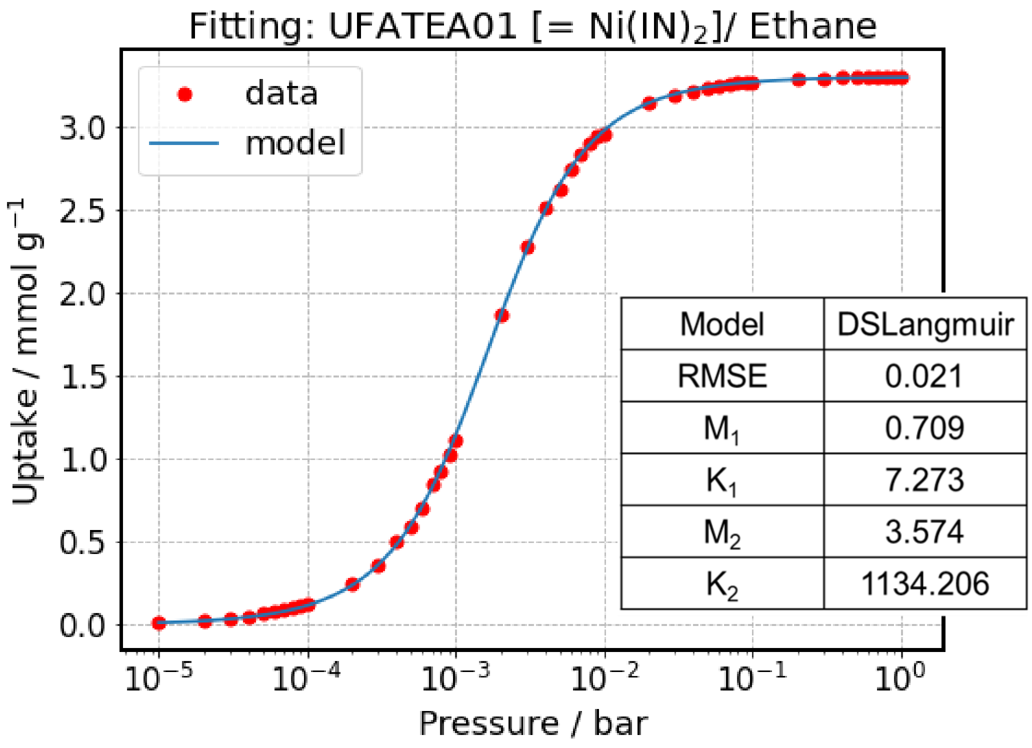

Figure S20. Model fit for simulated ethane adsorption isotherm of UFATEA01[= **Ni(IN)$_2$**].

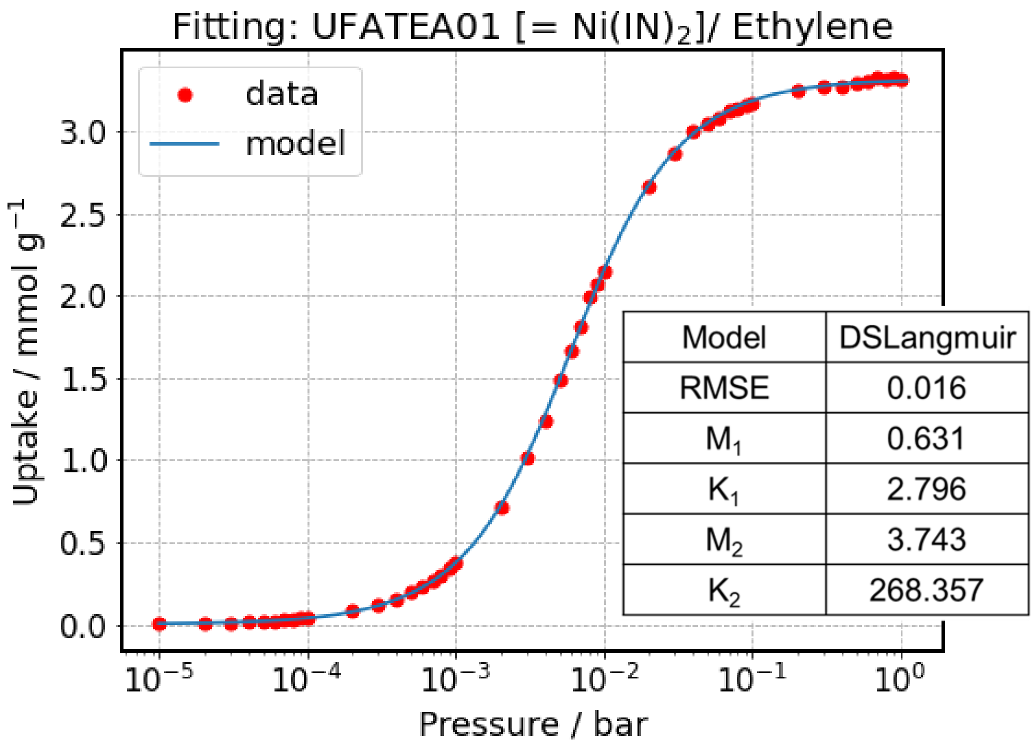

Figure S21. Model fit for simulated ethylene adsorption isotherm of UFATEA01[= **Ni(IN)$_2$**].

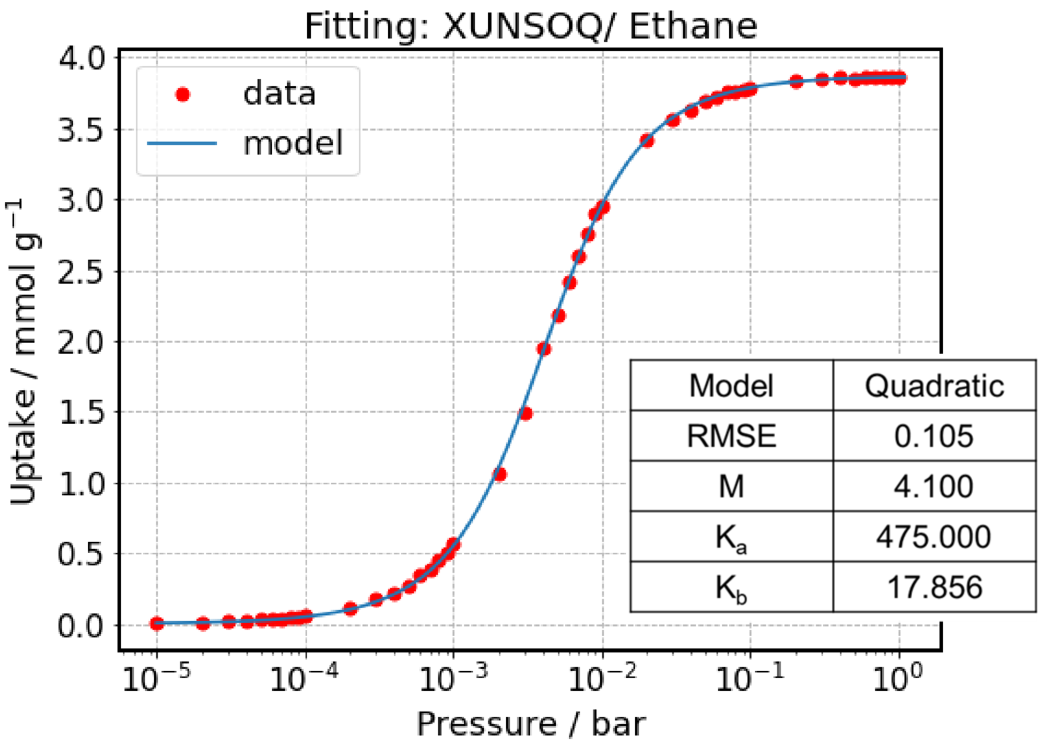

Figure S22. Model fit for simulated ethane adsorption isotherm of XUNSOQ.

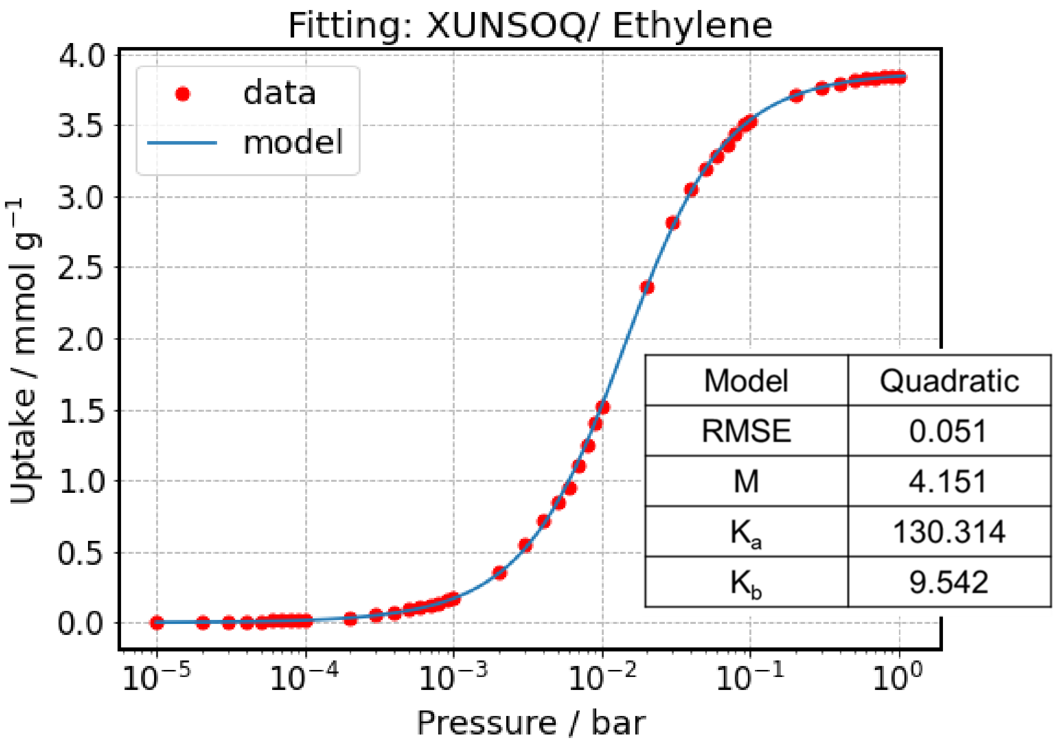

Figure S23. Model fit for simulated ethylene adsorption isotherm of XUNSOQ.

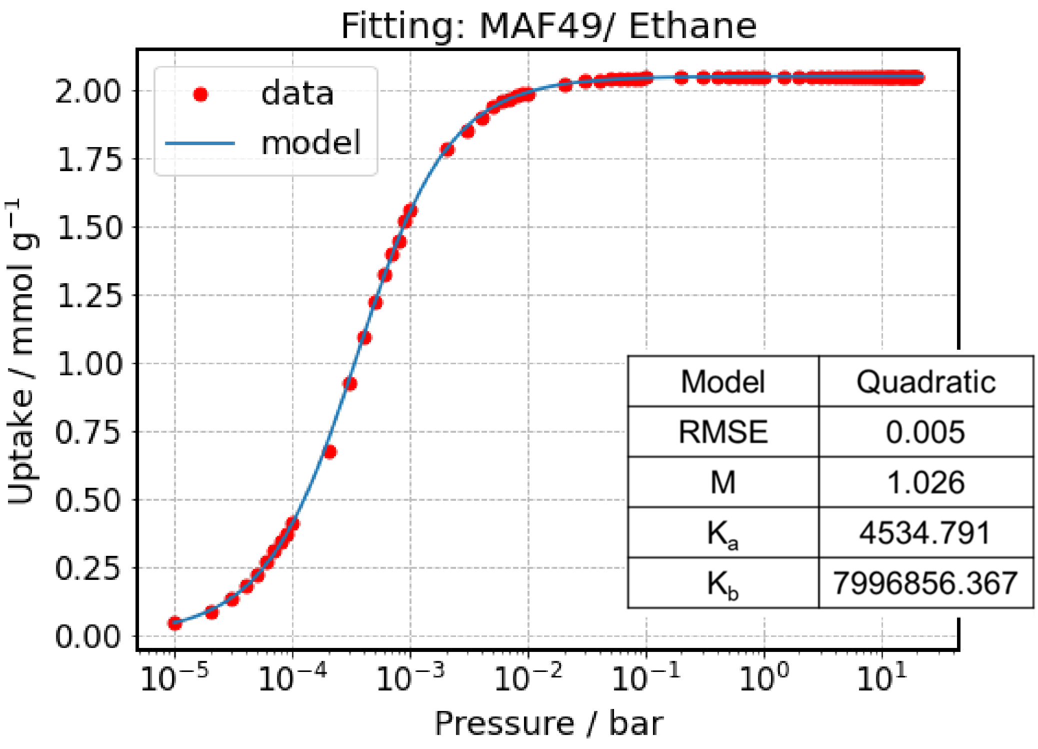

Figure S24. Model fit for simulated ethane adsorption isotherm of MAF-49.

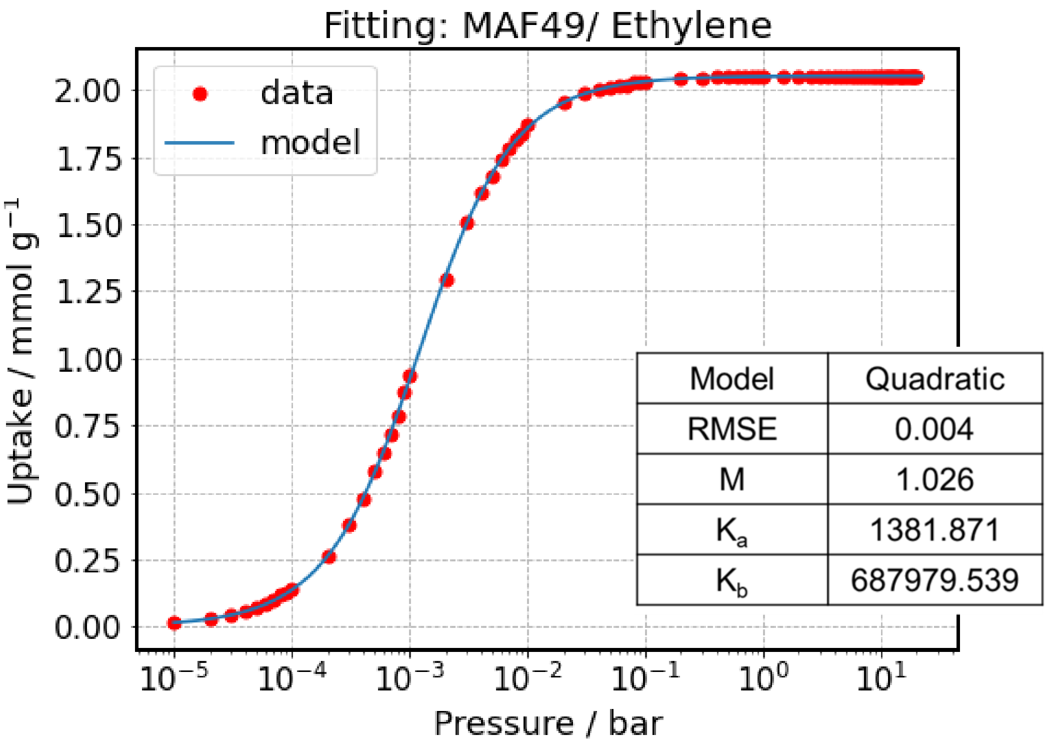

Figure S25. Model fit for simulated ethylene adsorption isotherm of MAF-49.

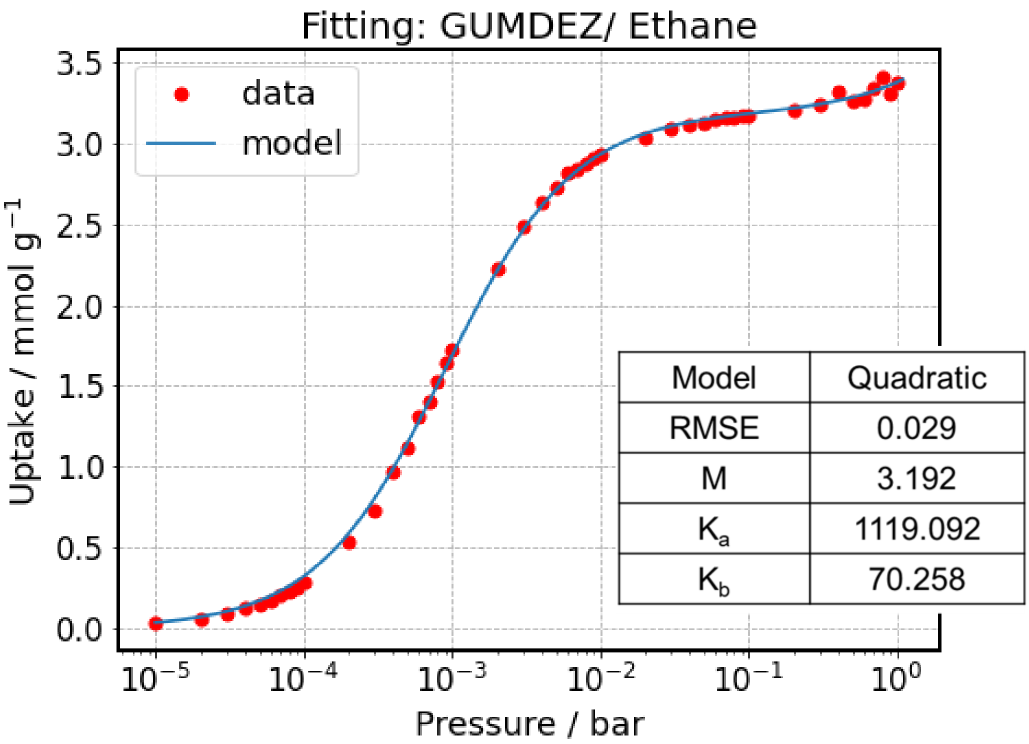

Figure S26. Model fit for simulated ethane adsorption isotherm of GUMDEZ.

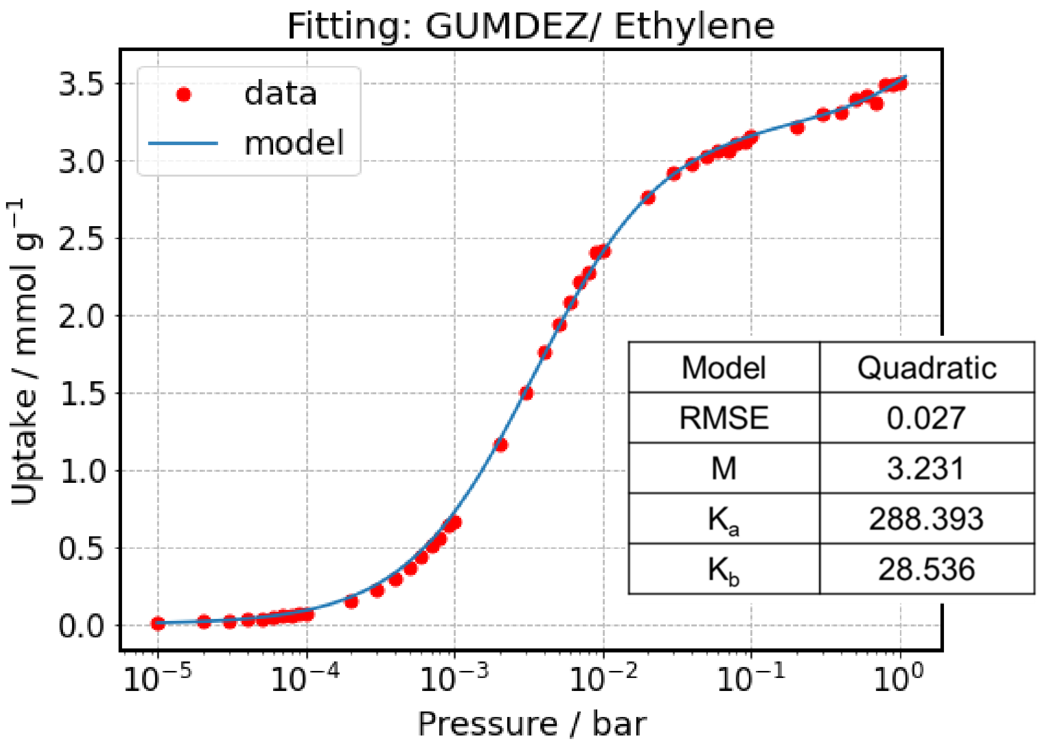

Figure S27. Model fit for simulated ethylene adsorption isotherm of GUMDEZ.

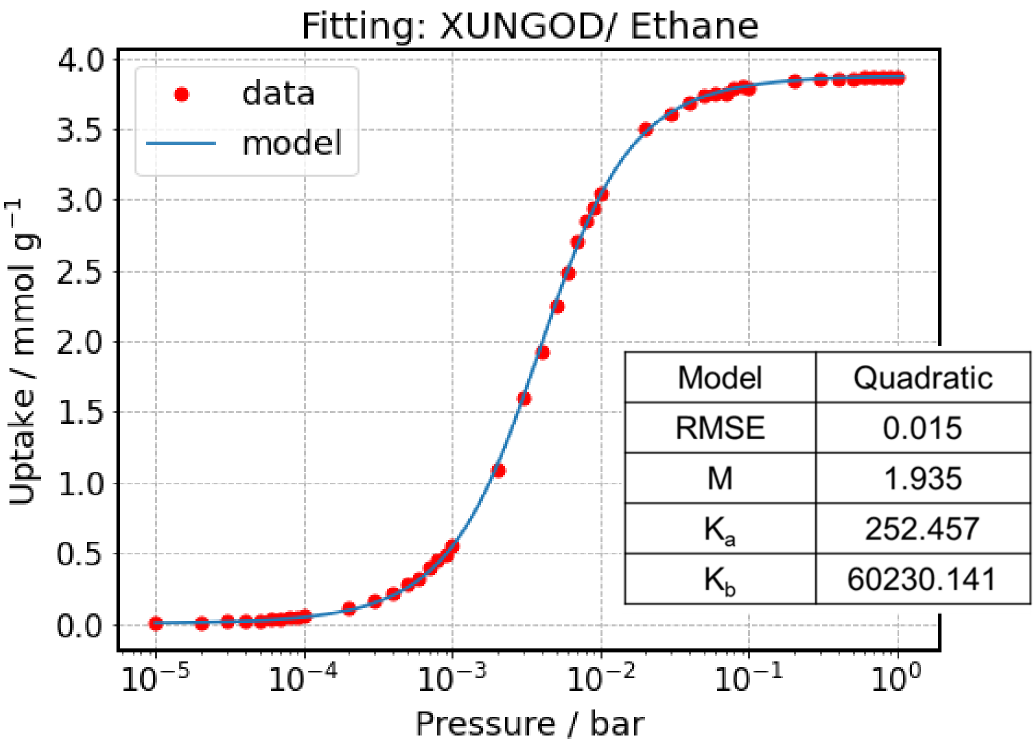

Figure S28. Model fit for simulated ethane adsorption isotherm of XUNGOD.

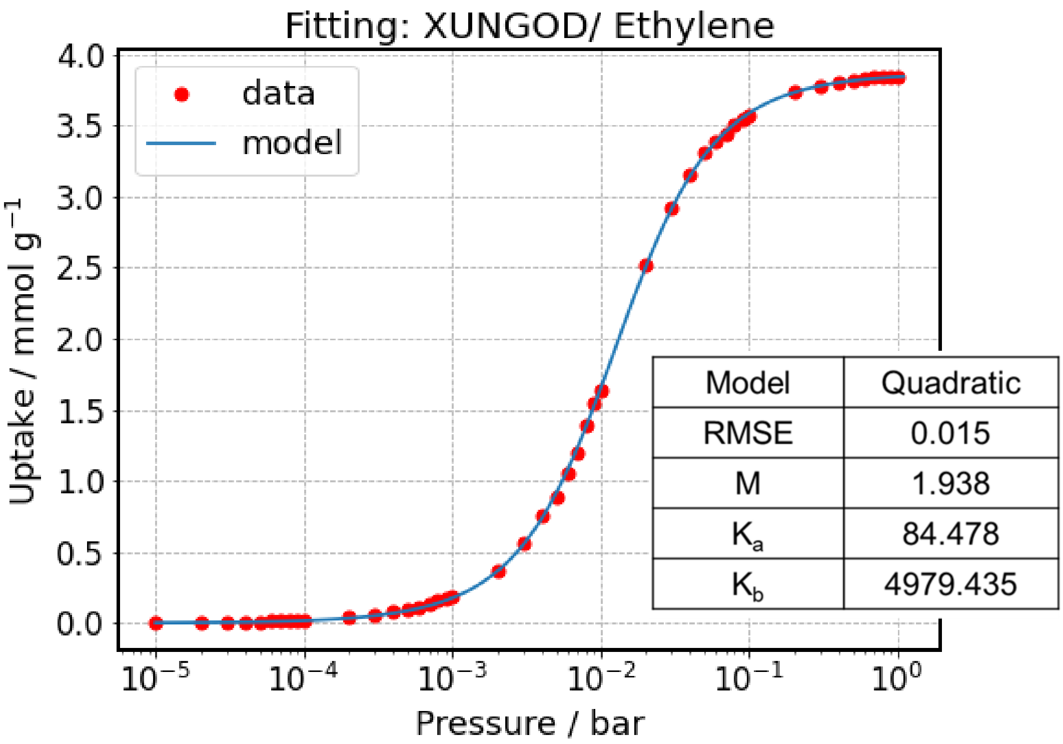

Figure S29. Model fit for simulated ethylene adsorption isotherm of XUNGOD.

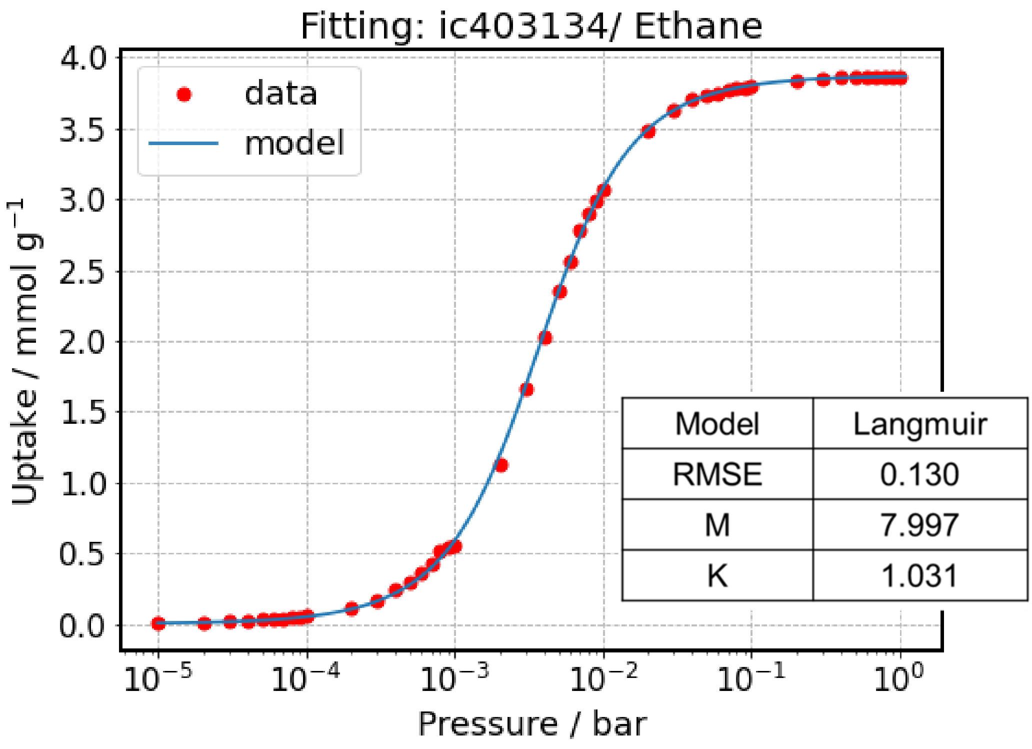

Figure S30. Model fit for simulated ethane adsorption isotherm of ic403134.

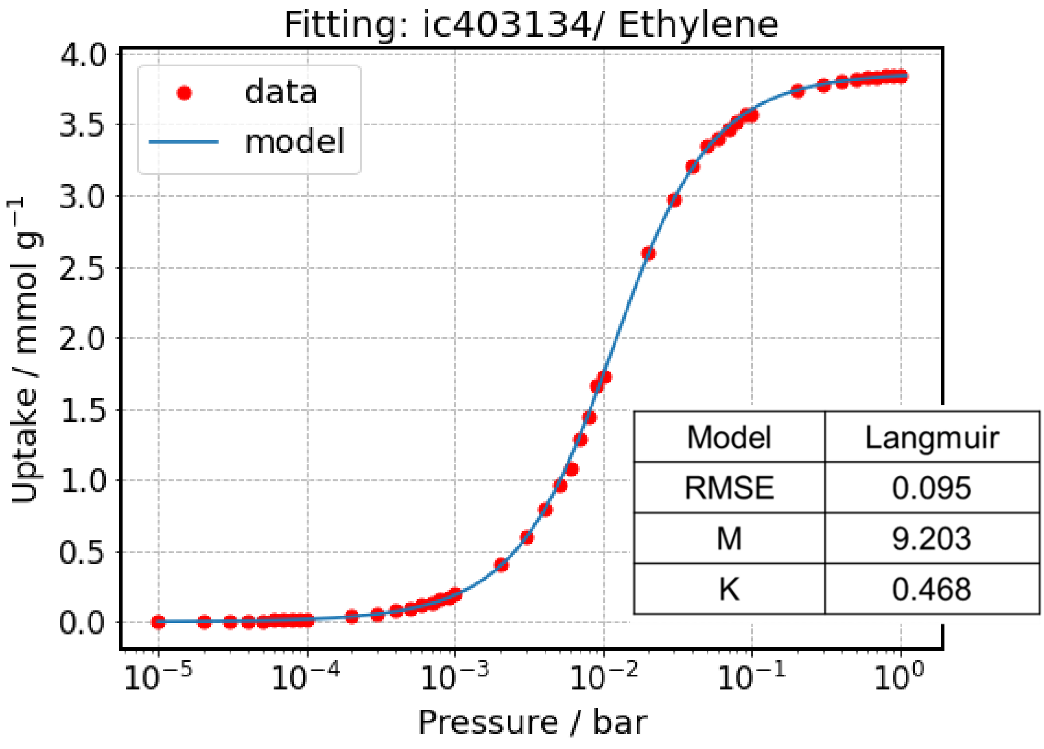

Figure S31. Model fit for simulated ethylene adsorption isotherm of ic403134.

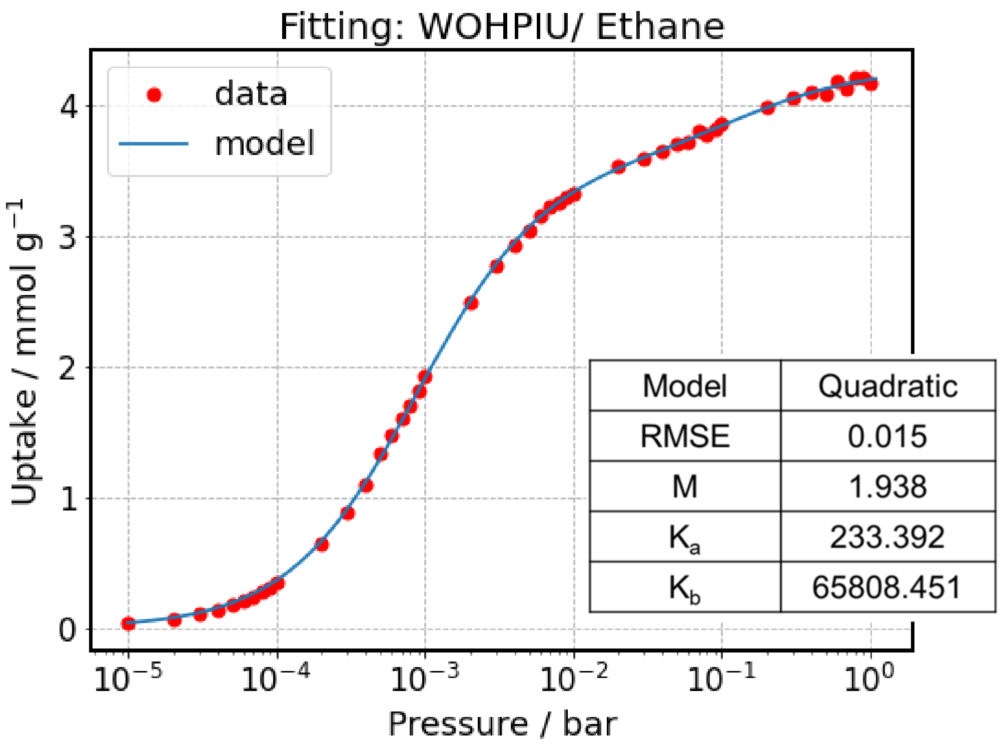

Figure S32. Model fit for simulated ethane adsorption isotherm of WOHPIU.

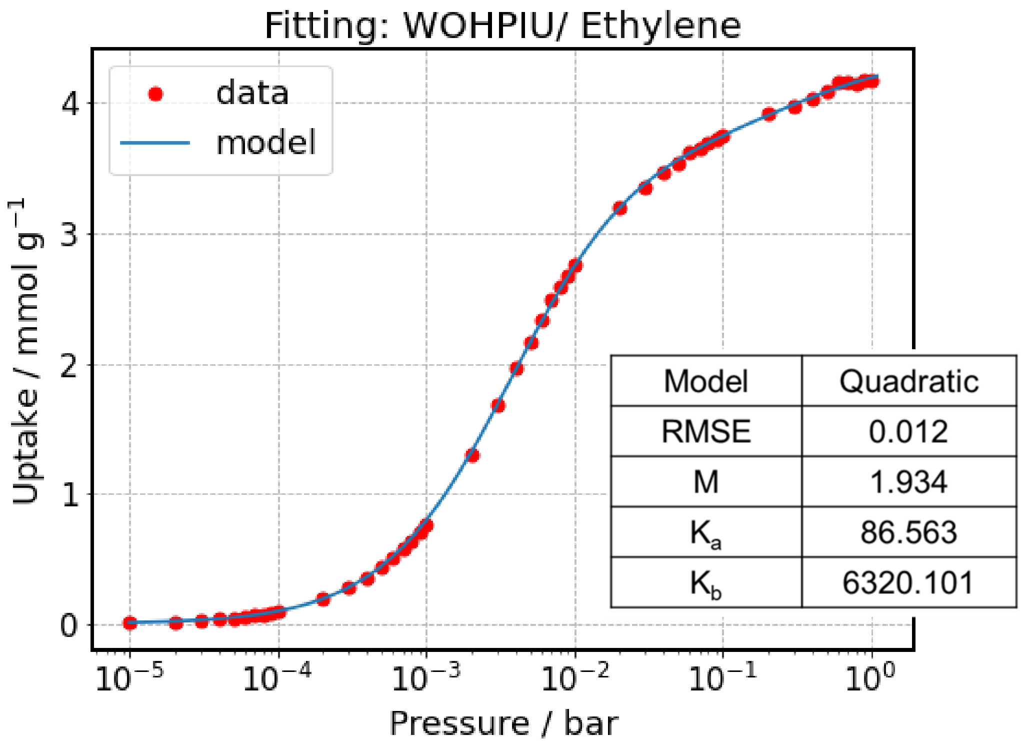

Figure S33. Model fit for simulated ethylene adsorption isotherm of WOHPIU.

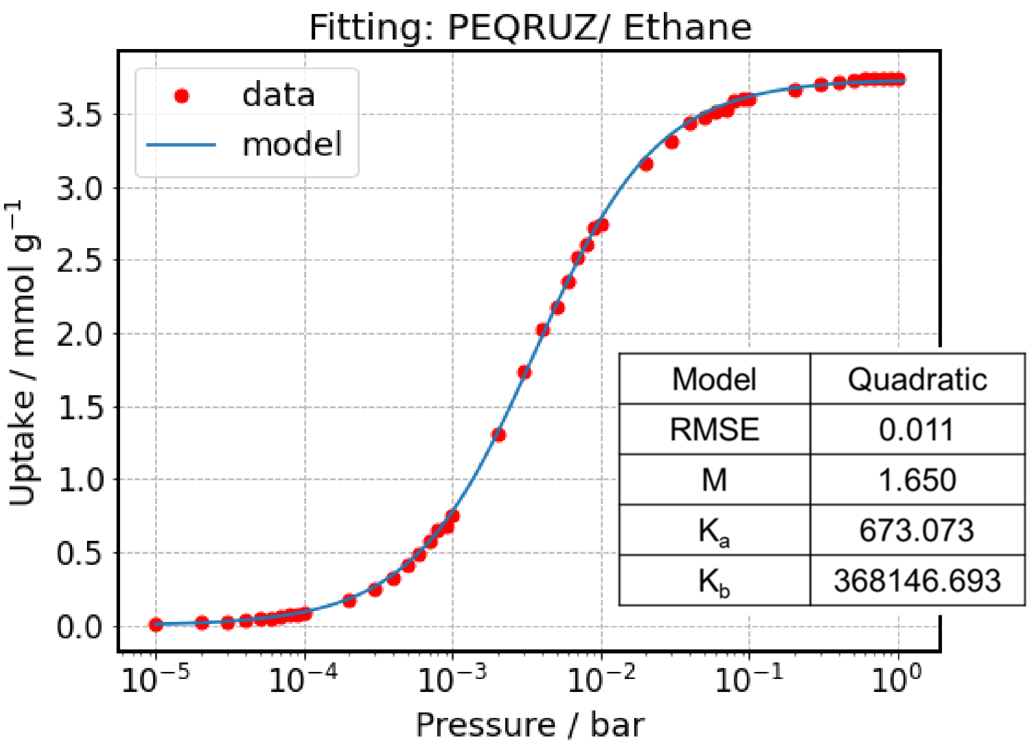

Figure S34. Model fit for simulated ethane adsorption isotherm of PEQRUZ.

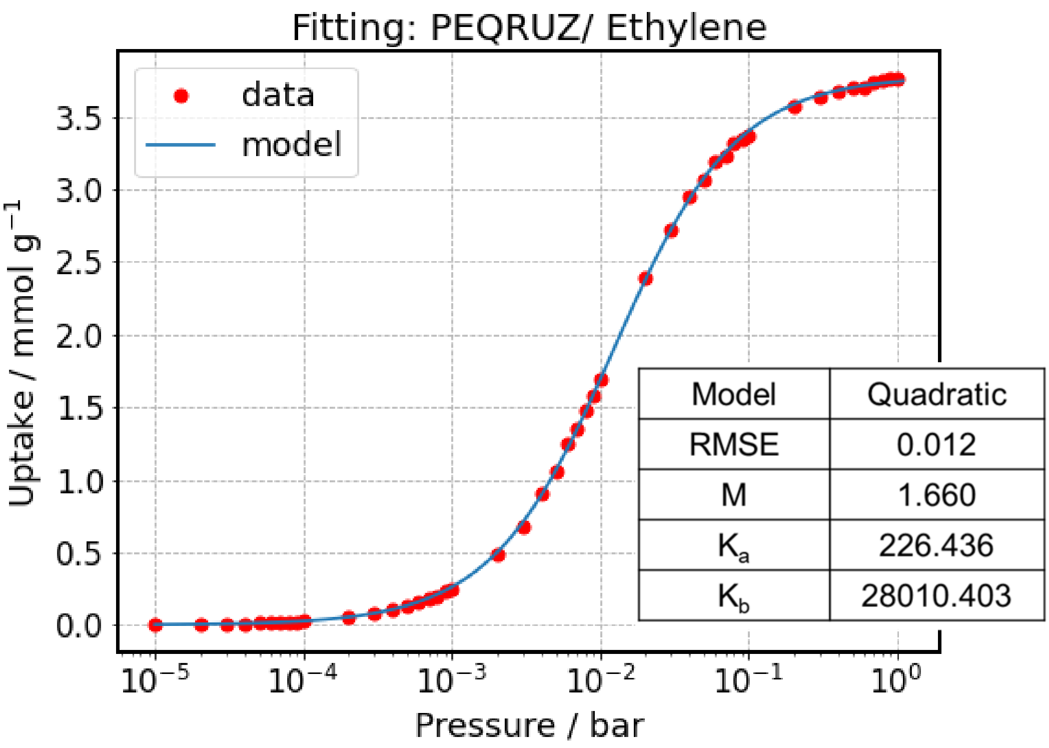

Figure S35. Model fit for simulated ethylene adsorption isotherm of PEQRUZ.

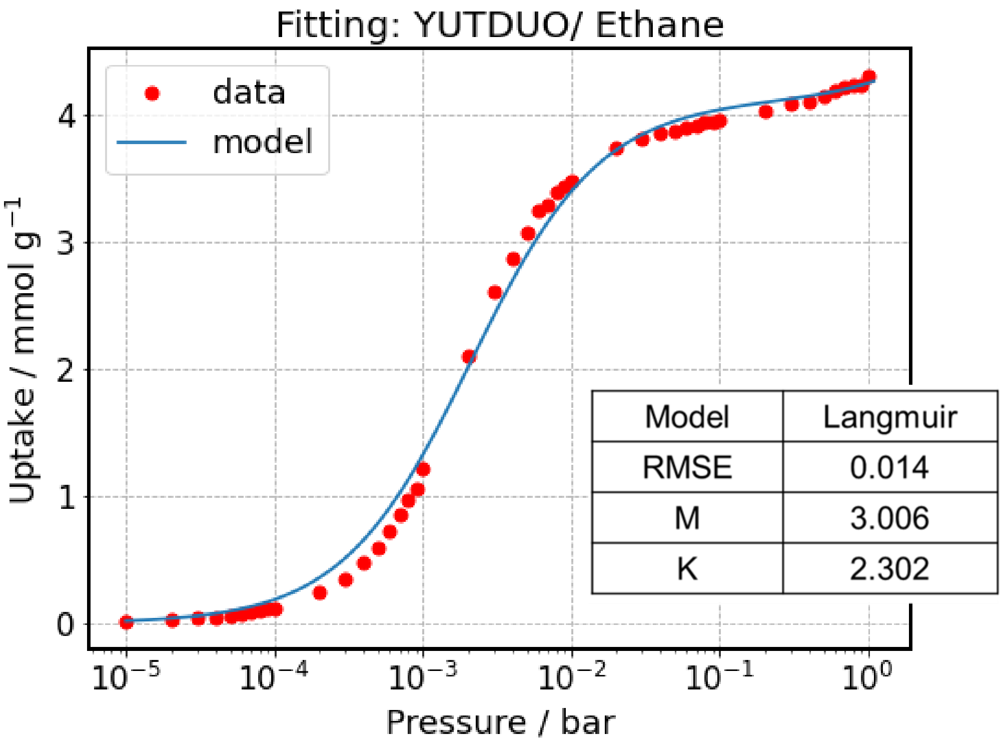

Figure S36. Model fit for simulated ethane adsorption isotherm of YUTDUO.

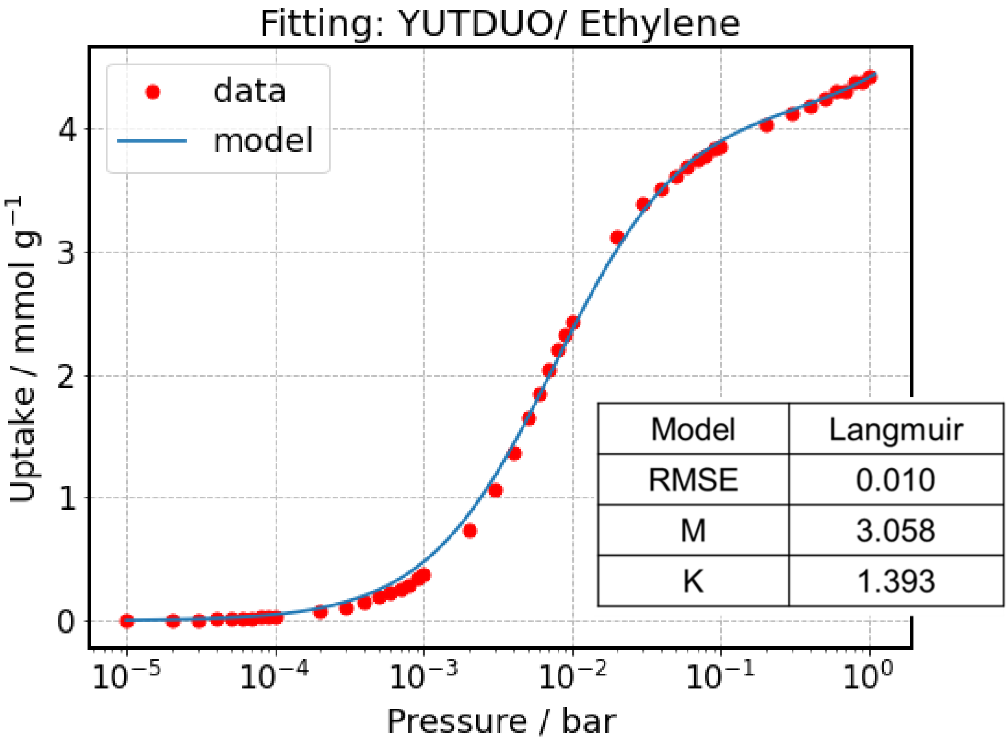

Figure S37. Model fit for simulated ethylene adsorption isotherm of YUTDUO.

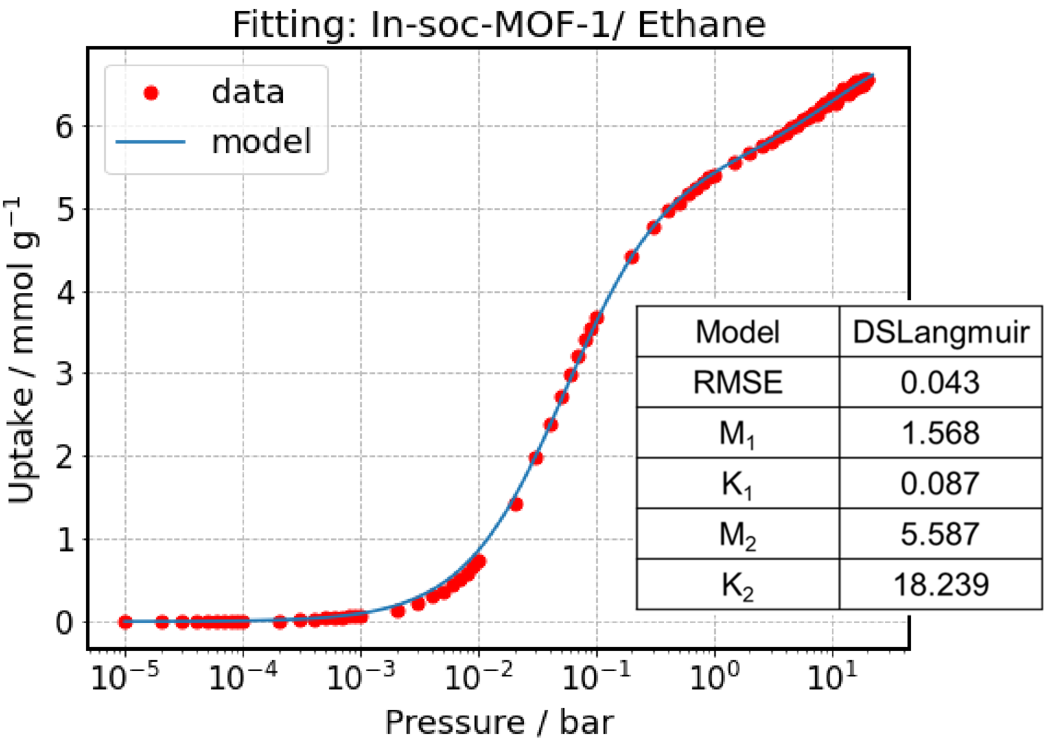

Figure S38. Model fit for simulated ethane adsorption isotherm of In-soc-MOF-1.

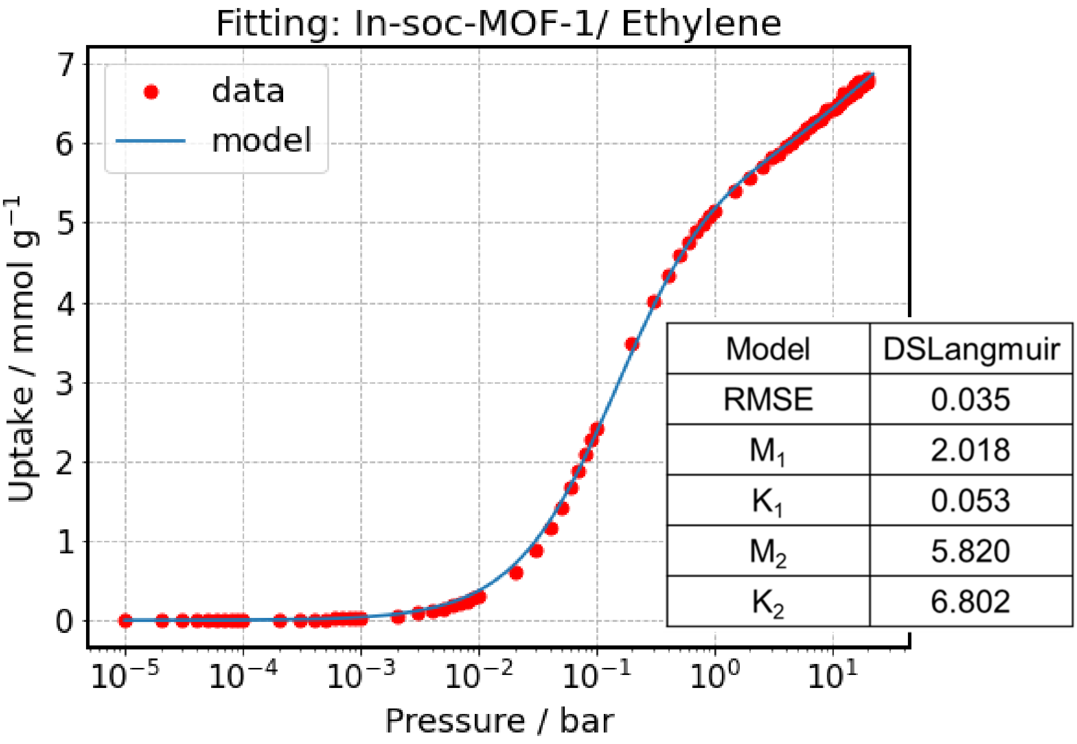

Figure S39. Model fit for simulated ethylene adsorption isotherm of In-soc-MOF-1.

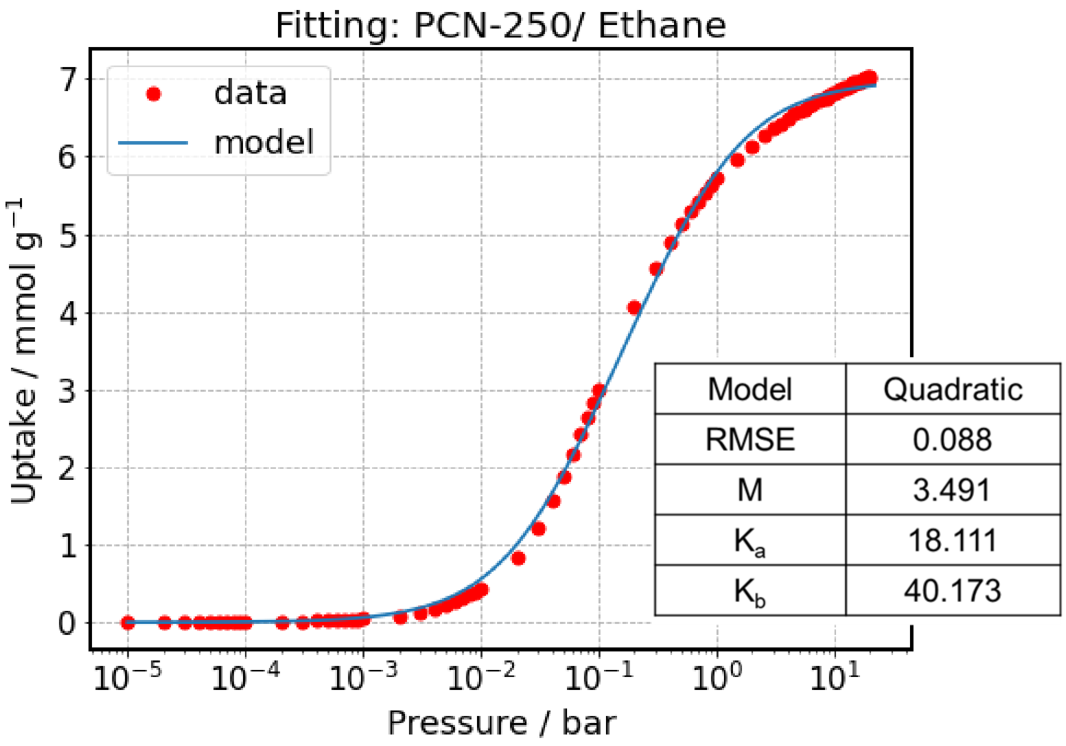

Figure S40. Model fit for simulated ethane adsorption isotherm of PCN-250.

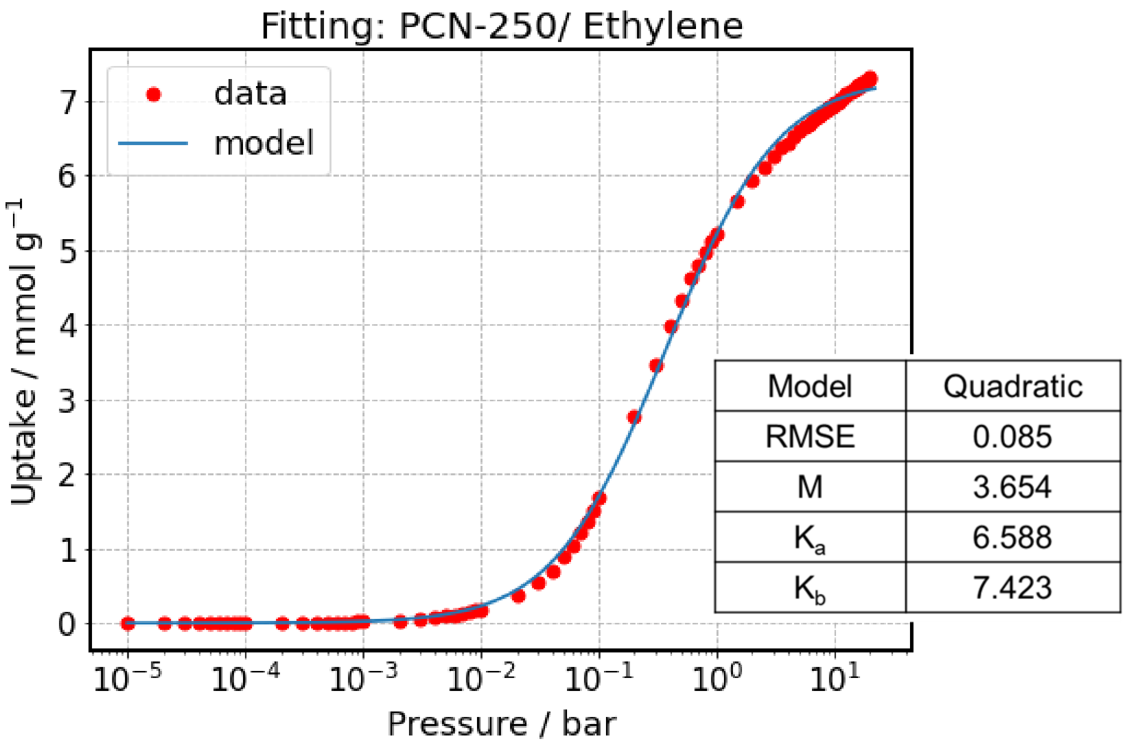

Figure S41. Model fit for simulated ethylene adsorption isotherm of PCN-250.

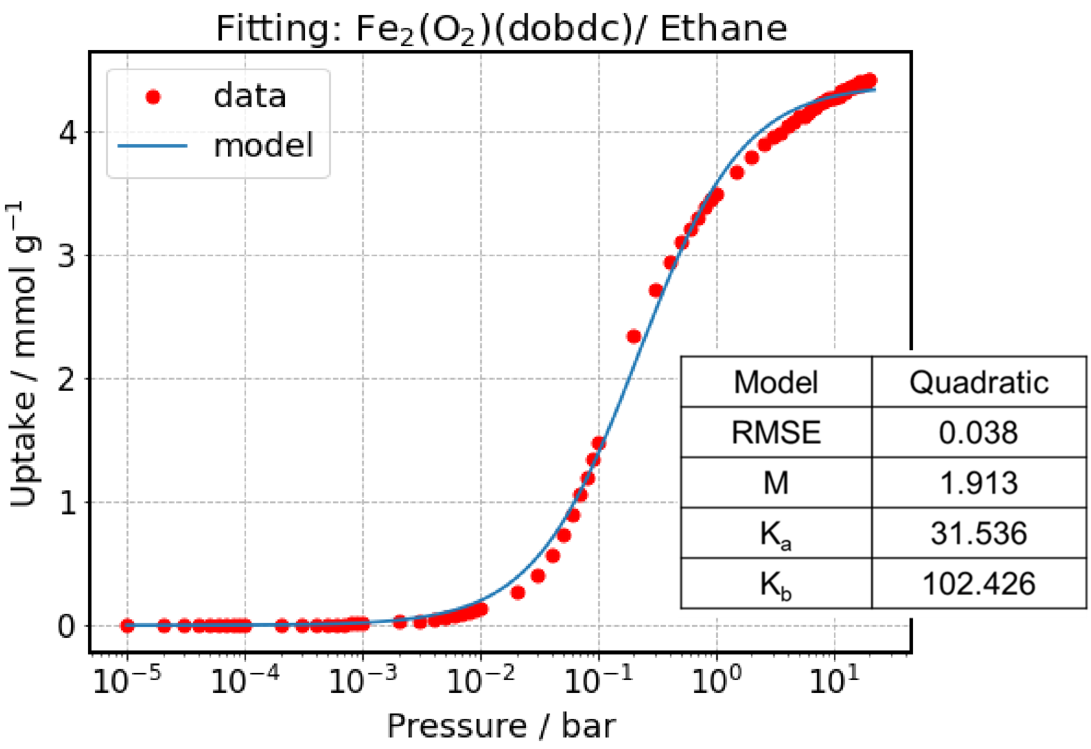

Figure S42. Model fit for simulated ethane adsorption isotherm of $Fe_2(O_2)$dobdc.

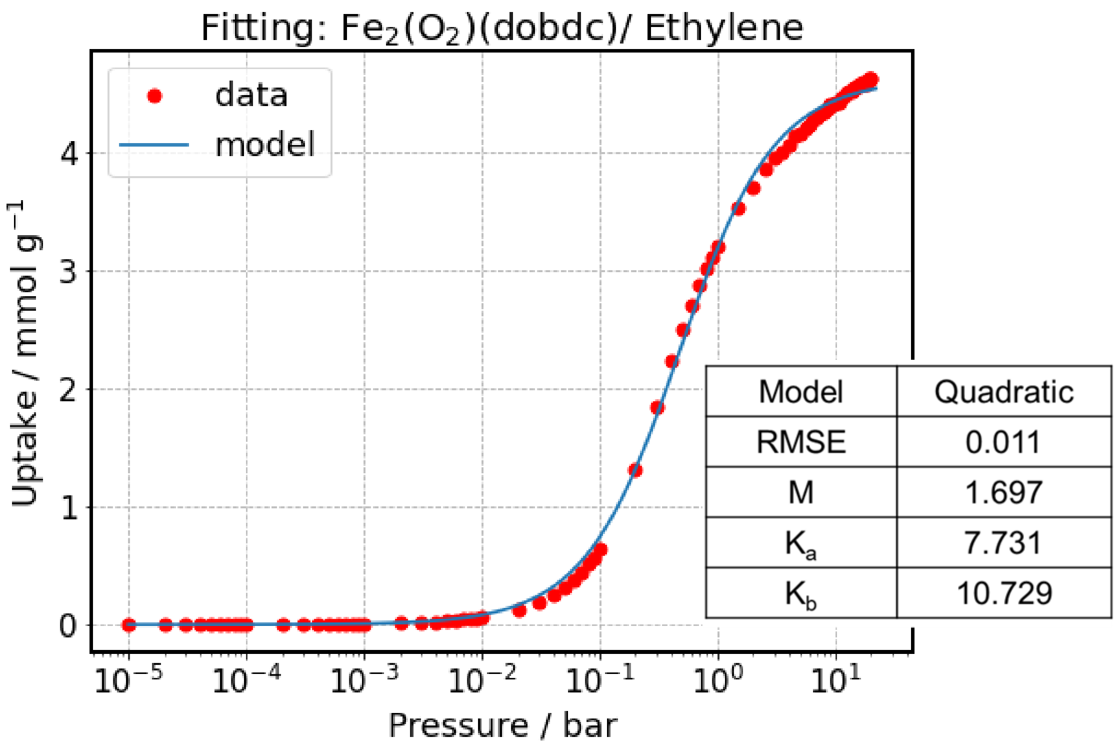

Figure S43. Model fit for simulated ethylene adsorption isotherm of $Fe_2(O_2)$dobdc.

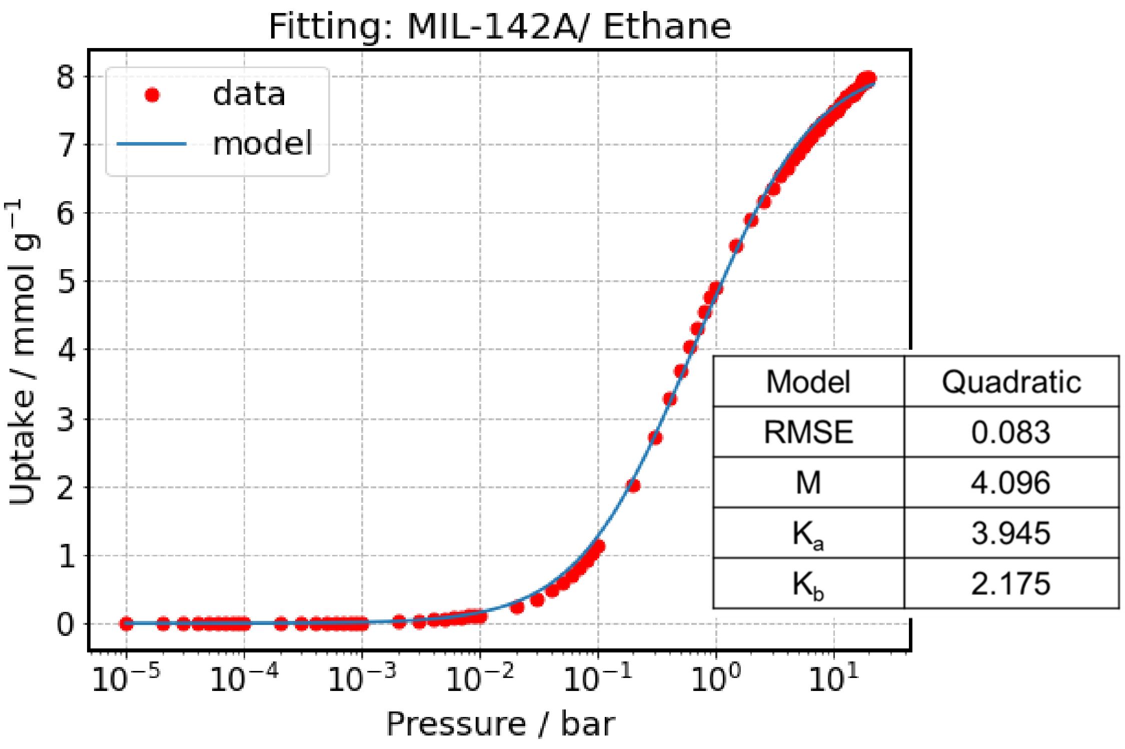

Model	Quadratic
RMSE	0.083
M	4.096
K_a	3.945
K_b	2.175

Figure S44. Model fit for simulated ethane adsorption isotherm of MIL-142A.

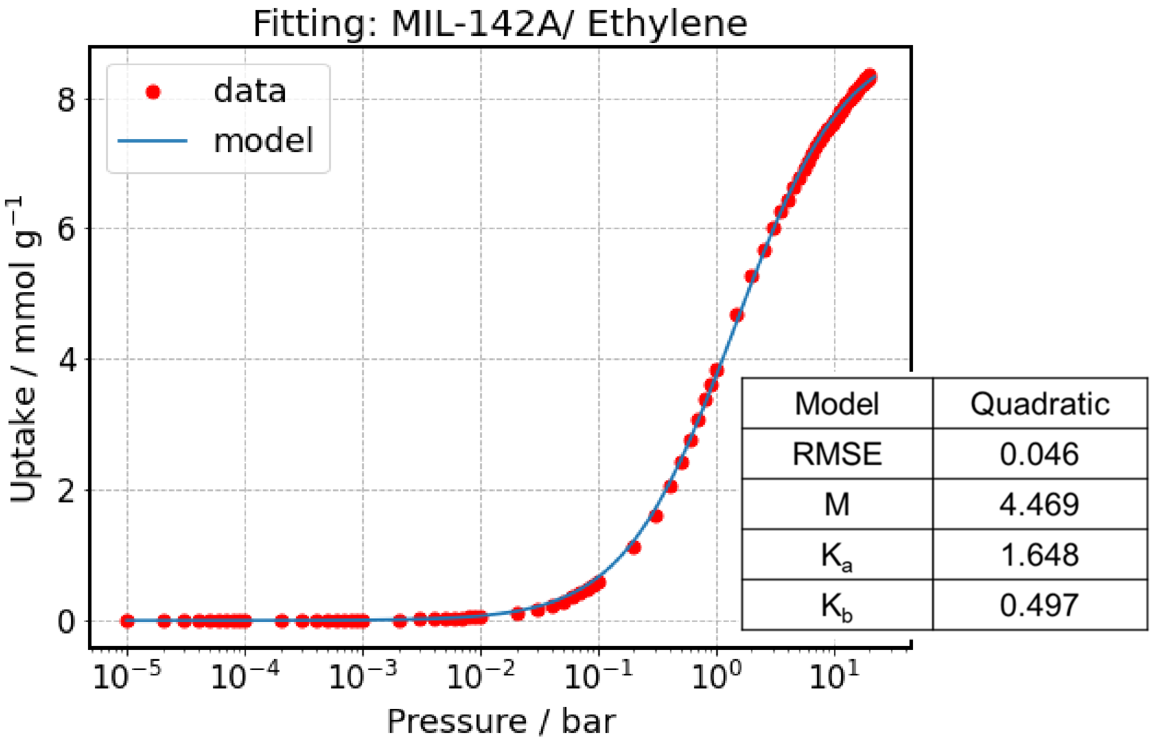

Model	Quadratic
RMSE	0.046
M	4.469
K_a	1.648
K_b	0.497

Figure S45. Model fit for simulated ethylene adsorption isotherm of MIL-142A.

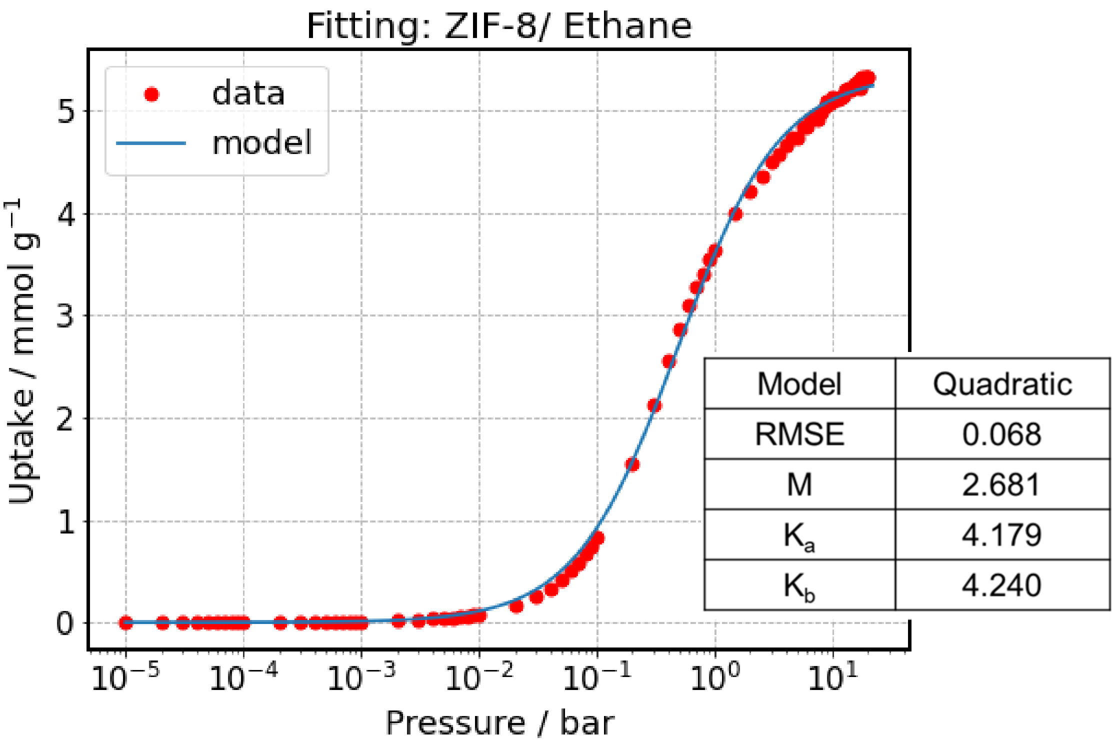

Figure S46. Model fit for simulated ethane adsorption isotherm of ZIF-8.

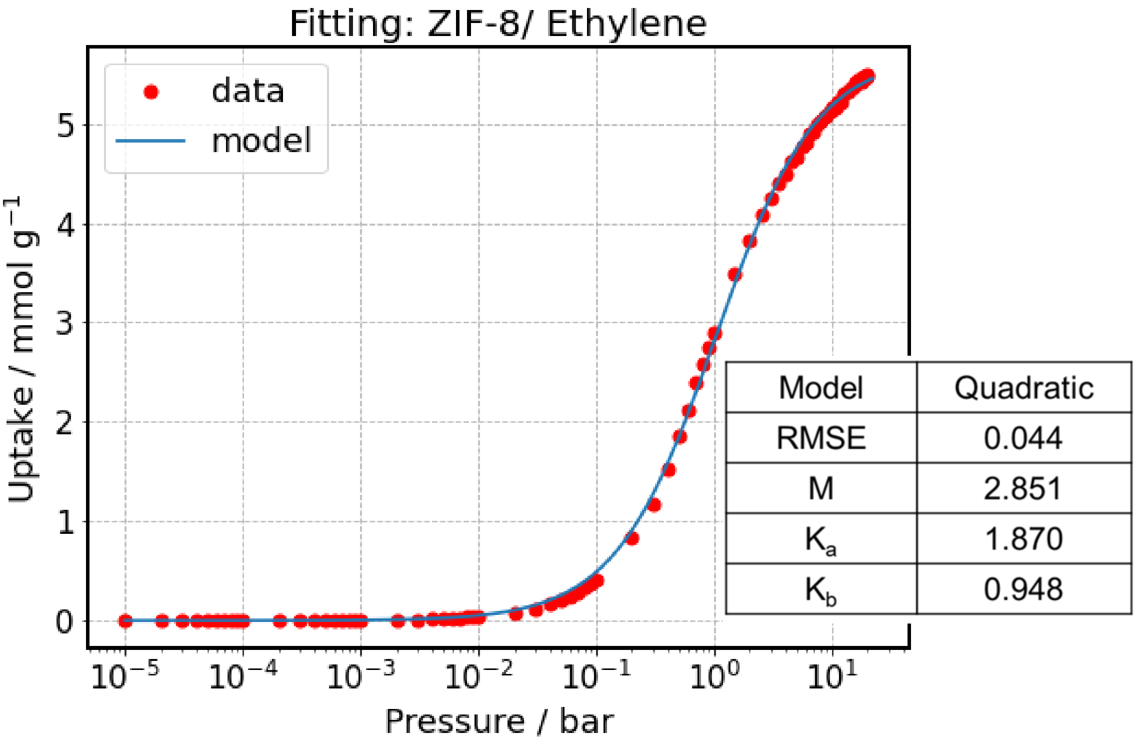

Figure S47. Model fit for simulated ethylene adsorption isotherm of ZIF-8.

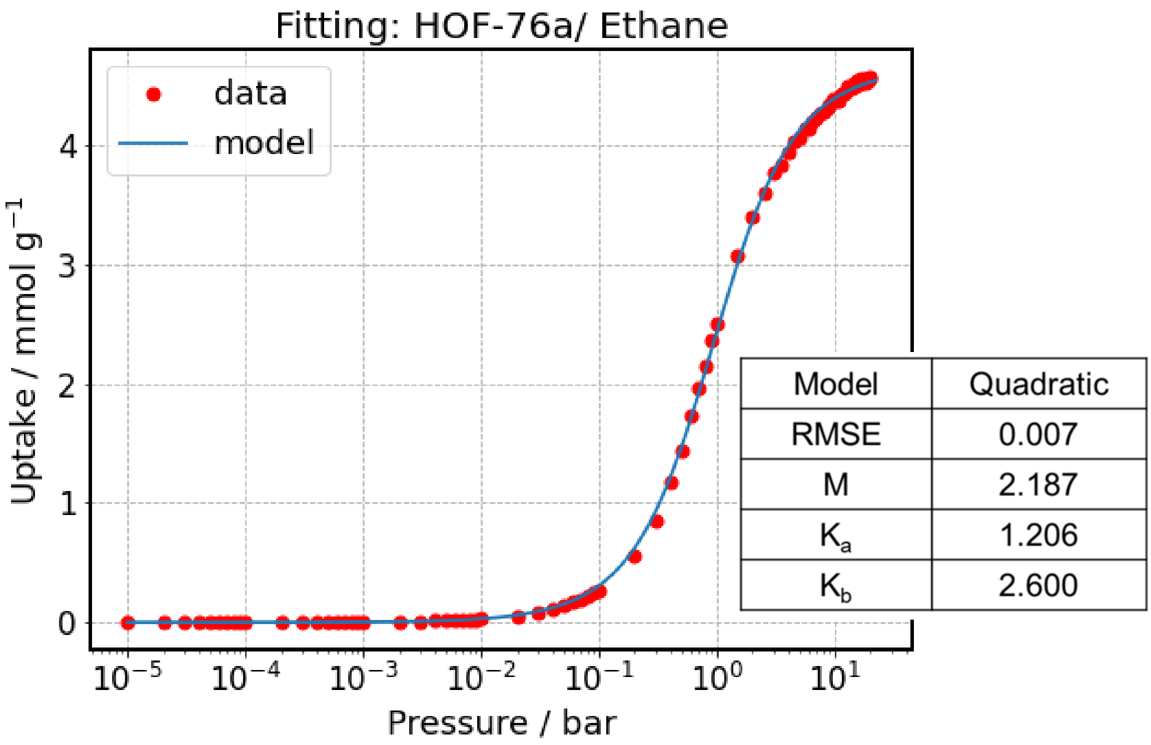

Figure S48. Model fit for simulated ethane adsorption isotherm of HOF-76a.

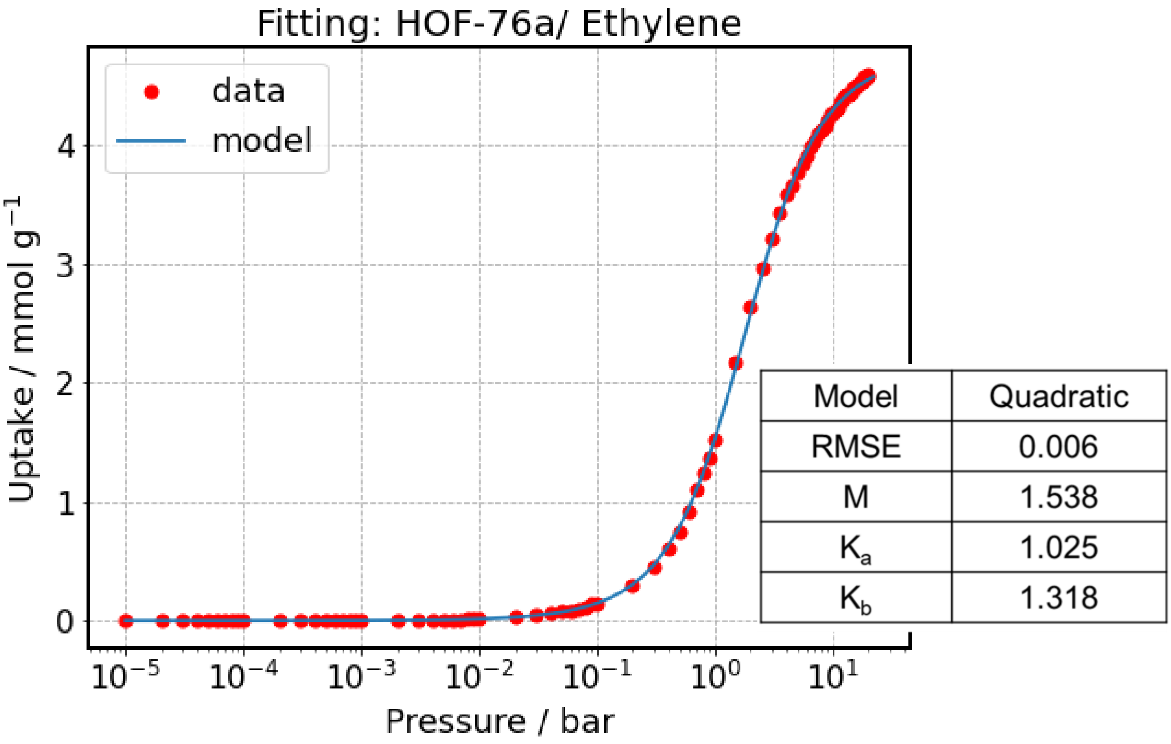

Figure S49. Model fit for simulated ethylene adsorption isotherm of HOF-76a.

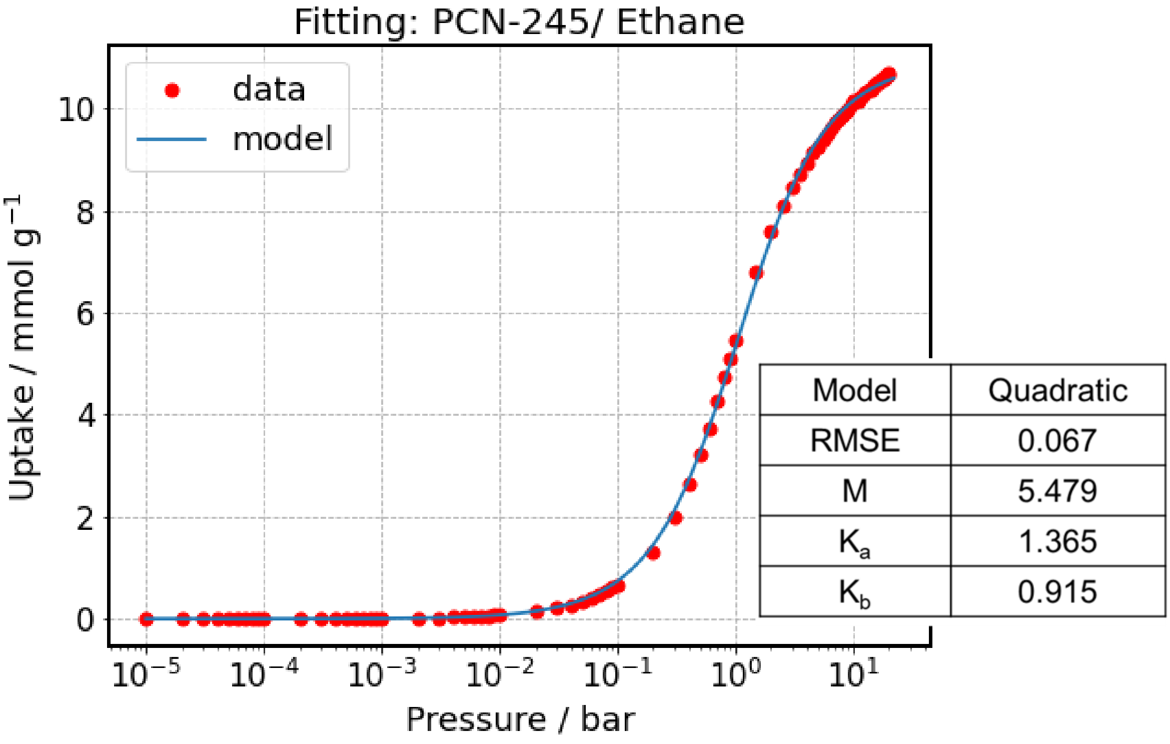

Model	Quadratic
RMSE	0.067
M	5.479
K_a	1.365
K_b	0.915

Figure S50. Model fit for simulated ethane adsorption isotherm of PCN-245.

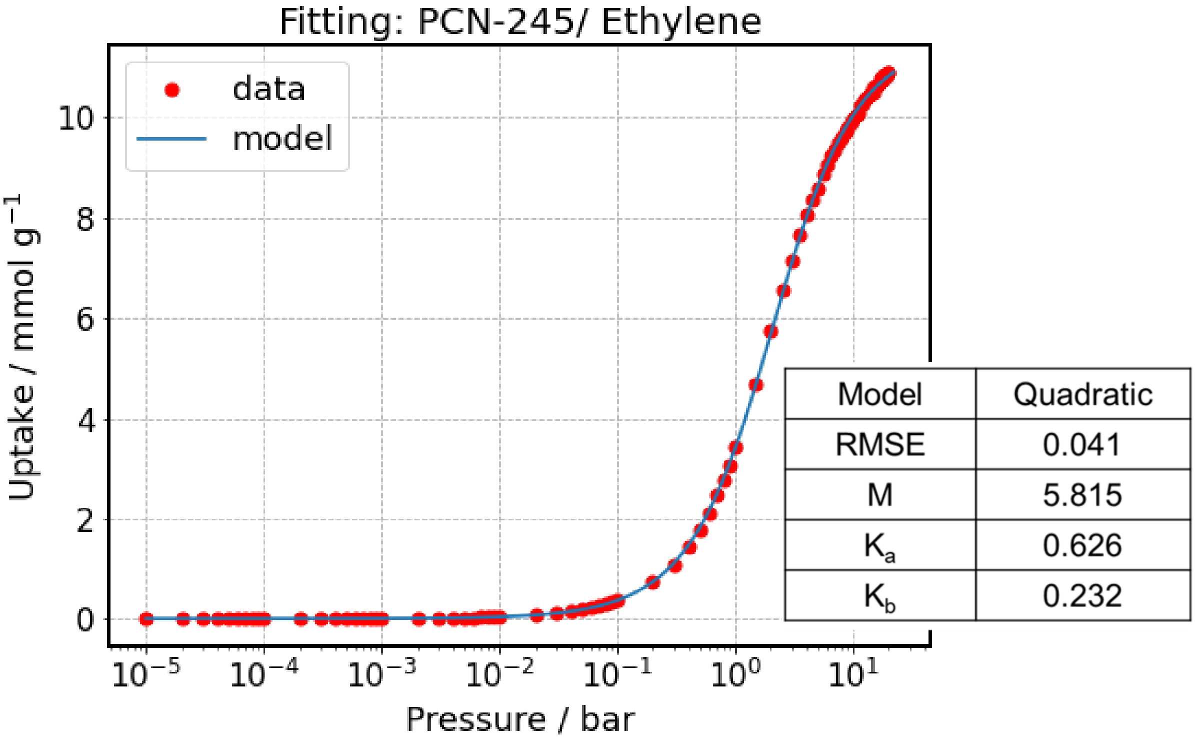

Model	Quadratic
RMSE	0.041
M	5.815
K_a	0.626
K_b	0.232

Figure S51. Model fit for simulated ethylene adsorption isotherm of PCN-245.

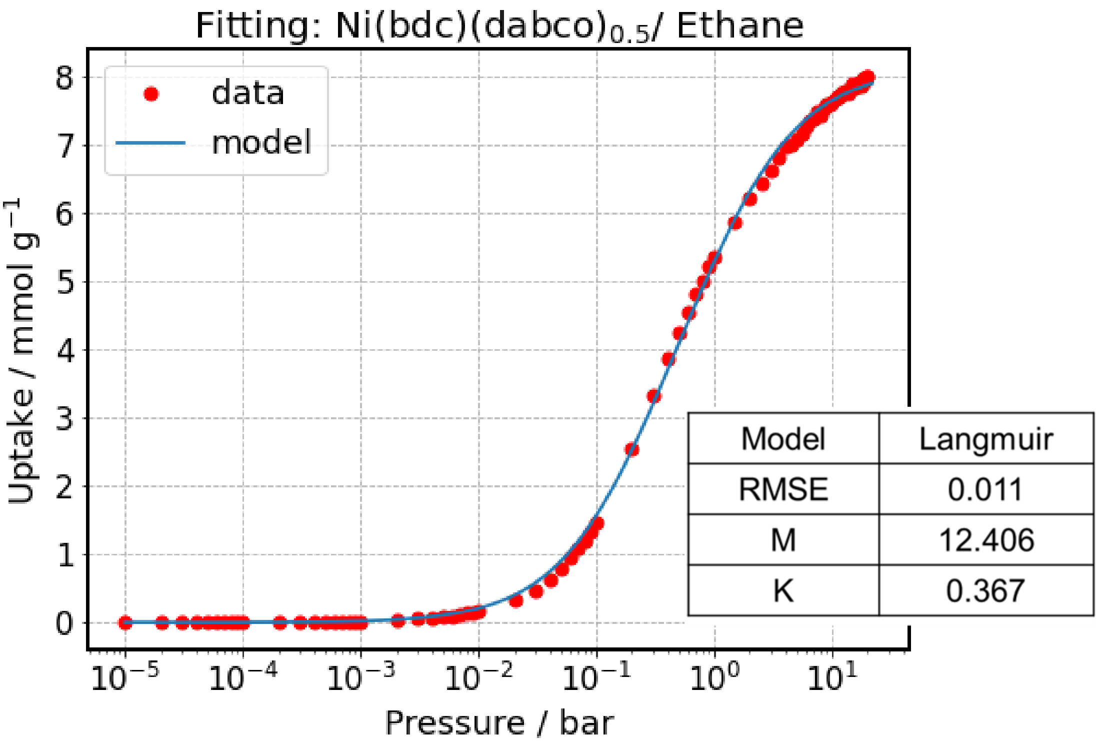

Figure S52. Model fit for simulated ethane adsorption isotherm of Ni(bdc)(dabco)$_{0.5}$.

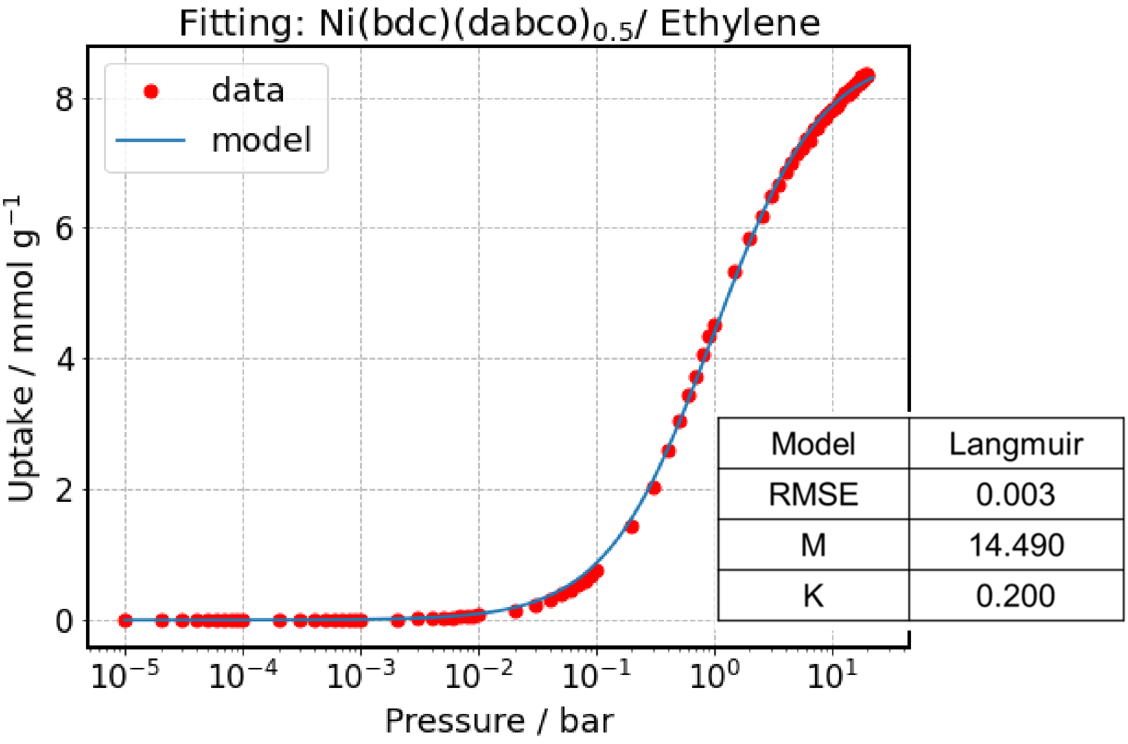

Figure S53. Model fit for simulated ethylene adsorption isotherm of Ni(bdc)(dabco)$_{0.5}$.

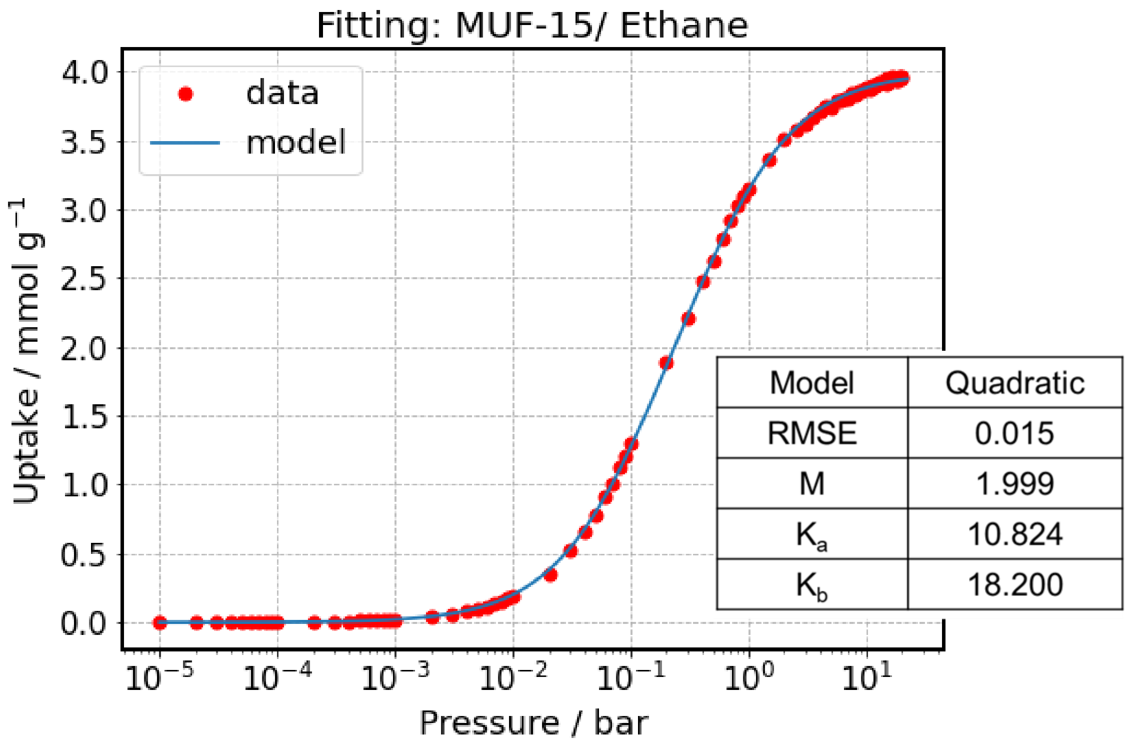

Figure S54. Model fit for simulated ethane adsorption isotherm of MUF-15.

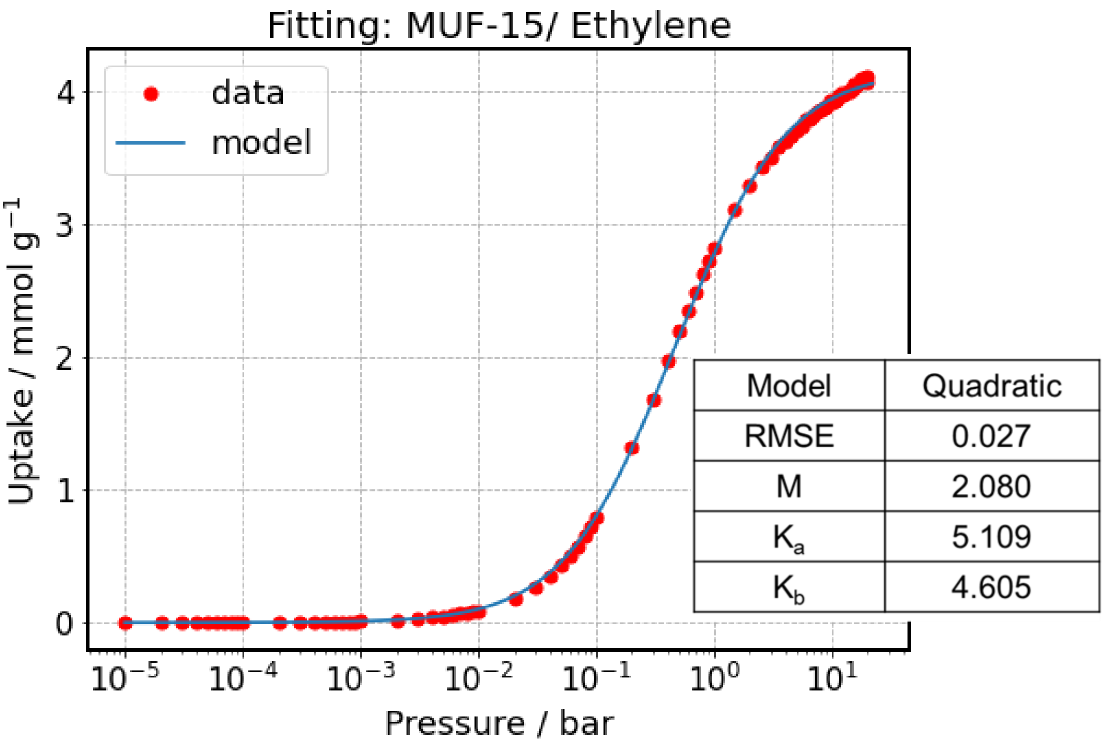

Figure S55. Model fit for simulated ethylene adsorption isotherm of MUF-15.

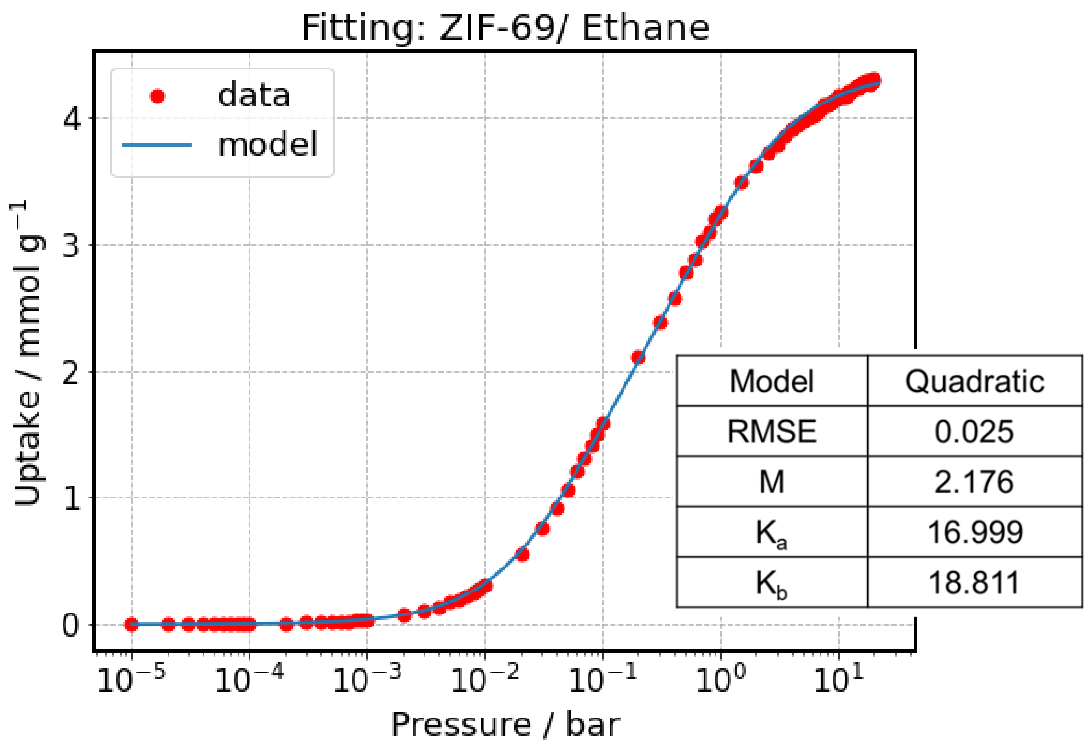

Figure S56. Model fit for simulated ethane adsorption isotherm of ZIF-69.

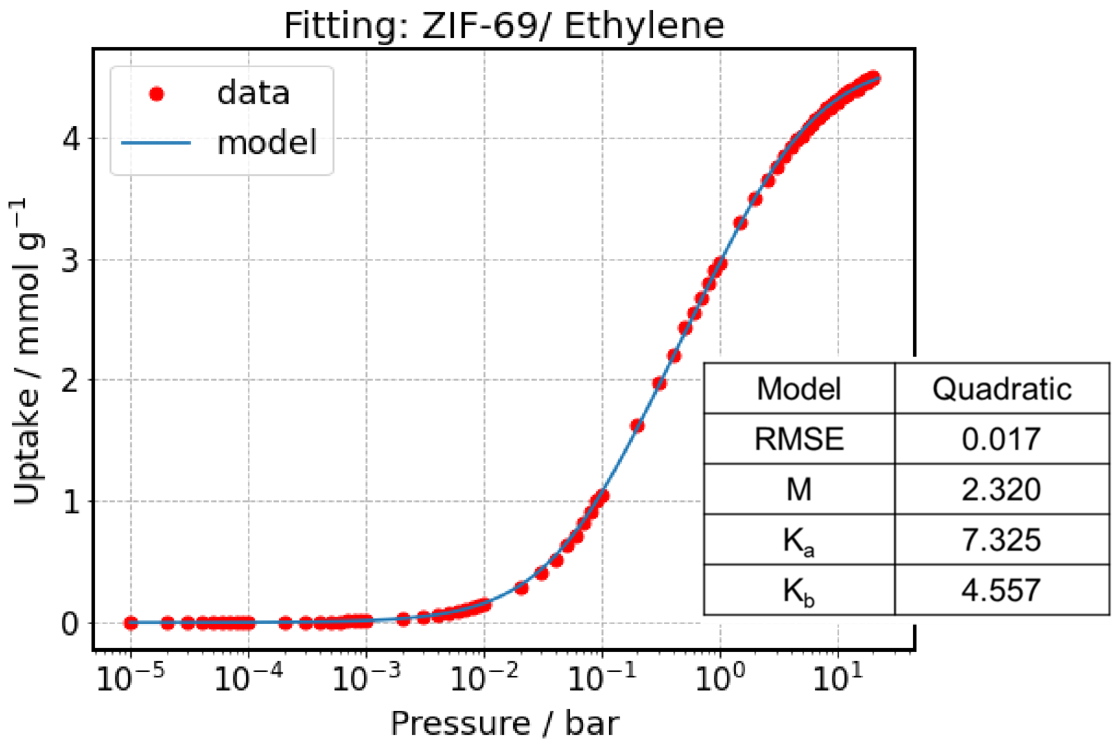

Figure S57. Model fit for simulated ethylene adsorption isotherm of ZIF-69.

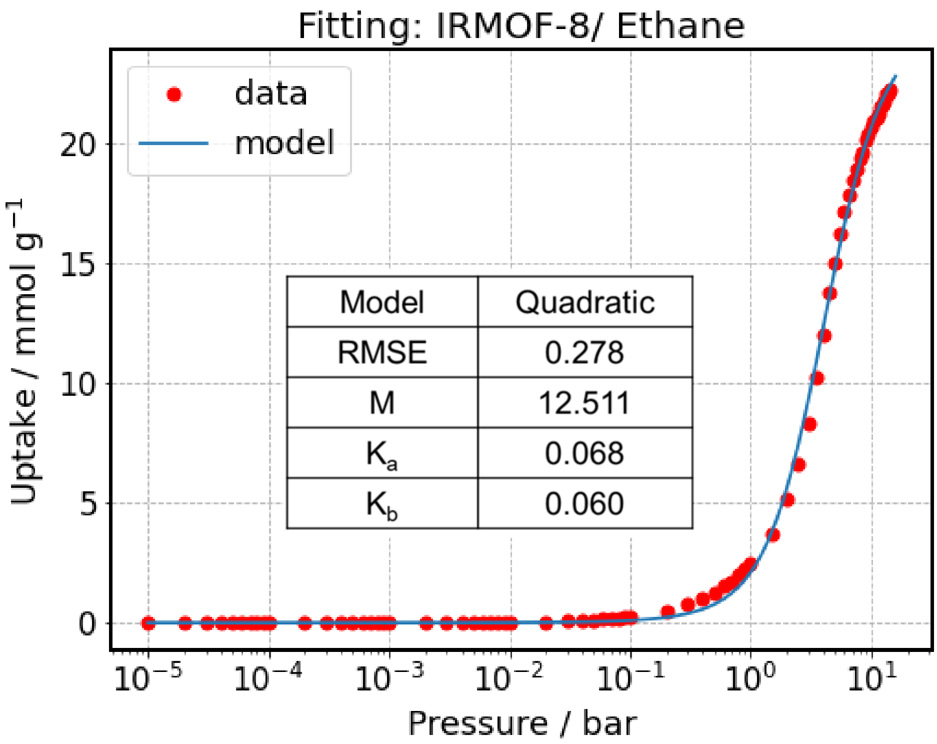

Figure S58. Model fit for simulated ethane adsorption isotherm of IRMOF-8.

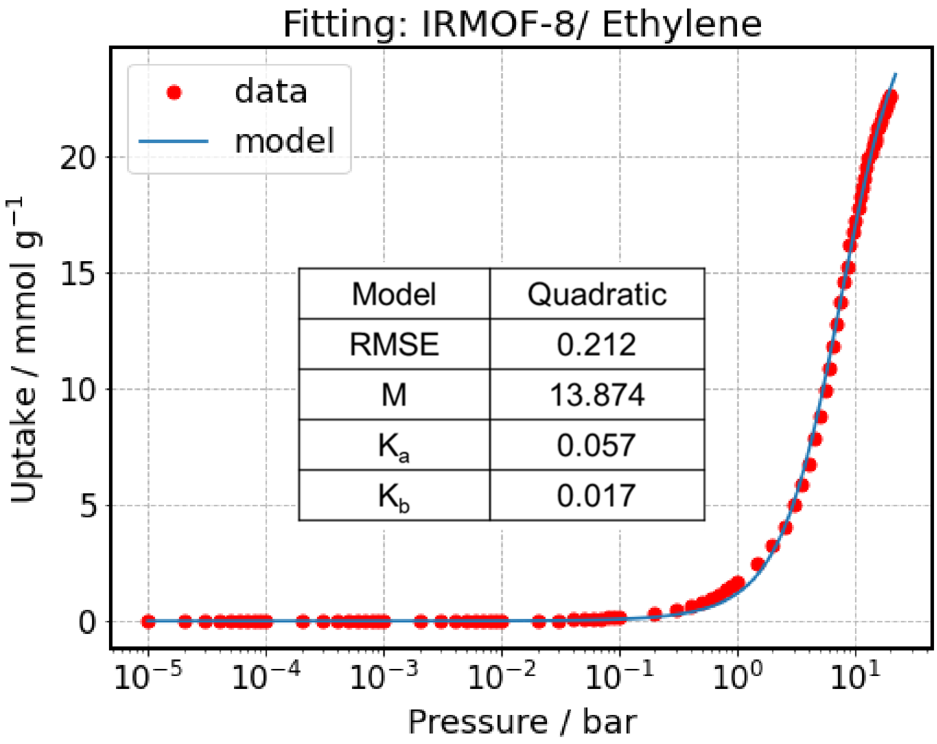

Figure S59. Model fit for simulated ethylene adsorption isotherm of IRMOF-8.

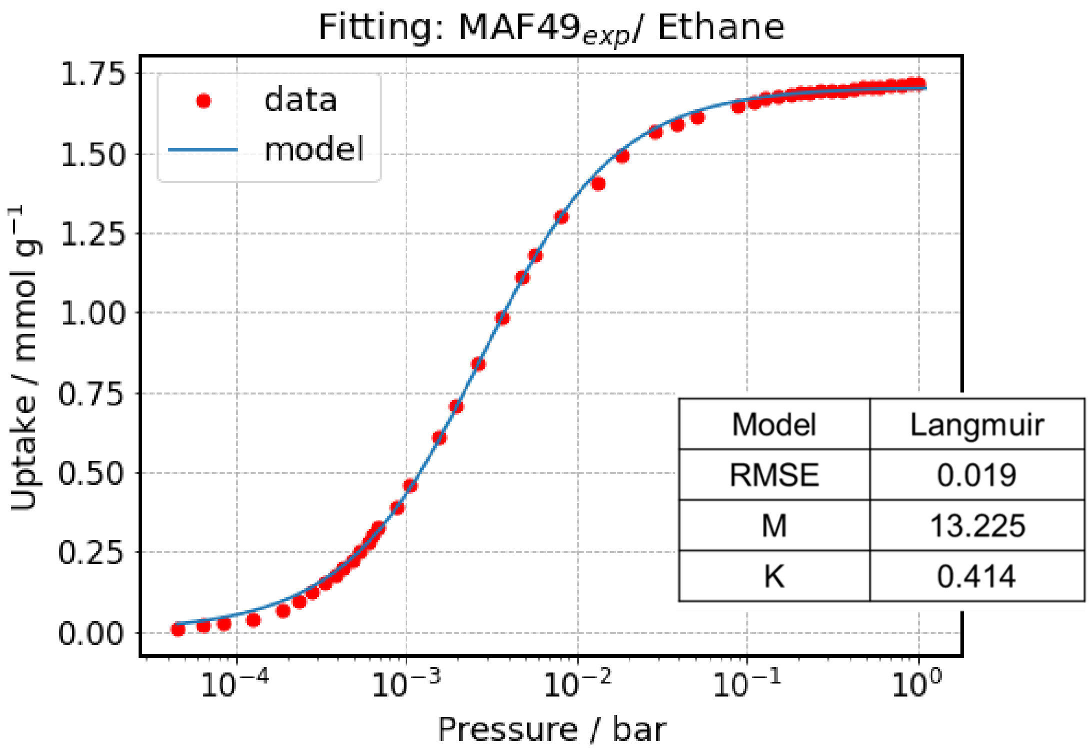

Figure S60. Model fit for experimental ethane adsorption isotherm of MAF-49.

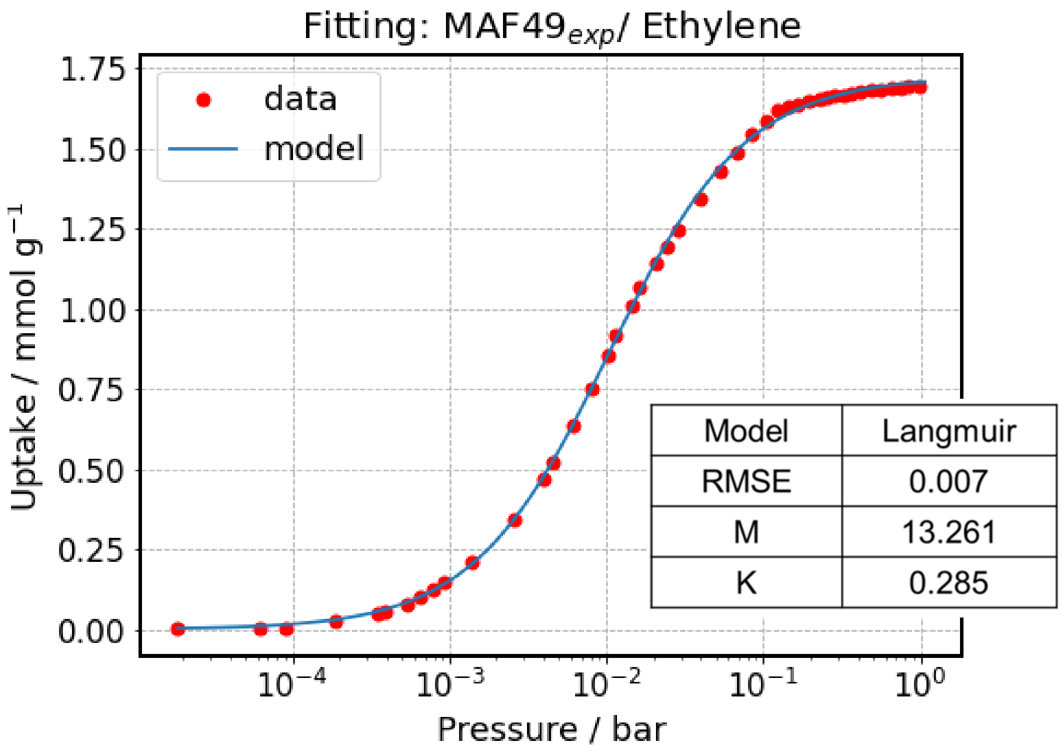

Figure S61. Model fit for experimental ethylene adsorption isotherm of MAF-49.

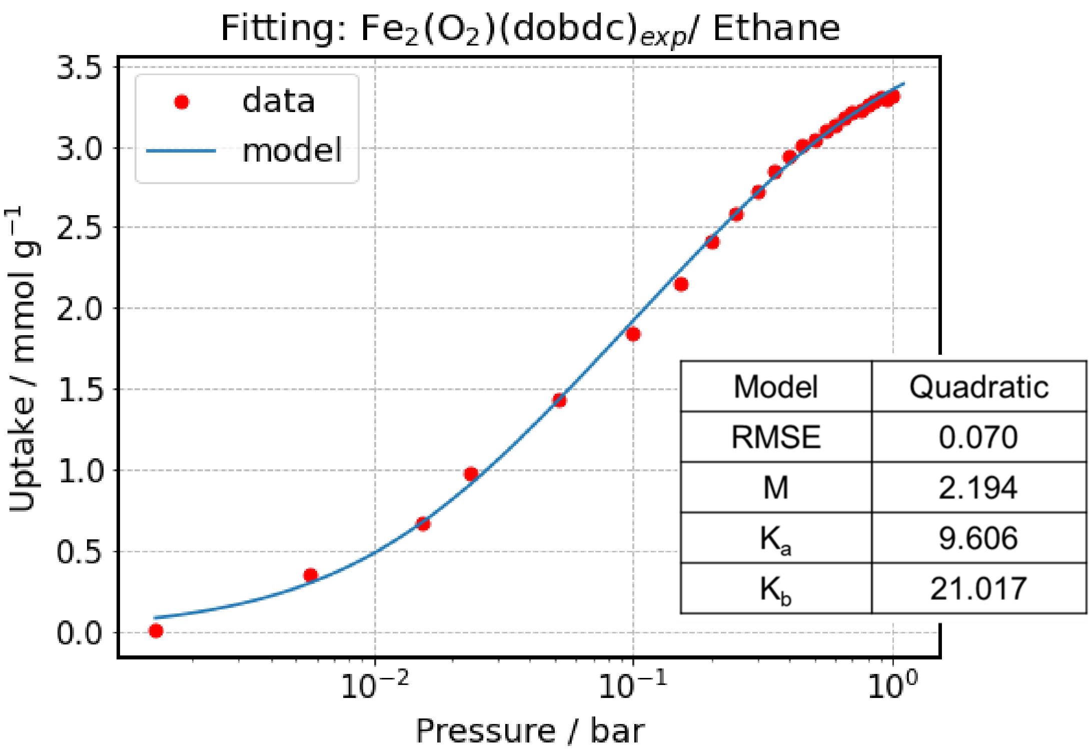

Figure S62. Model fit for experimental ethane adsorption isotherm of $Fe_2(O_2)$(dobdc).

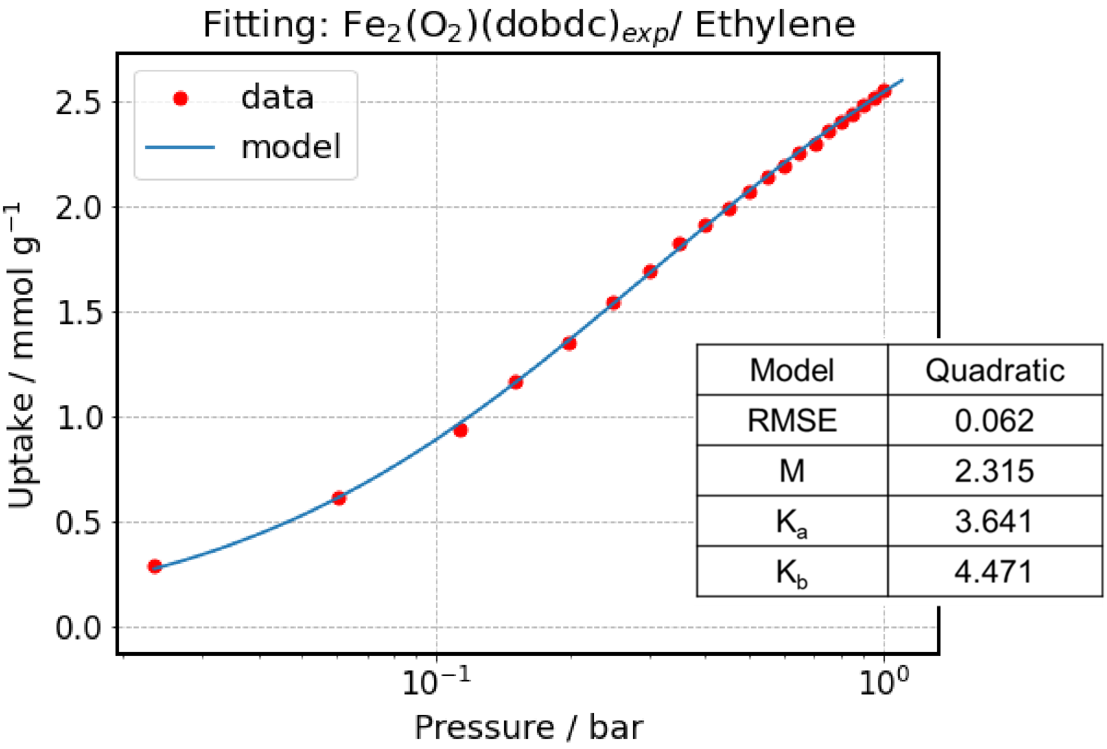

Figure S63. Model fit for experimental ethylene adsorption isotherm of $Fe_2(O_2)$(dobdc).

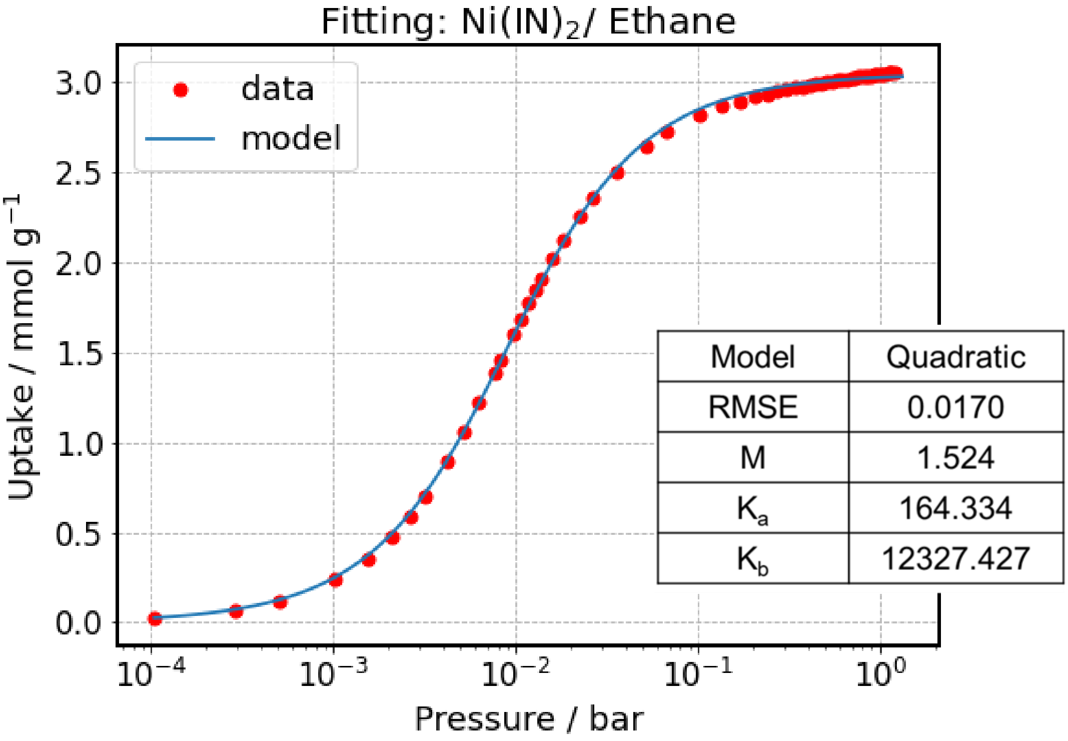

Figure S64. Model fit for experimental ethane adsorption isotherm of **Ni(IN)$_2$**.

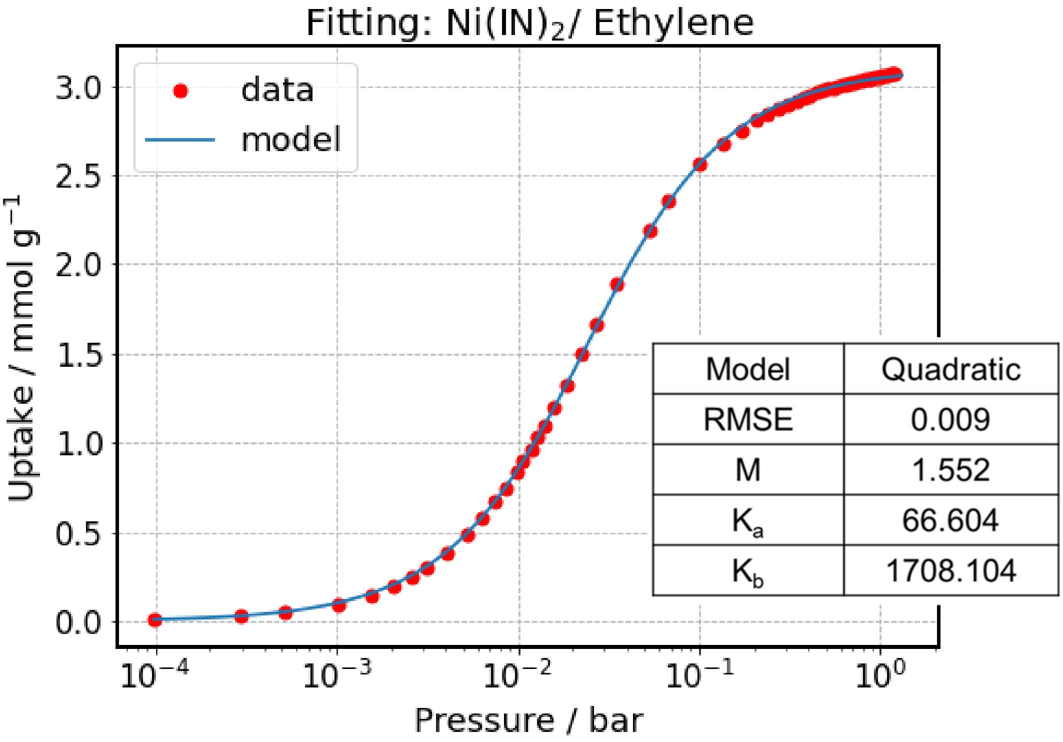

Figure S65. Model fit for experimental ethylene adsorption isotherm of **Ni(IN)$_2$**.

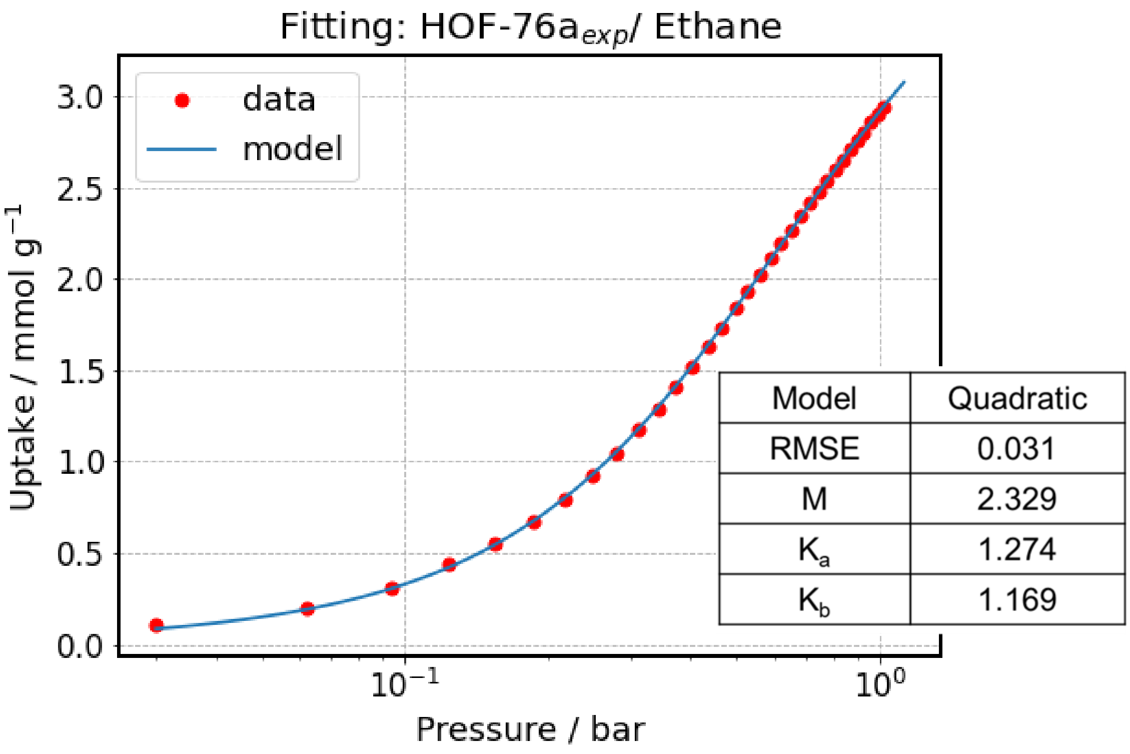

Figure S66. Model fit for experimental ethane adsorption isotherm of HOF-76a.

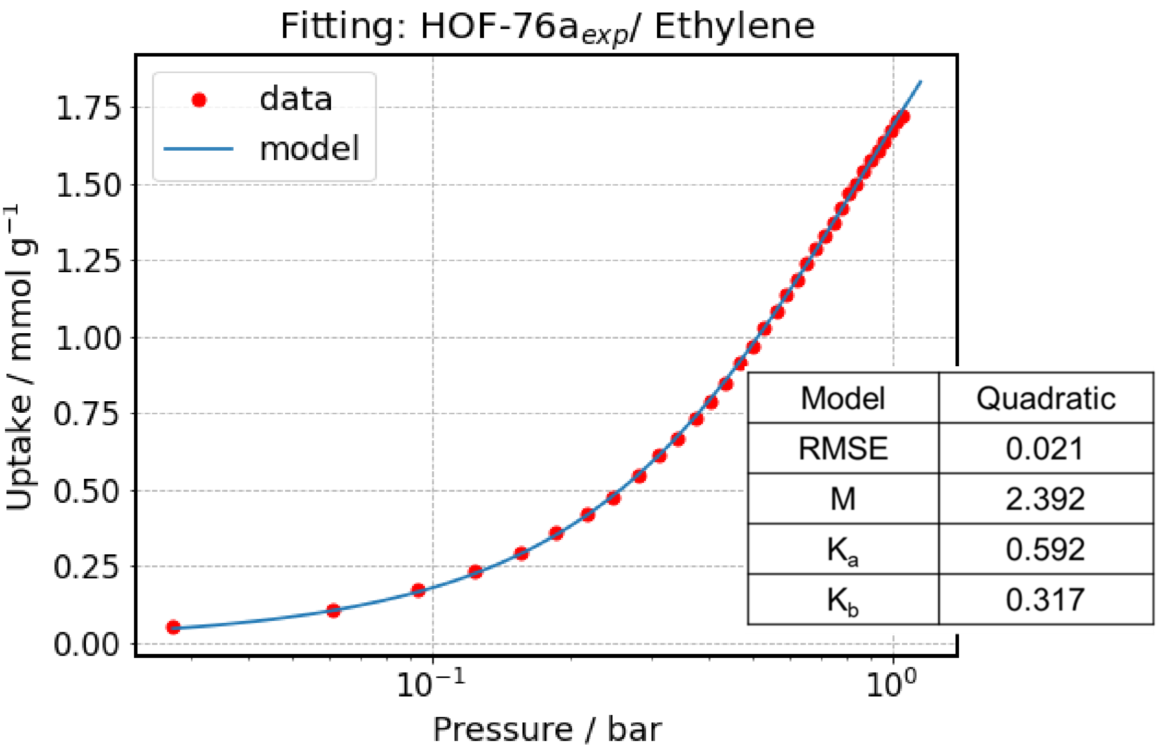

Figure S67. Model fit for experimental ethylene adsorption isotherm of HOF-76a.

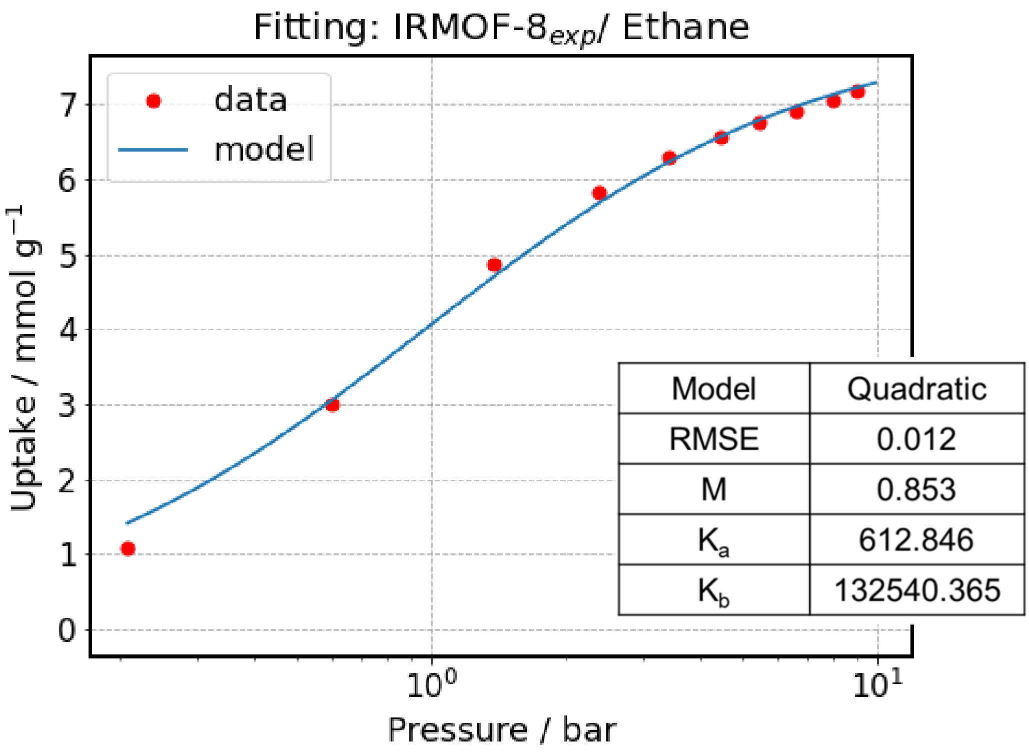

Figure S68. Model fit for experimental ethane adsorption isotherm of IRMOF-8.

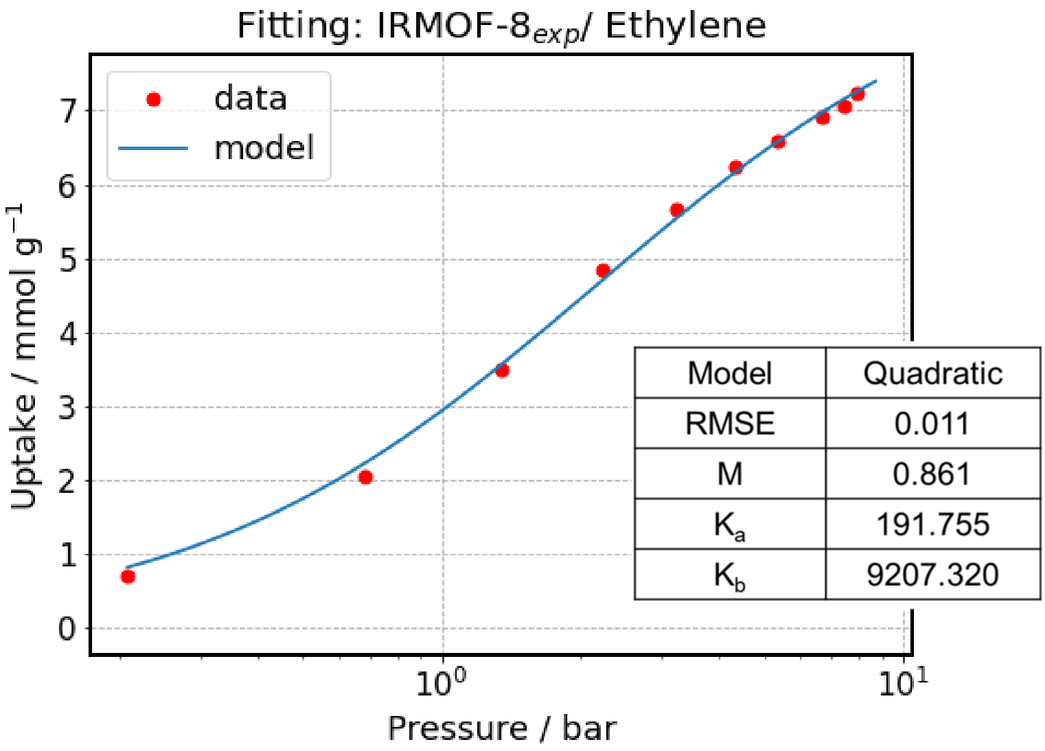

Figure S69. Model fit for experimental ethylene adsorption isotherm of IRMOF-8.

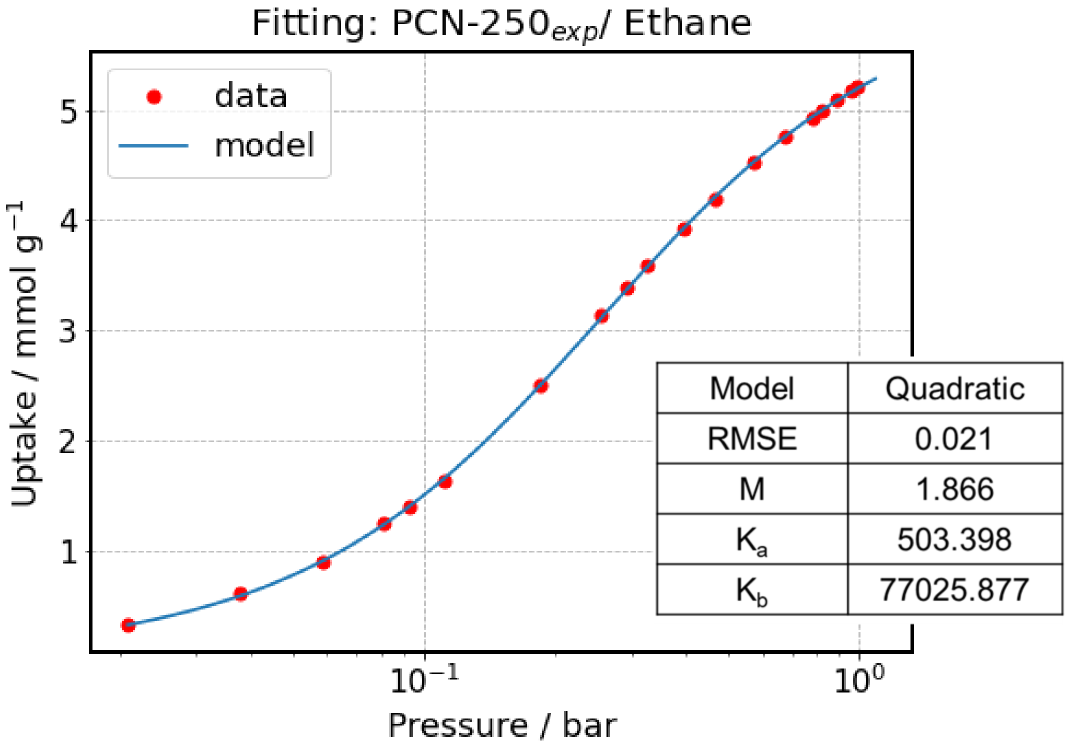

Figure S70. Model fit for experimental ethane adsorption isotherm of PCN-250.

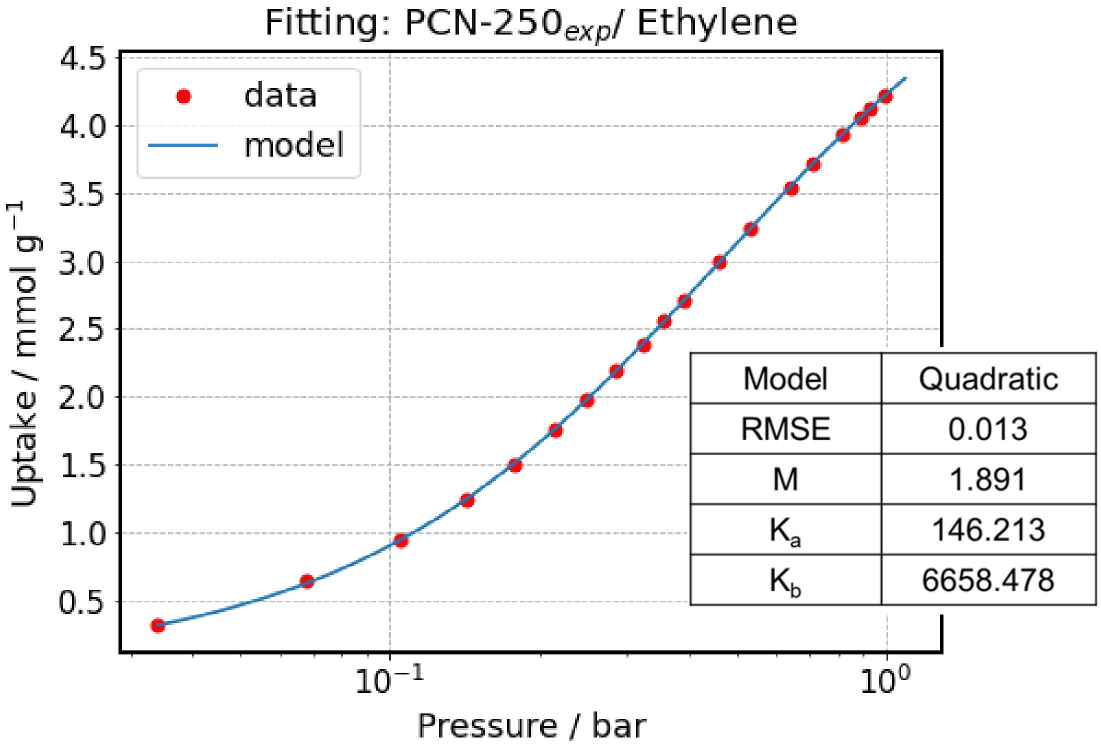

Figure S71. Model fit for experimental ethylene adsorption isotherm of PCN-250.

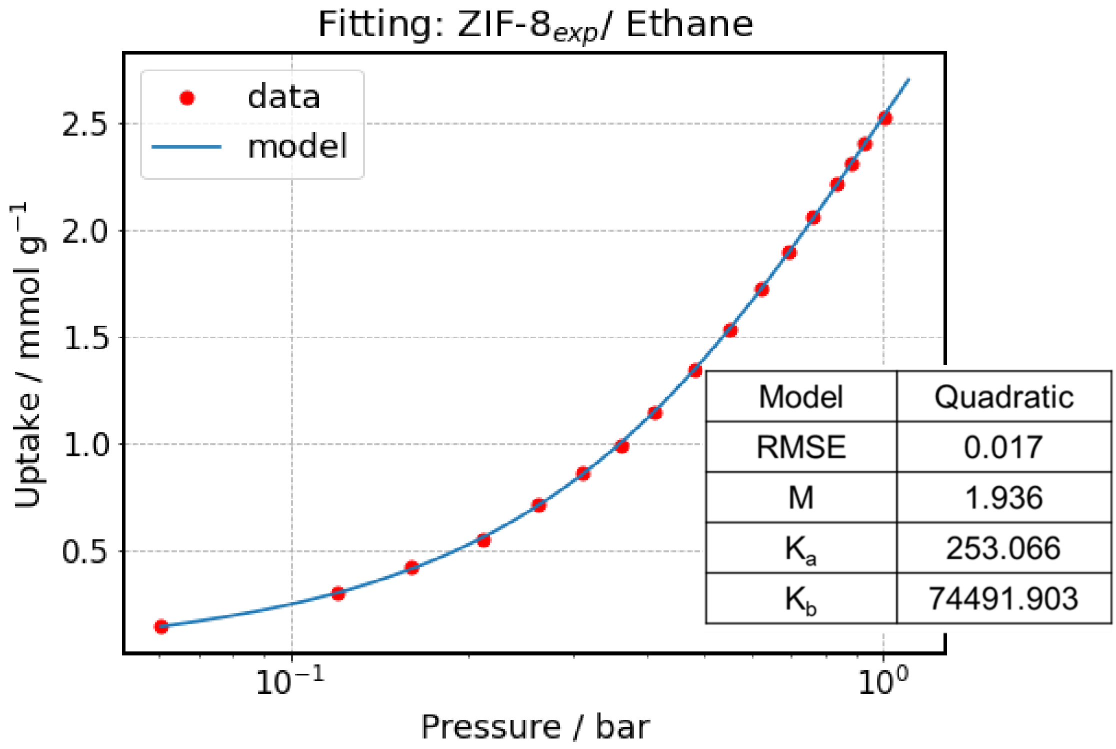

Figure S72. Model fit for experimental ethane adsorption isotherm of ZIF-8.

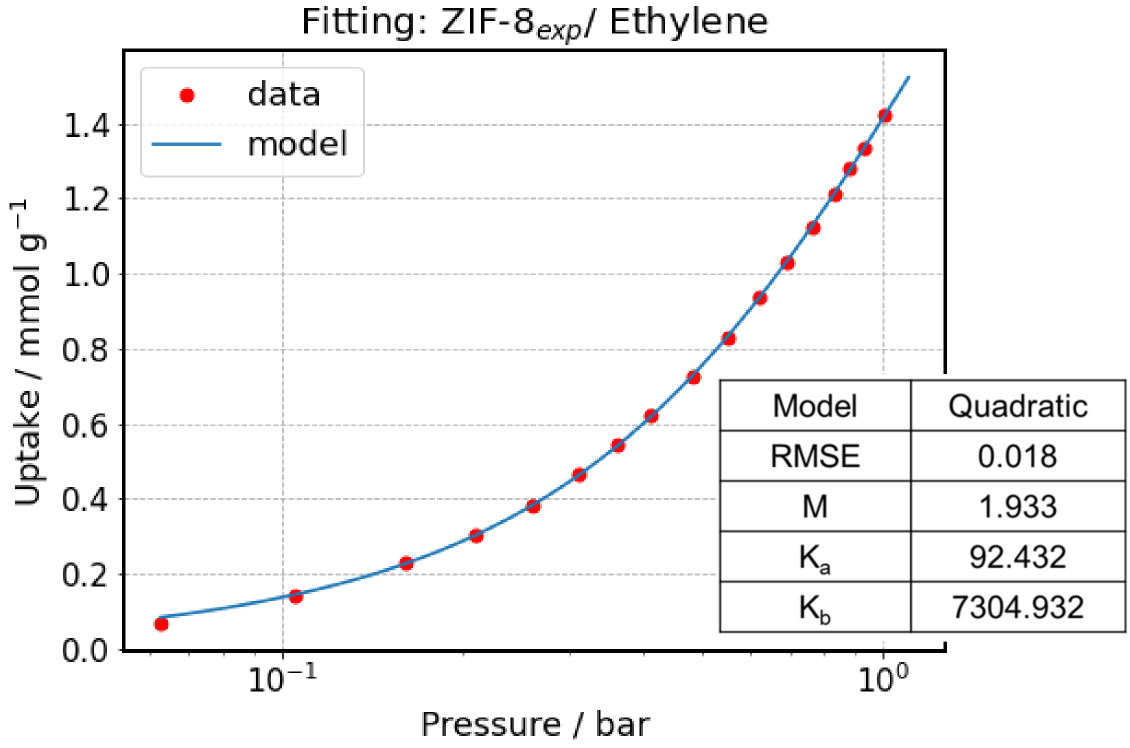

Figure S73. Model fit for experimental ethylene adsorption isotherm of ZIF-8.

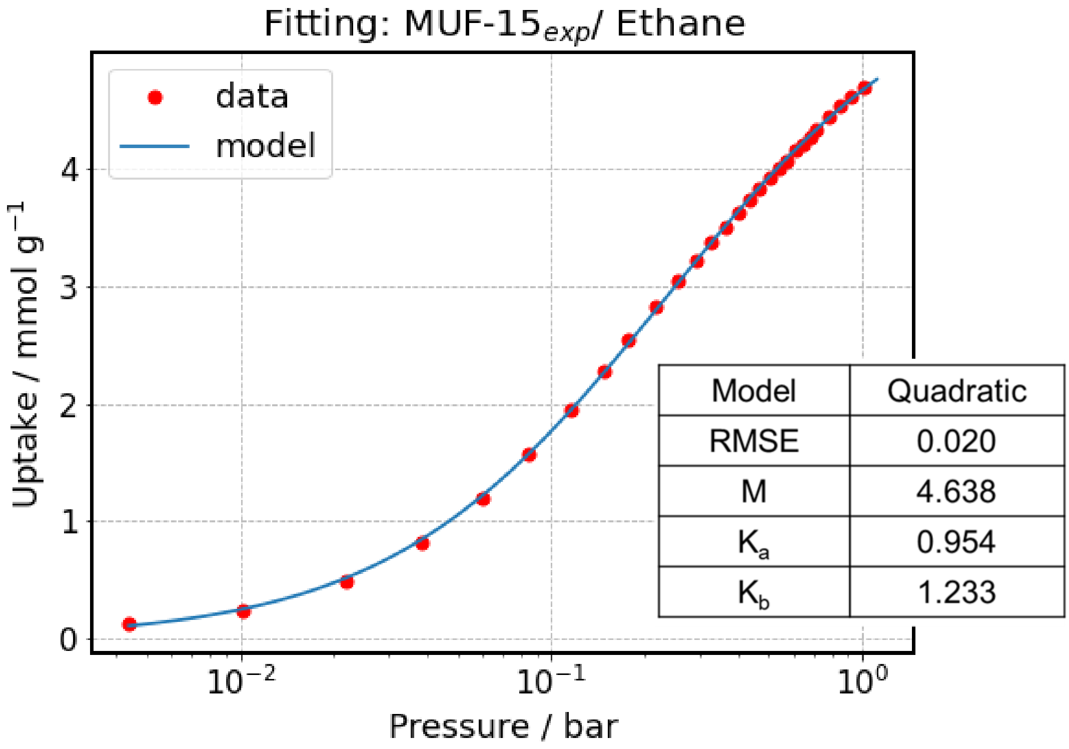

Figure S74. Model fit for experimental ethane adsorption isotherm of MUF-15.

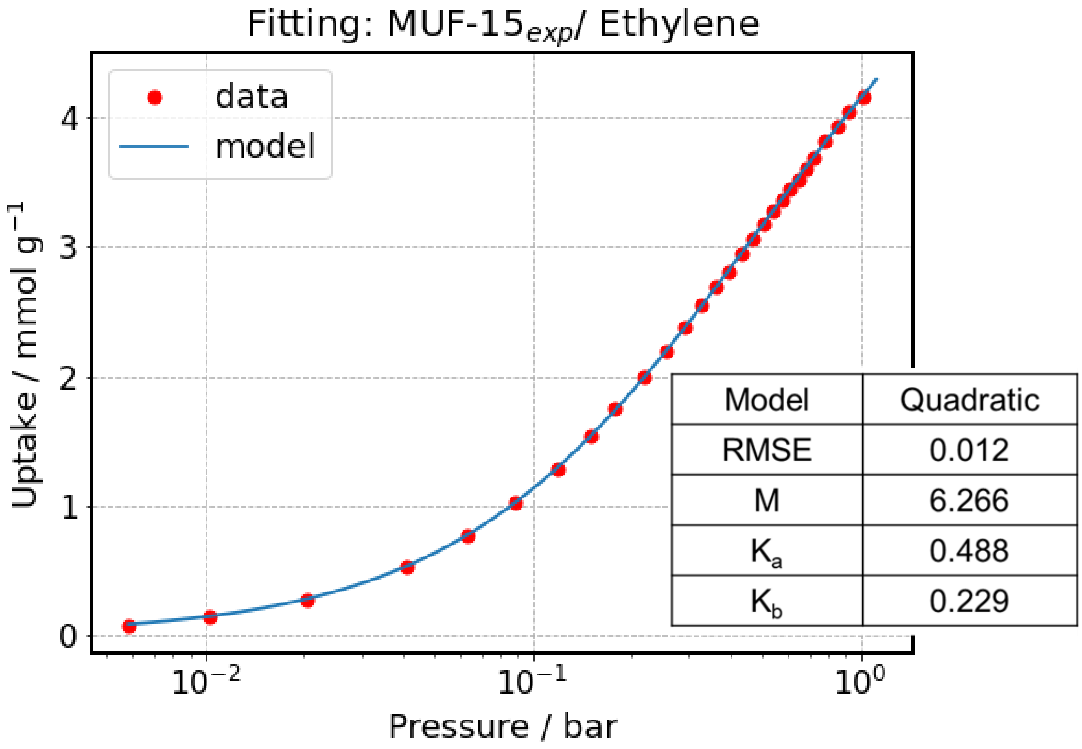

Figure S75. Model fit for experimental ethylene adsorption isotherm of MUF-15.

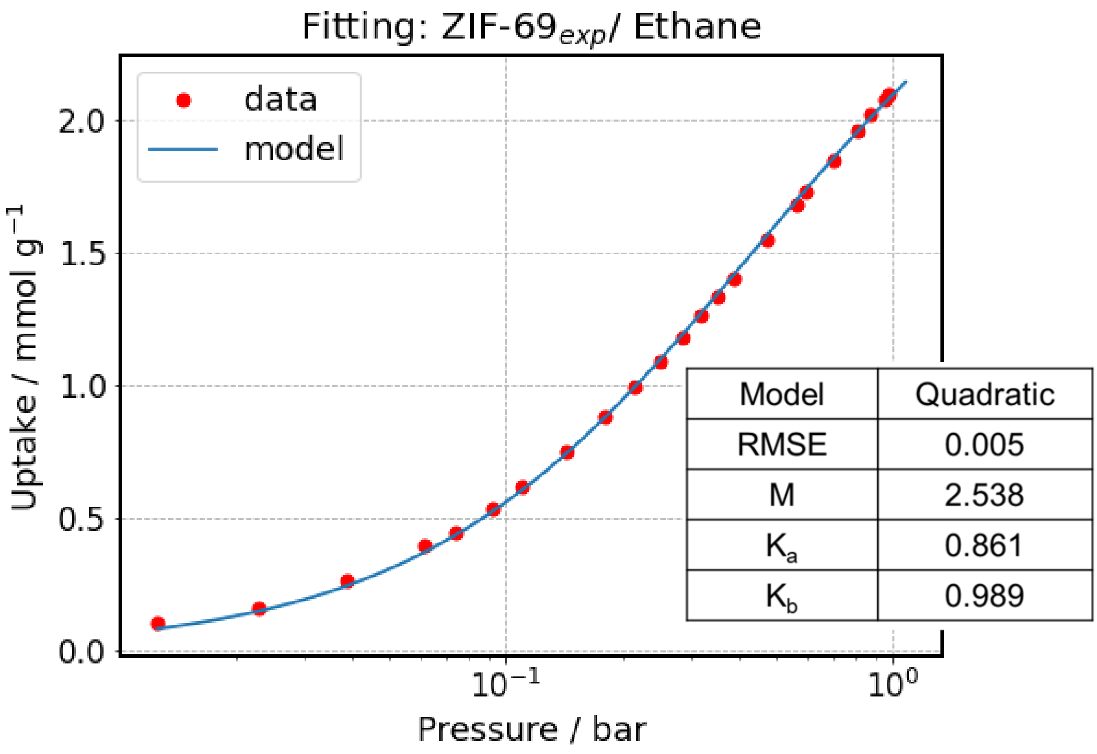

Figure S76. Model fit for experimental ethane adsorption isotherm of ZIF-69.

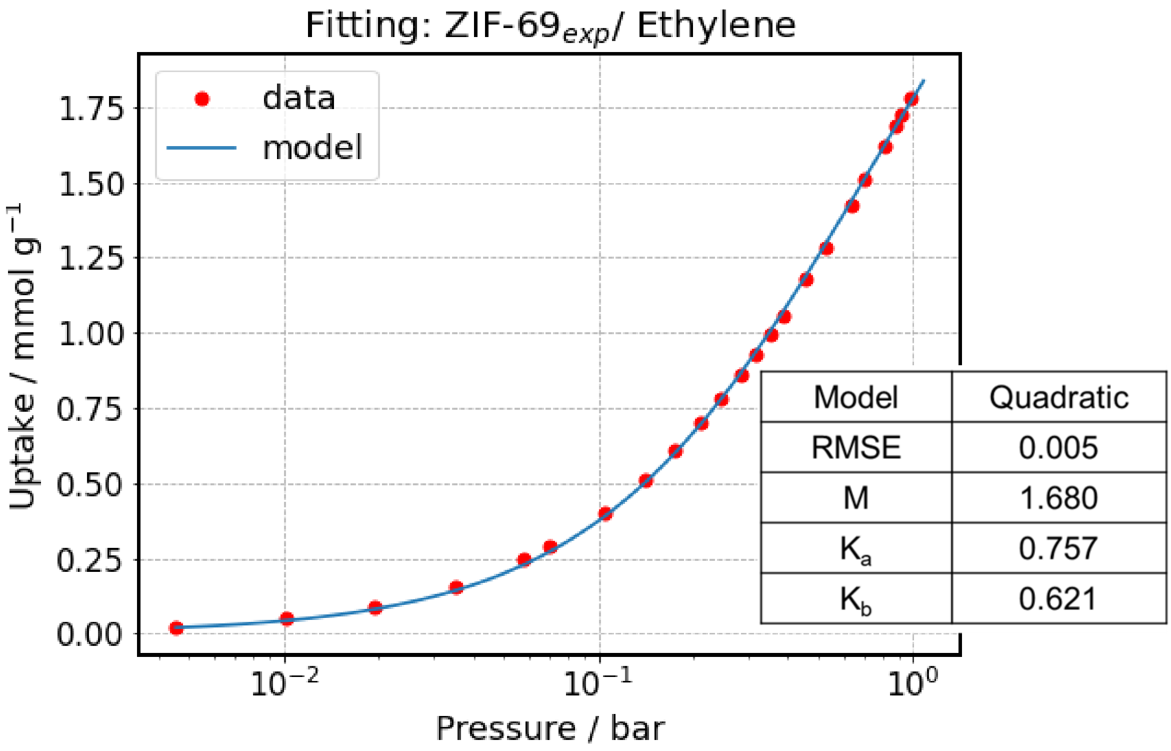

Figure S77. Model fit for experimental ethylene adsorption isotherm of ZIF-69.

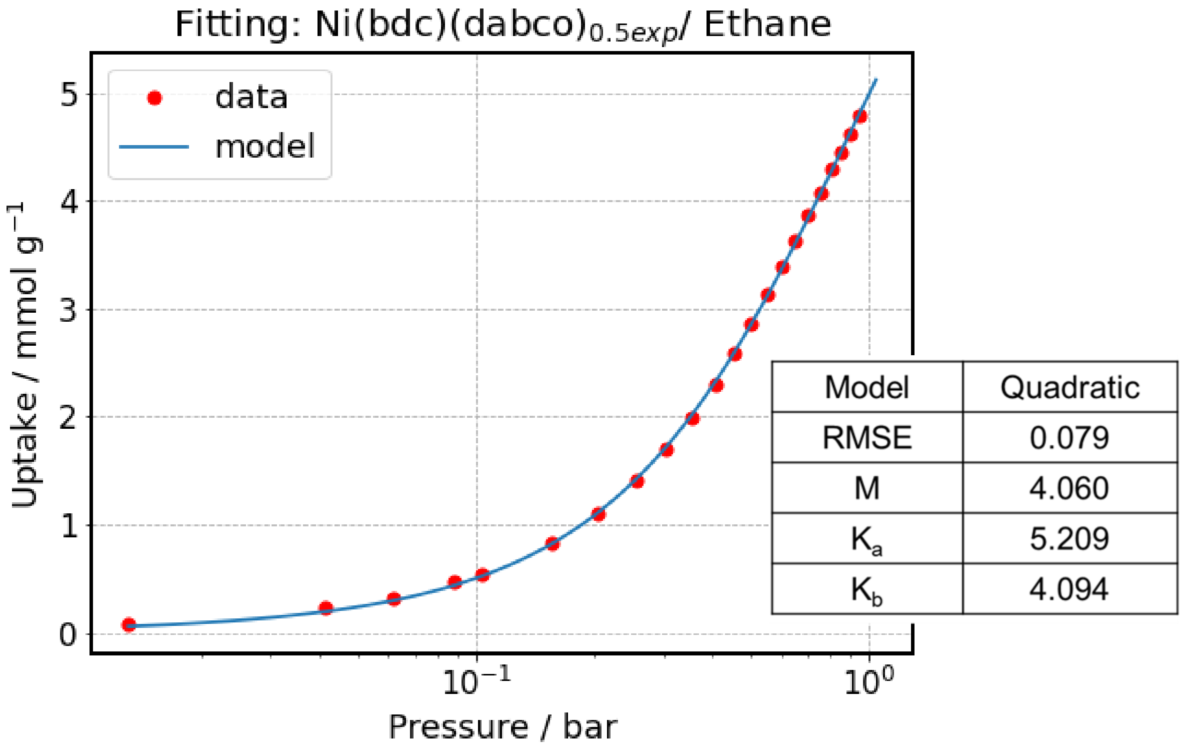

Figure S78. Model fit for experimental ethane adsorption isotherm of Ni(bdc)(dabco)$_{0.5}$.

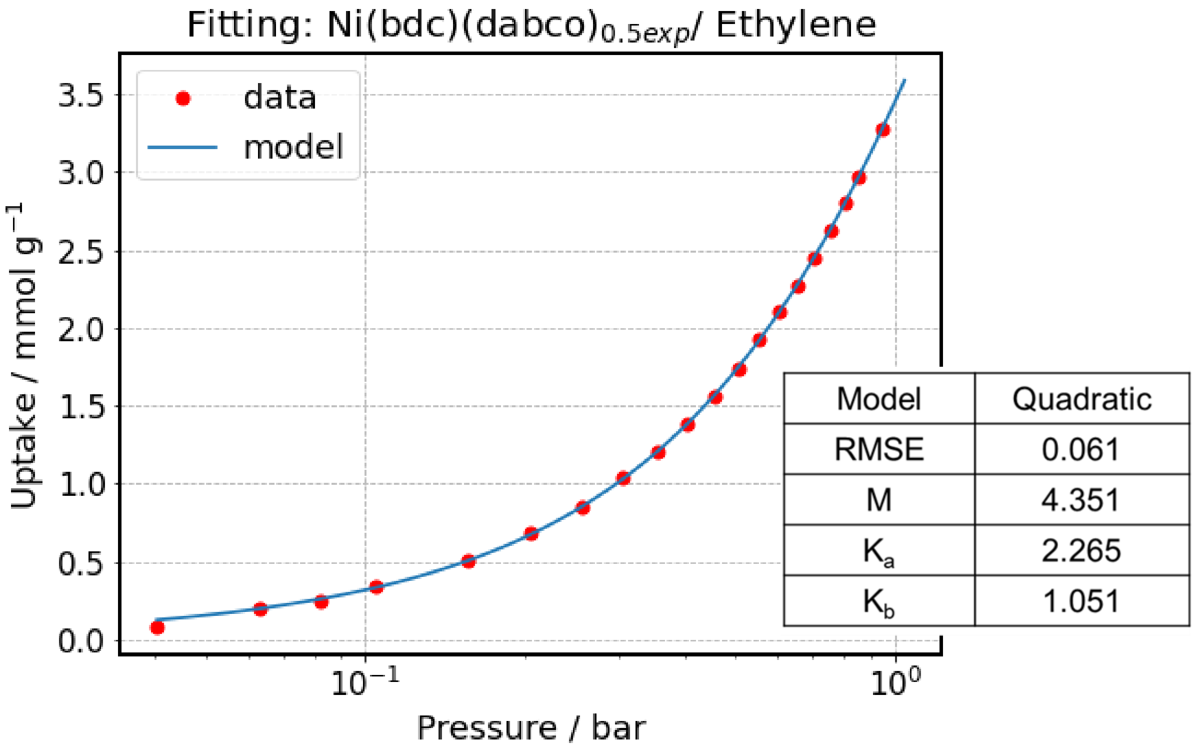

Figure S79. Model fit for experimental ethylene adsorption isotherm of Ni(bdc)(dabco)$_{0.5}$.

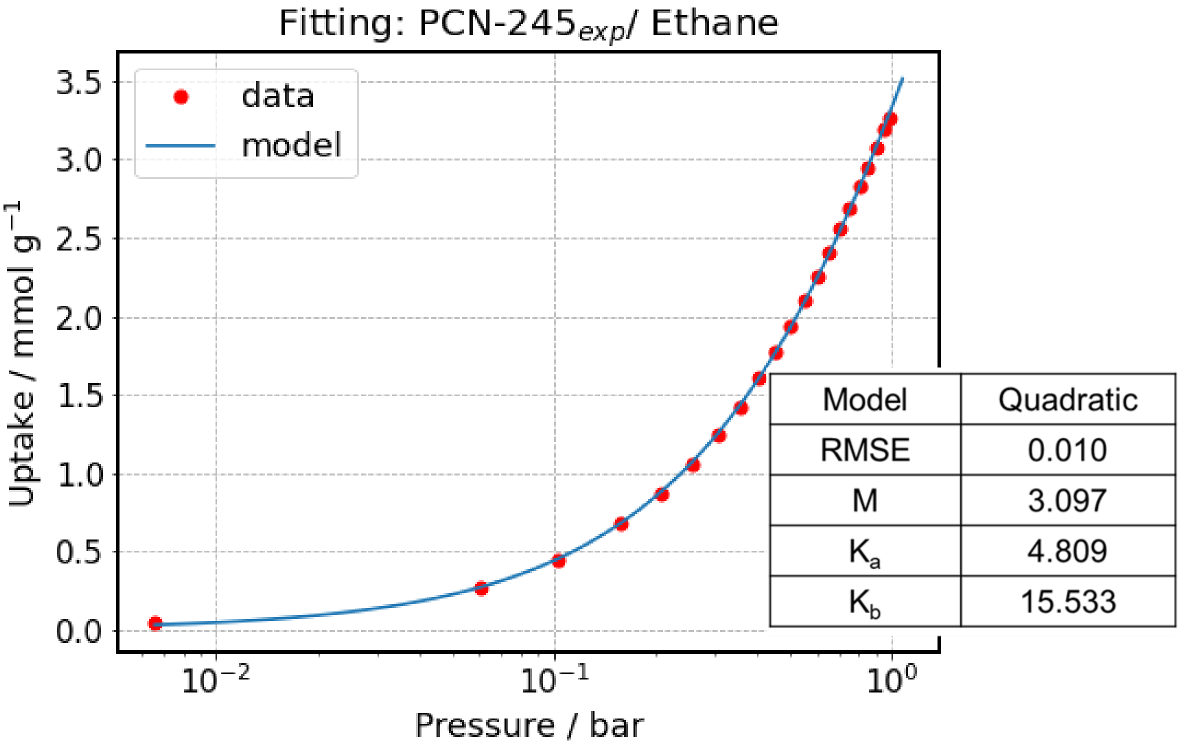

Figure S80. Model fit for experimental ethane adsorption isotherm of PCN-245.

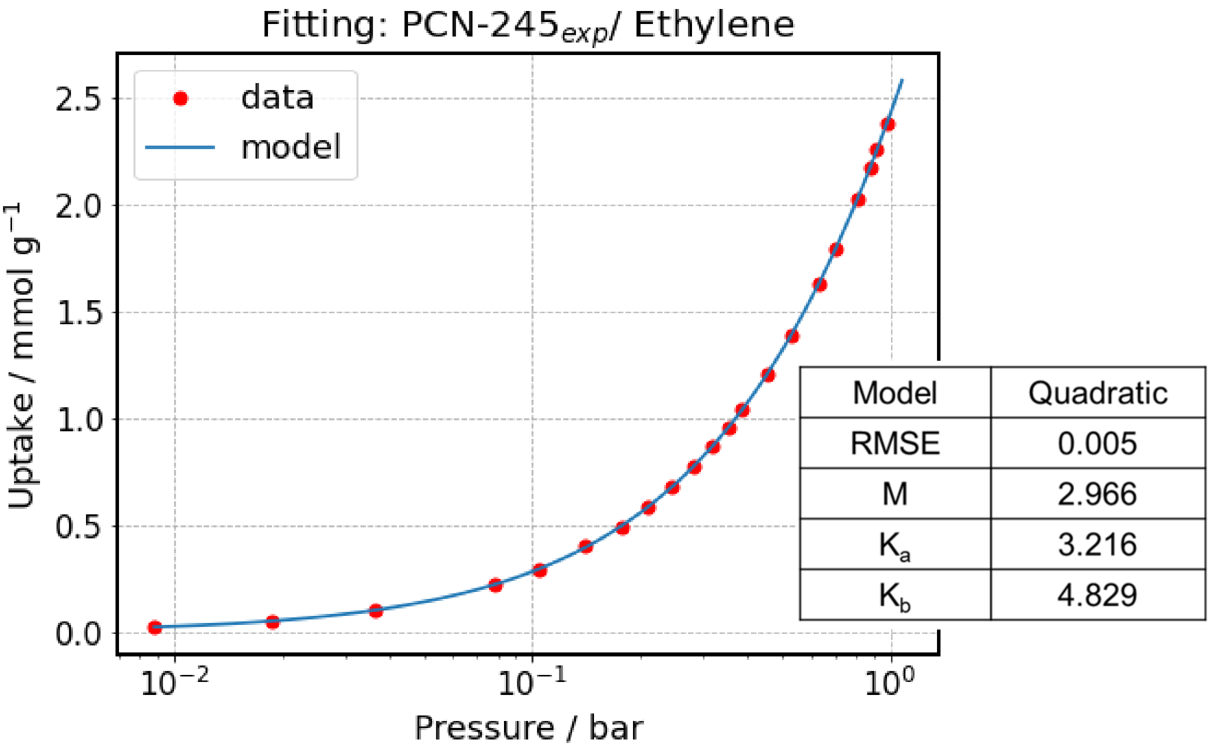

Figure S81. Model fit for experimental ethylene adsorption isotherm of PCN-245.

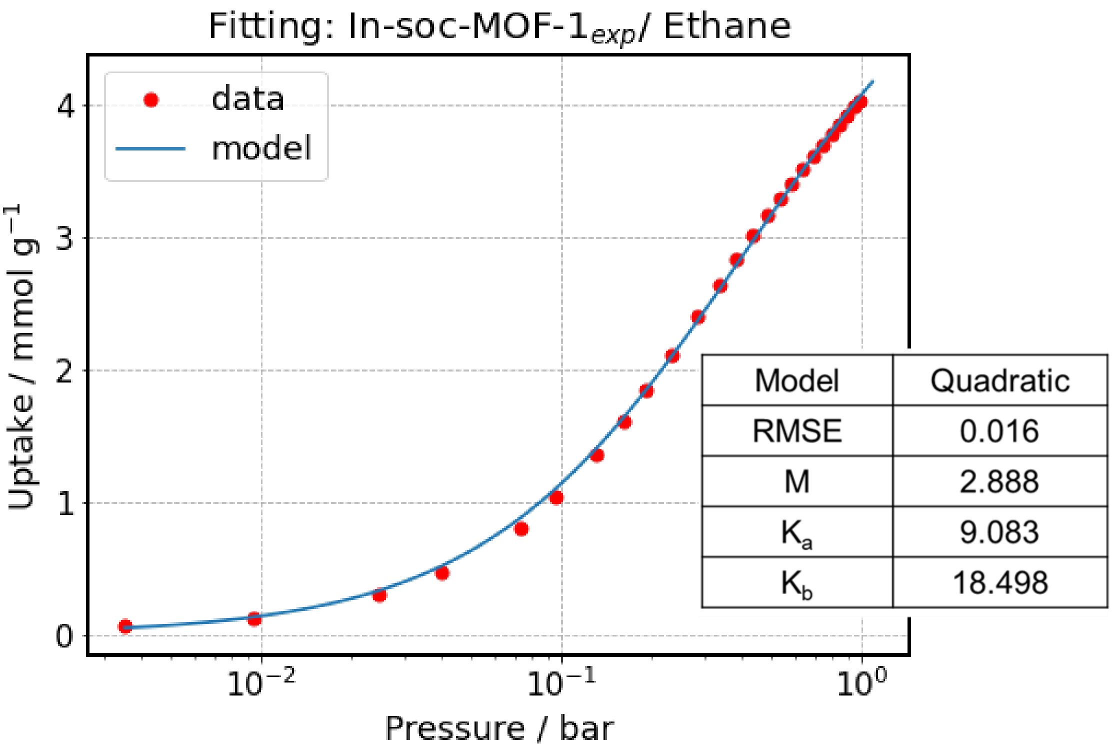

Figure S82. Model fit for experimental ethane adsorption isotherm of In-soc-MOF-1.

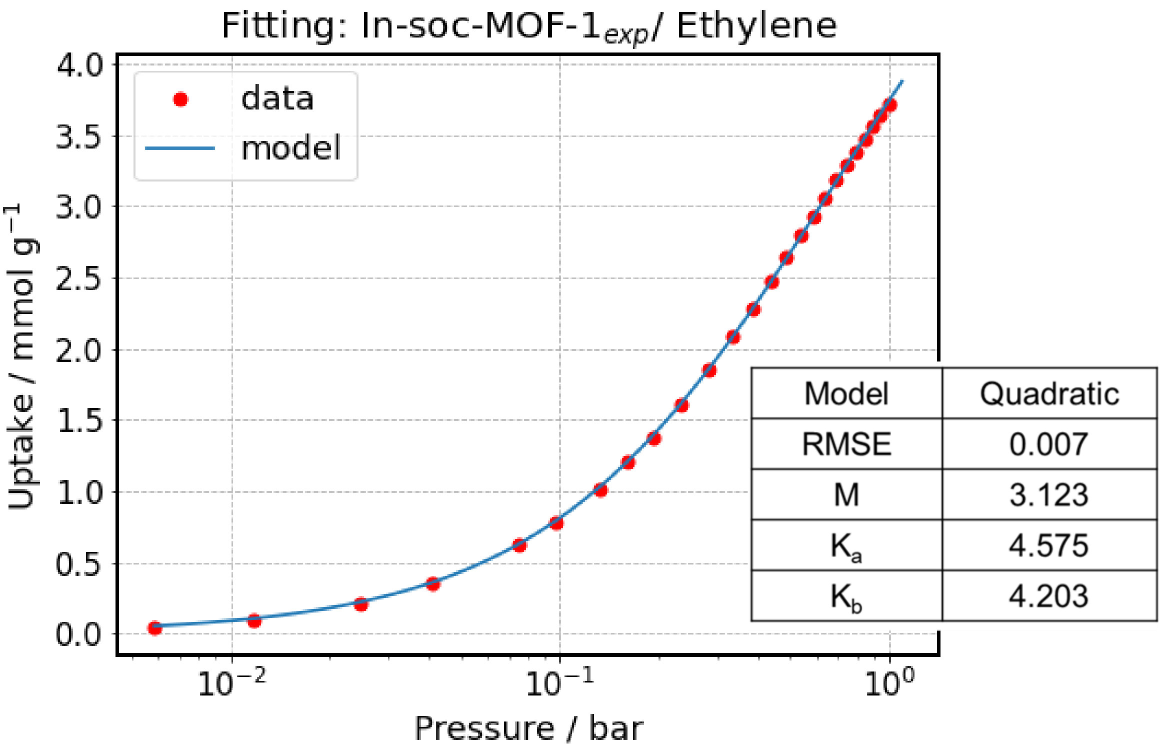

Figure S83. Model fit for experimental ethylene adsorption isotherm of In-soc-MOF-1.

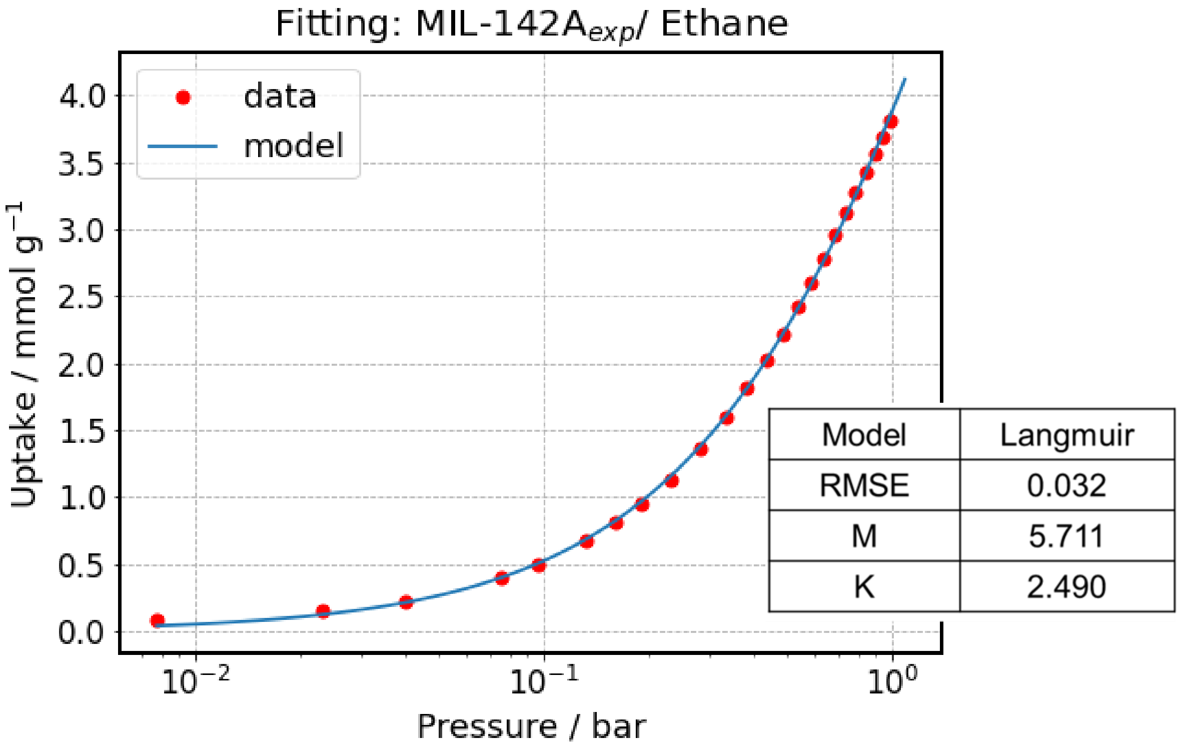

Figure S84. Model fit for experimental ethane adsorption isotherm of MIL-142A.

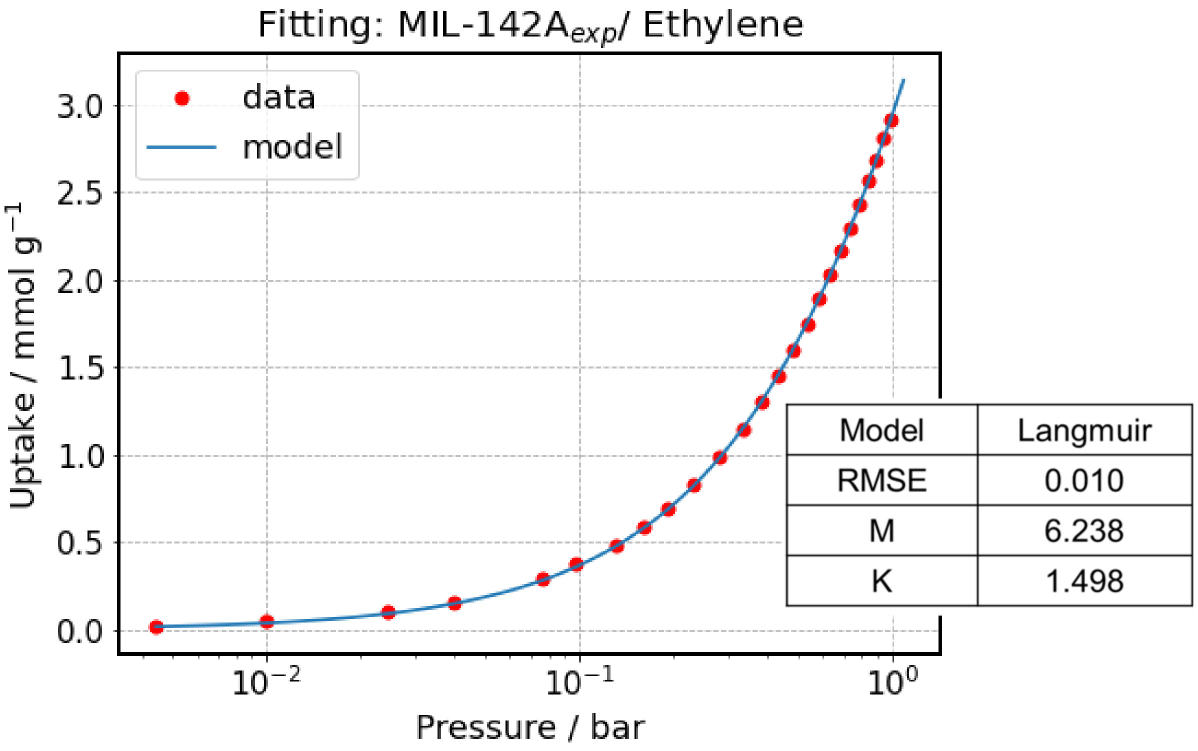

Figure S85. Model fit for experimental ethylene adsorption isotherm of MIL-142A.

S8. Comparison of productivity and recovery metrics

We obtained the productivity values that have been frequently proposed in the literatures. The results are shown from **Figure S86** to **S87**. For breakthrough simulations with the adsorbents we evaluated in this study, we used the following model equations, from Eq. S18 to Eq. S21. Based on the gas and solid phase concentrations, the model adopts linear driving force model to describe the mass transfer between the two phases. For different adsorbents, different isotherm models and corresponding parameters were applied in Eq. S20, which are listed in Section S7.

$$\frac{\partial C_i}{\partial t} = \frac{\partial(vC_i)}{\partial z} + \frac{\partial^2(D_{dis}C_i)}{\partial z^2} - \rho_s \frac{1-\varepsilon}{\varepsilon}\frac{\partial q_i}{\partial t} \quad \text{(Eq. S18)}$$

$$\frac{\partial q_i}{\partial t} = k_i(q_i^* - q_i) \quad \text{(Eq. S19)}$$

$$q_i^* = f_i^j(P_i, T) \quad \text{(Eq. S20)}$$

$$P_i = RTC_i \quad \text{(Eq. S21)}$$

where,

$i = \mathrm{C_2H_4, C_2H_6, He}$

$j = \mathrm{Adsorbents}$

t: time (sec)

z: position of column (m)

C_i: gas concentration ($mol \cdot m^{-3}$)

v: gas linear velocity ($m \cdot sec^{-1}$)

ε: column void fraction ($-$)

D_{dis}: dispersion coefficient

ρ_s: solid density

q_i: current solid phase uptake ($mol \cdot kg$)

q_i^*: equilibrium uptake ($mol \cdot kg$)

k_i: mass transfer coefficient ($1 \cdot sec^{-1}$)

P_i: partial pressure (Pa)

R: gas constant ($J \cdot mol^{-1} \cdot K^{-1}$)

T: temperature (K)

f_i^j: isotherm model (function of partial pressure and temperature)

The productivity values were obtained from the breakthrough simulations, where length (L) = 2 m (or 0.5 m for adsorbents with high uptake values), gas velocity (v)= 3 $mm \cdot sec^{-1}$, mass transfer coefficient

(k_{ov}) = 0.0001 sec^{-1}, solid density (ρ_s) = 1200 $kg \cdot m^{-3}$, feed composition ratio = 50:50. From each component flow rate of the breakthrough simulations, we use the following equation to obtain the the productivity:

$$(productivity) = \frac{\int_{t_1}^{t_2} f_{ethylene}(t)dt}{m_{solid}} \quad \text{(Eq. 22)}$$

To find the time period (t_1, t_2) in the above equation of productivity, we located the point where the accumulated amount of ethane becomes 0.001% of the ethylene amount.

Figures S86 and **S87** show the productivity obtained based on the simulated isotherm data and experimental isotherm data, respectively. Some materials have extremely small or zero productivity, which is likely due to the similarity in ethane and ethylene saturation uptake. For example, YUTDUO and GUMDEZ have ethane front in advance of the ethylene, and many of the adsorbents have significantly small productivity values less than 0.1. This is because the adsorbents with low productivity have similar saturated uptake values of ethane and ethylene. Since the ratio of two gas components is 50:50 in the breakthrough simulations, ethane and ethylene have similar breakthrough points when the same mass transfer coefficients are used for the simulation.

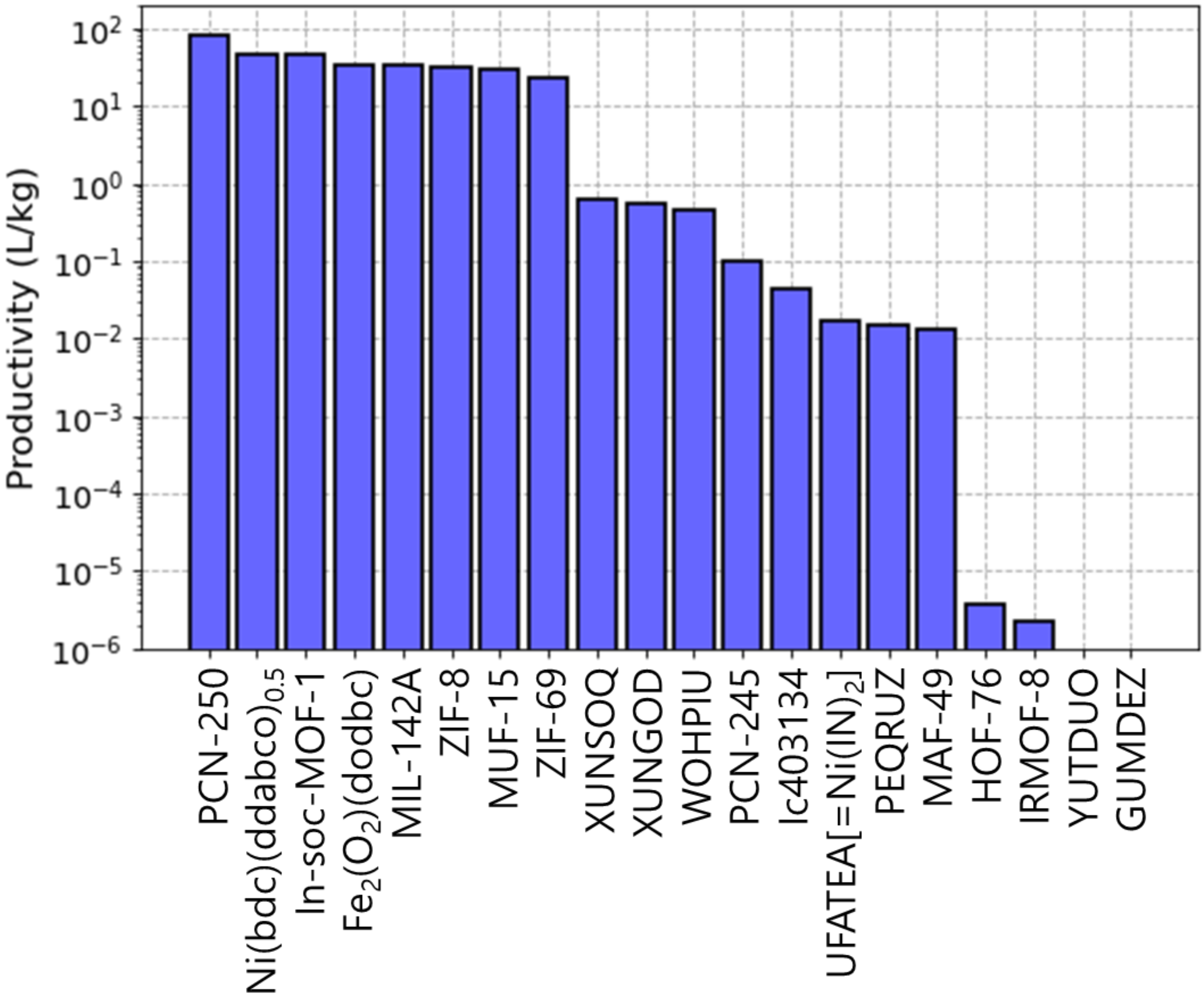

Figure S86. Productivity from the breakthrough simulations based on the simulated isotherm data (ethane comes first in YUTDUO and GUMDEZ cases due to the 50:50 feed condition).

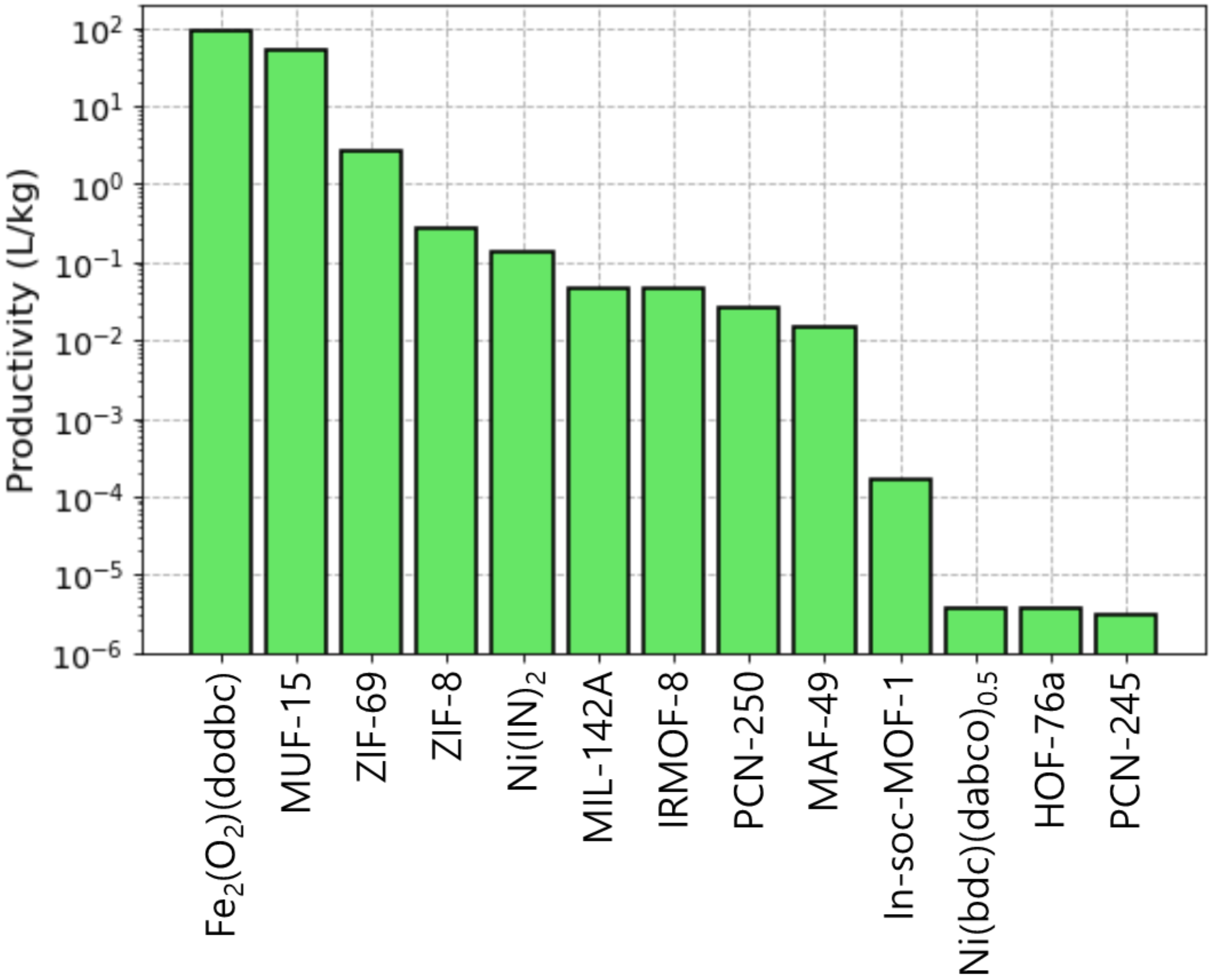

Figure S87. Productivity values from the breakthrough simulations based on the experimental isotherm data reported in other previous studies

However, the productivity values measured at different experimental conditions lead to different values and should not be compared with each other. For example, change in the feed concentration or column length leads to significant variance in adsorbent‘s productivity.

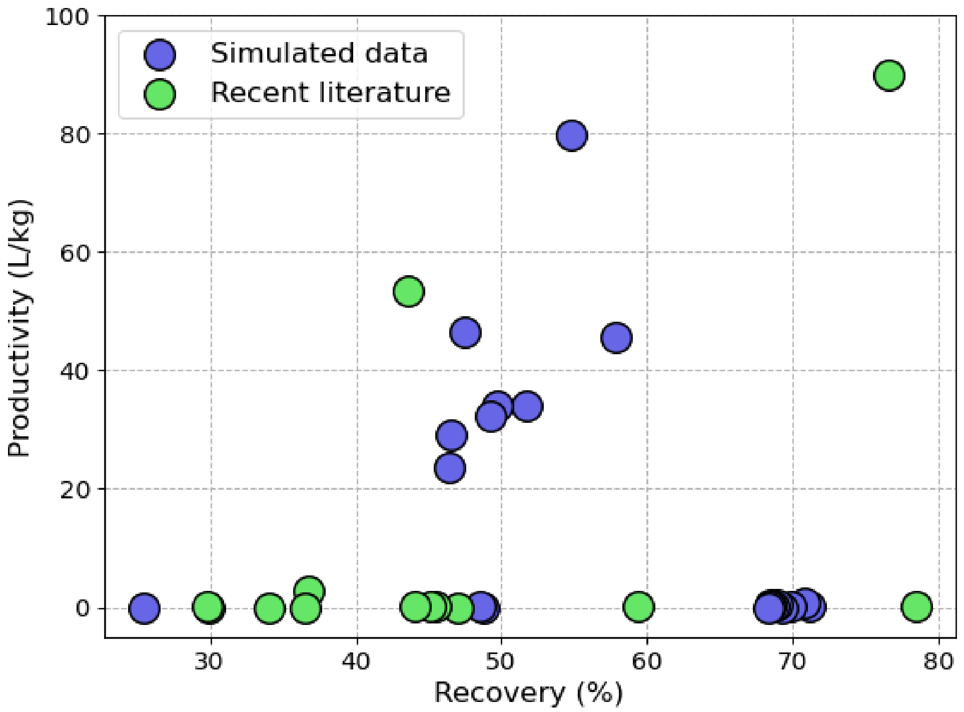

Figure S88. Scatter plot of the productivity value and the process recovery values for all the adsorbents used in this work.

Moreoever, the productivity values obtained from breakthrough experiments/simulations do not necessarily correlate with process-level metrics. To verify this, we compared the recovery values obtained from our ideal VSA simulations and productivity values (**Figure S88**). The spearman‘s ranking correlation coefficient (SRCC) value is 0.1023. The result indicates that the productivity value obtained from the breakthrough simulation/experiments are not necessarily related to process metric, such as product recovery, that we considered in this work. This is because the recovery from the VSA and the productivity from the breakthrough simulation (or experiment) represent completely different process information. In the breakthrough simulation (or experiments), the inert gas (typically helium) is employed to regenerate the adsorbents packed in advance of the column. However, in the actual operation of VSA process for ethane/ethylene separation, the inert gas is not injected because the use of inert gas increases the operating cost by causing to the loss both in the quality and the recovery of the ethylene due to the diluted gas concentration in the column. Moreover, the feed concentration is assumed to be 50:50 in a typical breakthrough work, while actual VSA processes have lower ethane concentration.

The product recovery metric tells us how much of ethylene can be produced out of the invested ethylene in the feed flow, which critically influences on the operating cost of the process. Computation of the metric reflects the effect of the remaining gas component after the desorption (i.e., regeneration) step, which impacts the adsorption characteristics of feed gas components in subsequent adsorption step.

[23] By repeating the adsorption-desorption cycles, the recovery value evaluates the adsorbents at the cyclic steady state (CSS) of the operation, which provides us with a more practical information about the perofrmance of adsorbents in the VSA process.

S9. References

[1] Z. Li, L. Du, J. Zhou, L. Li, Y. Hu, Y. Qiao, M. Xie, Q. Zhao, *New J. Chem.* **2013**, 37, 2473.

[2] S. Nandi, S. Collins, D. Chakraborty, D. Banerjee, P. K. Thallapally, T. K. Woo, R. Vaidhyanathan, *J. Am. Chem. Soc.* **2017**, 139, 1734.

[3] a) *ChemMaster - Microtrac BEL Corp.*, Microtrac BEL Corp., Japan **2019**; b) D. W. Kang, M. Kang, D. W. Kim, H. Kim, Y. H. Lee, H. Yun, J. H. Choe, C. S. Hong, *Adv. Sustainable Syst.* **2020**, DOI: 10.1002/adsu.2020001612000161.

[4] Y. G. Chung, E. Haldoupis, B. J. Bucior, M. Haranczyk, S. Lee, H. Zhang, K. D. Vogiatzis, M. Milisavljevic, S. Ling, J. S. Camp, B. Slater, J. I. Siepmann, D. S. Sholl, R. Q. Snurr, *J. Chem. Eng. Data* **2019**, 64, 5985.

[5] S. L. Mayo, B. D. Olafson, W. A. Goddard, *J. Phys. Chem.* **1990**, 94, 8897.

[6] C. D. Wick, M. G. Martin, J. I. Siepmann, *J. Phys. Chem. B* **2000**, 104, 8008.

[7] J. J. Potoff, J. I. Siepmann, *AlChE J.* **2001**, 47, 1676.

[8] D. Dubbeldam, S. Calero, D. E. Ellis, R. Q. Snurr, *Mol. Simul.* **2015**, 42, 81.

[9] D. Dubbeldam, S. Calero, T. J. H. Vlugt, *Mol. Simul.* **2018**, 44, 653.

[10] D. E. Coupry, M. A. Addicoat, T. Heine, *J. Chem. Theory Comput.* **2016**, 12, 5215.

[11] P. G. Boyd, S. M. Moosavi, M. Witman, B. Smit, *J. Phys. Chem. Lett.* **2017**, 8, 357.

[12] S. Plimpton, *J. Comput. Phys.* **1995**, 117, 1.

[13] *F Module - Accelrys Inc.*, Accelrys Inc, San Diego, CA **2011**.

[14] T. F. Willems, C. H. Rycroft, M. Kazi, J. C. Meza, M. Haranczyk, *Microporous Mesoporous Mater.* **2012**, 149, 134.

[15] J. Hutter, M. Iannuzzi, F. Schiffmann, J. VandeVondele, *Wiley Interdiscip. Rev.: Comput. Mol. Sci.* **2014**, 4, 15.

[16] J. P. Perdew, K. Burke, M. Ernzerhof, *Phys. Rev. Lett.* **1996**, 77, 3865.

[17] S. Grimme, J. Antony, S. Ehrlich, H. Krieg, *J. Chem. Phys.* **2010**, 132, 154104.

[18] a) C. Hartwigsen, S. Goedecker, J. Hutter, *Phys. Rev. B* **1998**, 58, 3641−3662; b) S. Goedecker, M. Teter, J. Hutter, *Phys. Rev. B* **1996**, 54, 1703.

[19] J. VandeVondele, J. Hutter, *J. Chem. Phys.* **2007**, 127, 114105.

[20] S. Ga, H. Jang, J. H. Lee, *Comput. Chem. Eng.* **2017**, 102, 188.

[21] J.-H. Park, S.-S. Han, J.-N. Kim, S.-H. Cho, *Korean J. Chem. Eng.* **2004**, 21, 236.

[22] C. M. Simon, B. Smit, M. Haranczyk, *Comput. Phys. Commun.* **2016**, 200, 364.

[23] S. Ga, S. Lee, J. Kim, J. H. Lee, *Comput. Chem. Eng.* **2020**, 143, 107105.
